# Upstream Ecological Control of the IAA–Skatole Branch: A pH-Dependent Triple-Lock Framework for Gut-Derived Uremic Toxin Precursors

**DOI:** 10.3390/toxins18090362

**Published:** 2026-08-24

**Authors:** Kana Yuasa, Hidehisa Shimizu

**Affiliations:** 1 Graduate School of Natural Science and Technology, Shimane University, 1060 Nishikawatsu-Cho, Matsue 690-8504, Shimane, Japan; 2 Faculty of Life and Environmental Sciences, Shimane University, 1060 Nishikawatsu-Cho, Matsue 690-8504, Shimane, Japan; 3 Institute of Agricultural and Life Sciences, Academic Assembly, Shimane University, 1060 Nishikawatsu-Cho, Matsue 690-8504, Shimane, Japan; 4 The United Graduate School of Agricultural Sciences, Tottori University, 4-101 Koyama-Minami, Tottori 680-8553, Tottori, Japan; 5 Estuary Research Center, Shimane University, 1060 Nishikawatsu-Cho, Matsue 690-8504, Shimane, Japan; 6 Interdisciplinary Center for Science Research, Shimane University, 1060 Nishikawatsu-Cho, Matsue 690-8504, Shimane, Japan

**Keywords:** skatole, indole-3-acetic acid, indoleacetate decarboxylase, microbial tryptophan metabolism, intestinal pH, gut–kidney axis, uremic toxins, equifinality, mathematical modeling

## Abstract

Gut-derived indole metabolites are implicated in the gut–kidney axis, but the factors controlling the intestinal conversion of indole-3-acetic acid (IAA) to skatole remain incompletely defined. We developed a deterministic, hypothesis-generating framework that represents this conversion as a finite-pool allocation process governed by pH-dependent ecological permissiveness, terminal-conversion capacity, precursor availability, spatial progression, and competing loss. The model separates the available-pool scale from a dimensionless integrated conversion exposure, Ψ. Across 5400 loss-free scenarios spanning 10 pH profiles and graded metabolic and host-associated constraints, the distal endpoint normalized to the available pool followed the analytically derived relationship $1-\exp\left(-\Psi \right)$. Thus, distinct combinations of mechanistically relevant parameters produced the same normalized distal endpoint, demonstrating that this endpoint alone cannot uniquely identify the underlying mechanism. Competing loss further separated absolute, total-pool-normalized, and conditional outputs, showing that mechanistic interpretation depends on endpoint normalization. Analytical and numerical checks supported internal consistency. The framework was not fitted to biological data, and concentration values were used only as technical scaling references. This biologically unvalidated model generates experimentally testable hypotheses regarding the roles of pH, terminal-conversion capacity, precursor availability, and competing loss in intestinal IAA-to-skatole metabolism; it is not intended to provide physiological or clinical predictions.

## 1. Introduction

Chronic kidney disease (CKD) imposes a growing global burden through premature mortality, cardiovascular complications, and kidney failure. In 2023, approximately 788 million adults worldwide had CKD, corresponding to an age-standardized prevalence of 14.2% [1]. Progressive nephron loss is accompanied by inflammation, oxidative stress, tubular injury, extracellular matrix accumulation, and renal fibrosis [2,3]. The generation and systemic accumulation of gut-derived protein-bound uremic toxins (PBUTs) are influenced by microbial function, host factors, intestinal transit, diet, the intestinal environment, and renal clearance [4,5,6,7,8,9,10]. As kidney function declines, PBUTs accumulate, while their strong protein binding limits removal by conventional dialysis [7,8,9,10]. Reducing intestinal toxin-precursor production before absorption may therefore provide a complementary strategy for limiting systemic exposure.

The gut–kidney axis integrates diet, microbial metabolism, epithelial barrier function, and renal clearance. CKD-associated intestinal alterations include disrupted microbial function, reduced beneficial fermentation products, barrier impairment, inflammation, and altered aromatic amino acid metabolism and uremic-solute generation [4,5,6,7,8,9]. However, these signatures are not attributable solely to kidney dysfunction. Intestinal transit, medications, dietary substrate availability, dialysis modality, and intestinal inflammation also influence microbial and metabolite profiles [5,6]. Lower estimated glomerular filtration rate has been associated with increased indole- and p-cresol-biosynthetic potential and a shift from saccharolytic toward proteolytic metabolism, with intestinal transit and nutrition- and medication-related factors acting as important covariates [5,6]. Microbiome-targeted interventions must therefore account for CKD stage, dialysis status, intestinal transit and inflammation, medications, and renal dietary restrictions.

Among PBUTs, circulating indoxyl sulfate (IS) has been associated with adverse CKD outcomes and pathways involving vascular injury, coagulation, inflammation, cellular senescence, and renal fibrosis [11,12,13,14,15]. IS and other tryptophan-derived uremic solutes can activate the aryl hydrocarbon receptor (AhR), although their effects depend on ligand identity, concentration, cellular context, exposure duration, and renal clearance [12,13,14,15]. Microbial tryptophan metabolism comprises distinct biochemical pathways [16,17,18]. Tryptophanase converts tryptophan to indole, which is absorbed and hepatically oxidized and sulfated to form IS [16,17,18]. Other pathways generate indole-3-acetic acid (IAA), indole-3-aldehyde, indolelactic acid, indolepropionic acid, indoleacrylic acid, and related metabolites with context-dependent effects on mucosal immunity, epithelial barrier function, and inflammatory signaling [16,17,18,19,20,21,22,23,24,25]. Locally generated IAA may participate in intestinal signaling, whereas circulating IAA may contribute to the uremic solute burden when renal clearance is impaired. Microbial branch allocation must therefore be distinguished from subsequent absorption, host metabolism, and systemic disposition.

The IAA–skatole branch is biologically relevant because its metabolites can elicit distinct and sometimes opposing responses. In Caco-2 cells, IAA exerted antiproliferative effects associated with Toll-like receptor 4 (TLR4)–c-Jun N-terminal kinase (JNK) signaling [26] and counteracted skatole-induced proliferation in HCT-116 cells [27]. IAA and skatole also exerted opposing effects on multidrug resistance protein 1 (MDR1) homeostasis [28], while orally administered IAA produced intestinal and systemic effects in a dextran sulfate sodium-induced colitis model [29]. Conversely, skatole altered intestinal epithelial functions through AhR- and p38-associated responses [30], induced tumor necrosis factor-α (TNF-α) through p38 and JNK signaling that was partially attenuated by AhR activation [31], and promoted inflammatory responses involving nuclear factor-κB (NF-κB), extracellular signal-regulated kinase (ERK), p38, TNF-α, and interleukin-6 (IL-6) [32].

In a related but biochemically distinct context, circulating IS has been implicated in CKD-associated colorectal cancer through increased epidermal growth factor receptor (EGFR) expression, enhanced sensitivity to epidermal growth factor (EGF), and AhR-, Akt/β-catenin-, and c-Myc-related proliferative signaling [33,34,35]. In HCT-116 cells, IS-induced EGFR upregulation was accompanied by enhanced responsiveness to subsequent EGF stimulation [33,34]. These findings provide translational motivation for controlling upstream microbial metabolism but do not establish that the IAA-to-skatole branch regulates the indole-to-hepatic IS pathway. Microbial indole production, intestinal absorption, hepatic IS formation, EGF sensitivity, and downstream IS-associated signaling are outside the present model.

The IAA–skatole branch provides a tractable system for examining environmental regulation of microbial metabolic allocation. Foundational studies characterized microbial IAA and skatole production and the biological and toxicological properties of skatole [36,37,38,39]. IAA-to-skatole conversion has been demonstrated in anaerobic isolates and microbial communities [40,41]. In indoleacetate decarboxylase (IAD)-positive anaerobes, IAA is the direct skatole precursor, and the oxygen-sensitive glycyl radical enzyme IAD catalyzes terminal decarboxylation [42] through hydrogen-atom transfer [43]. Indole and skatole have been measured in bacterial cultures, intestinal contents, and fecal samples, with skatole production documented in mixed fecal communities [44,45]. Elevated fecal skatole concentrations have also been reported in patients with large-bowel polyps or colorectal cancer [46].

The microbial indole-to-hepatic IS and IAA-to-skatole pathways are therefore biochemically distinct. This study addresses only allocation within a simplified microbial IAA–skatole branch and does not simulate indole production, hepatic IS formation, intestinal absorption, renal clearance, or circulating IS. We designate the fraction remaining on the precursor side as the “retained IAA-equivalent” and that assigned to terminal skatole formation as the “skatole-equivalent.” These are model-derived allocation quantities, not measured concentrations, and do not imply that all non-converted material remains chemically intact as IAA. The model does not calculate receptor activation, EGF sensitivity, intracellular signaling, inflammation, epithelial injury, proliferation, or therapeutic efficacy.

At the community level, terminal conversion cannot be inferred from enzyme abundance alone. Fermentable-carbohydrate availability redirects microbial tryptophan metabolism, whereas luminal pH modulates tryptophan-catabolic activity [47,48,49]. High-protein and high-meat-protein diets can favor proteolytic fermentation [49,50,51], while dietary substrates broadly shape microbial composition, carbohydrate fermentation, short-chain fatty acid (SCFA) production, and colonic physiology [51,52,53,54,55,56]. Resistant starch and other fermentable substrates can acidify the lumen and modify intestinal tumor-related outcomes in experimental systems [55,56,57,58]. Butyrate-associated responses and resistant-starch-rich diets have been linked to lower intestinal or tissue skatole burdens [59,60], whereas dietary fiber, lactose, and non-starch polysaccharides can alter indole and skatole production or absorption [61,62]. Increased carbohydrate fermentation may thus favor butyrate-producing networks over proteolytic metabolism [53,54,55,56,63]. Because much of this evidence derives from animal and fermentation studies rather than CKD cohorts, the framework treats pH as an integrative ecological variable reflecting community-level permissiveness for substrate utilization and terminal conversion, rather than as a proxy for the abundance or activity of a single enzyme. Intestinal and fecal pH vary with anatomical location, diet, transit, and health status [64,65]. The colonic mucus system restricts microbial access to the epithelium and influences local substrate availability [66,67], while environmental pH adaptation may promote community stability [68]. These observations motivate a three-layer conceptual and scenario-based framework rather than a unified kinetic mechanism. The Upstream Ecological Lock represents permissiveness associated with luminal pH and fermentable-substrate availability; the Terminal Metabolic Lock represents IAD-associated conversion capacity; and the Host-Protection Lock conceptually represents reduced availability of host-associated and luminal substrates to the modeled microbial branch. Potential contributors to the latter include preserved epithelial and mucus-barrier integrity and reduced microbial access to endogenous tryptophan-containing material. These mechanisms are not explicitly simulated. The Host-Protection Lock was represented operationally by the dimensionless substrate-availability coefficient $L_{3}$, which determines the fraction of the model-defined total precursor-equivalent pool available to enter the spatial conversion system. Because the layers operate at distinct biological scales, they are modeled as separate scenario variables rather than as components of a shared kinetic mechanism. A phenomenological loss compartment represents absorption, degradation, or diversion into alternative pathways.

The feasibility of upstream pathway control is supported by mechanism-based inhibition of microbial tryptophanases, which reduced circulating IS in preclinical models [69], and rational genetic manipulation of microbial metabolism, which modulated a circulating uremic solute [70]. Although these studies neither examine IAD-mediated conversion nor validate the present model, they demonstrate that selective manipulation of microbial precursor pathways can alter host exposure.

To our knowledge, no quantitative framework has systematically examined how ecological permissiveness, terminal-conversion capacity, and host-associated substrate availability jointly influence allocation within a simplified IAA–skatole branch. Principal uncertainties include model structure, parameter assumptions, scaling conventions, the consequences of assumed pH deviations near the transition region, and robustness across alternative pH profiles and conversion capacities. The nominal total-pool scale was informed by reported skatole measurements in microbial cultures, intestinal contents, and fecal samples [38,44,45,46]. Conversion to an aqueous-equivalent scale required assumptions regarding fecal density and the water-accessible fraction. Because IAA and skatole are chemically distinct, fecal and luminal concentrations are not interchangeable, and terminal-metabolite burden cannot directly indicate precursor concentration. Thus, 1000 μM is used solely as a technical scaling benchmark, not as an established physiological luminal IAA concentration. Outputs represent scenario-dependent equivalents rather than empirically calibrated concentrations.

Accordingly, this study aimed to examine how ecological permissiveness, IAD-associated terminal-conversion capacity, and host-associated substrate availability influence modeled allocation between the IAA and skatole sides of the branch. We developed a literature-informed, theoretical, and hypothesis-generating framework representing this branch as a finite-pool allocation problem. It integrates an algebraic pH-dependent allocation model, a one-dimensional plug-flow-reactor-inspired spatial model, and scenario analyses of pool size, logistic parameters, pH sensitivity and assumed pH deviations, conversion capacity, substrate availability, spatial pH trajectories, relative residence, benchmark-conversion assumptions, and non-conversion losses. By separating normalized allocation from scale-dependent absolute outputs, the framework identifies influential assumptions within prespecified ranges. It is neither calibrated to reproduce human fecal, luminal, plasma, or tissue concentrations nor intended to predict clinical responses. Rather, it provides a transparent basis for prioritizing experimentally testable drivers and guiding validation in pH-controlled in vitro fermentation systems, stable-isotope-tracing in vivo studies, and stratified CKD clinical cohorts.

## 2. Results

### 2.1. Technical Total-Pool Scale and Sensitivity to Conversion Assumptions

**Figure 1:**
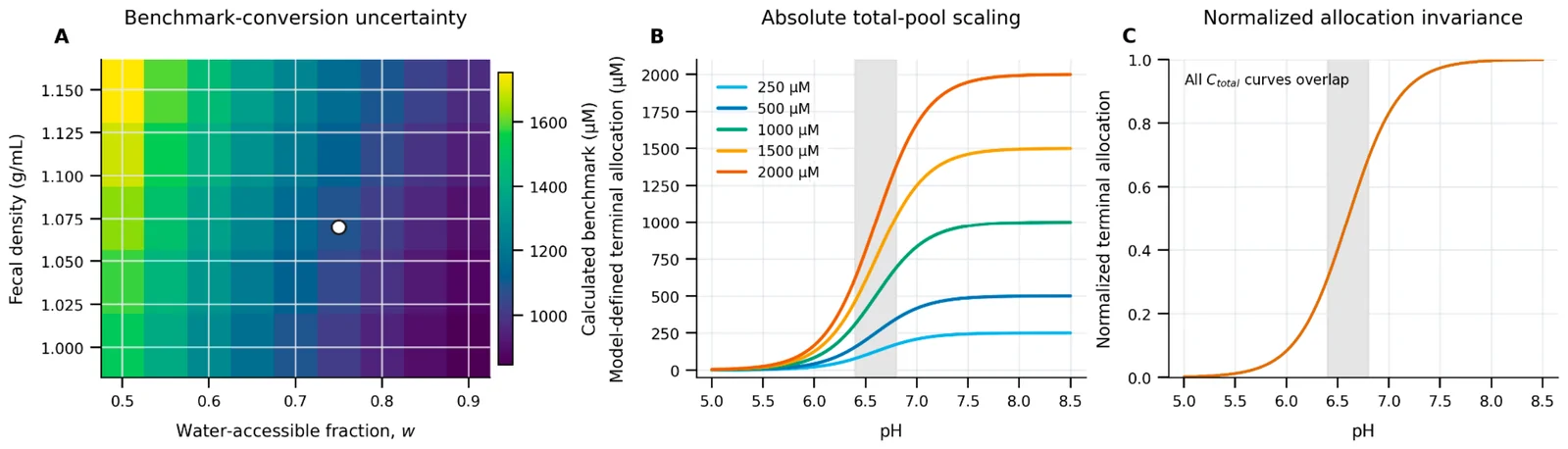
Technical benchmark uncertainty, total-pool scaling, and normalized allocation invariance. (**A**) Sensitivity of the technical benchmark concentration to uncertainty in the water-accessible fraction (w) and fecal density ($\rho_{\mathrm{feces}}$). The heat map shows benchmark concentrations recalculated from a reference fecal skatole burden of $100{\;\mu g\;g}^{-1}\;\mathrm{fresh}\;\mathrm{feces}$ across combinations of w=0.50–0.90 and $\rho_{\mathrm{feces}}=1.00–1.15{\;g\;\mathrm{mL}}^{-1}$. The white circle indicates the reference assumption (w=0.75, $\rho_{\mathrm{feces}}=1.07{\;g\;\mathrm{mL}}^{-1}$) used in subsequent analyses. The resulting benchmark concentrations serve exclusively as technical scaling anchors and do not represent measured luminal indole-3-acetic acid (IAA) concentrations, calibrated fecal or circulating metabolite concentrations, diagnostic thresholds, or recommended exposure levels. The evaluated assumptions altered the absolute technical visualization scale but did not modify the normalized allocation function. (**B**) Absolute distal terminal allocation predicted across total-pool scales of 250, 500, 1000, 1500, and 2000 μM under the reference logistic formulation. Increasing total-pool size proportionally increases absolute distal terminal allocation while preserving the shape of the allocation function. (**C**) Normalized distal terminal allocation generated using total-pool scales of 250–2000 μM. All normalized curves completely overlap, demonstrating invariance of normalized allocation to total-pool scaling. The selected total-pool scale determines absolute output magnitude but does not alter normalized allocation. The shaded region denotes the transition interval surrounding the reference midpoint.

A fecal skatole burden of 100 μg/g fresh feces was converted to a nominal technical total-pool scale using a fecal density of 1.07 g/mL, a skatole molecular weight of $131.2{\;\mu g\;\mu \mathrm{mol}}^{-1}$, and a water-accessible fraction of 0.75. Under these assumptions, the calculated benchmark was 1087.4 μM, equivalent to approximately 1.087 mM. The unrounded computational value was retained in the machine-readable source data, and a rounded value of 1000 μM was used as the reference total-pool scale in subsequent scenario analyses (Table 1; Figure 1A). Across all 45 combinations of water-accessible fraction and fecal density, the recalculated benchmark ranged from 846.9 μM at w=0.90 and $\rho_{\mathrm{feces}}=1.00\;g/\mathrm{mL}$ to 1753.0 μM at w=0.50 and $\rho_{\mathrm{feces}}=1.15\;g/\mathrm{mL}$. The maximum-to-minimum ratio was approximately 2.07. Thus, the selected conversion assumptions changed the absolute technical visualization scale by approximately twofold but did not alter the normalized allocation function. The complete 45-condition matrix is reported in Supplementary Table S1 and Figure S1. These values were used exclusively as technical scaling anchors. They do not represent measured luminal IAA concentrations, calibrated fecal or circulating metabolite concentrations, diagnostic thresholds, or recommended experimental exposure levels. Subsequent analyses therefore evaluated model-defined precursor allocation rather than systemic uremic-toxin burden.

### 2.2. pH-Dependent Algebraic Allocation and Total-Pool Scaling

**Figure 2:**
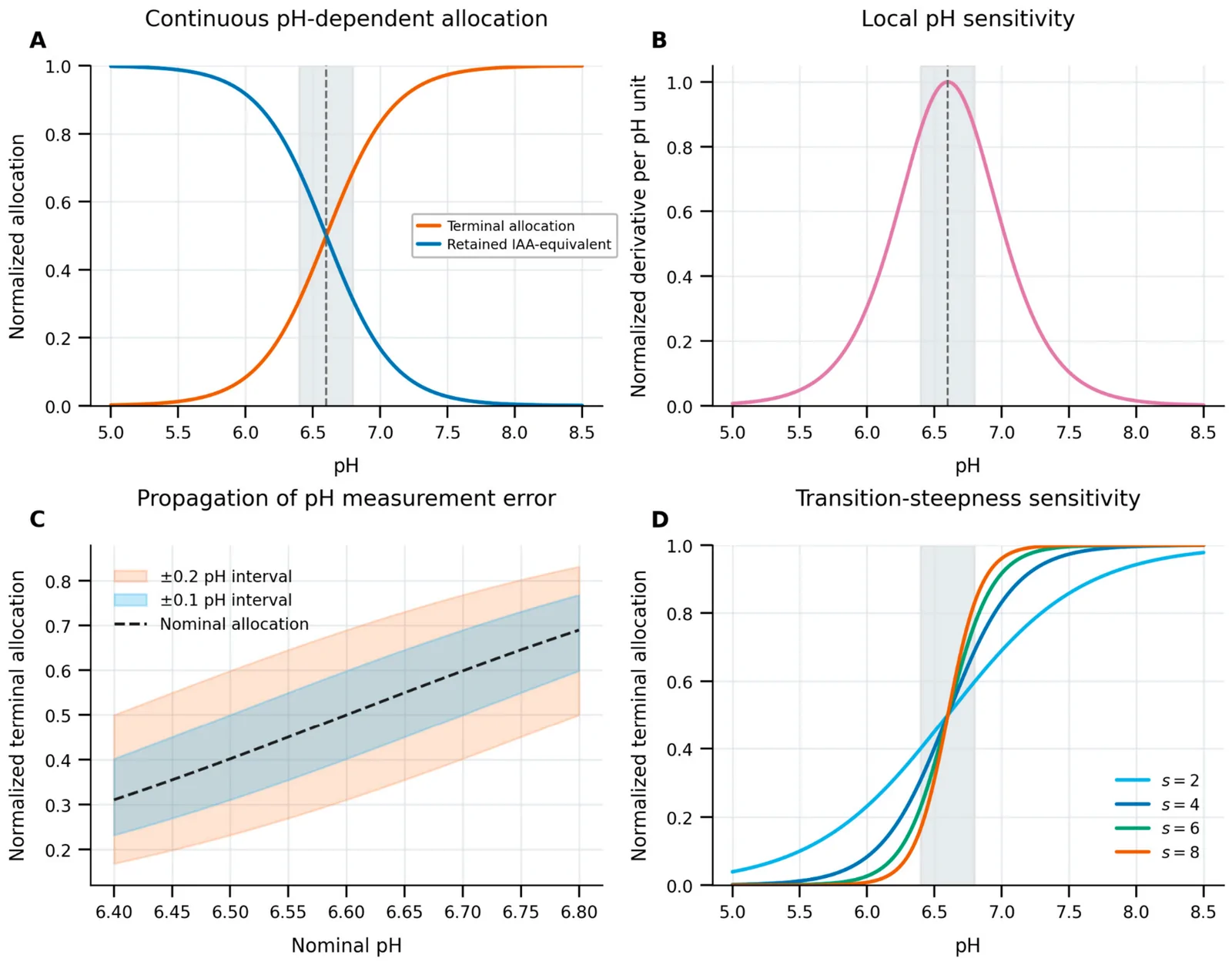
Continuous pH-dependent allocation and sensitivity characteristics. (**A**) Logistic allocation function describing continuous redistribution between retained IAA-equivalent material and distal terminal allocation as a function of pH. Equal allocation occurs at ${\mathrm{pH}}_{\mathrm{mid}}=6.6$, where retained and distal terminal fractions are both 0.5. (**B**) Normalized local sensitivity of the logistic allocation function expressed as the normalized derivative with respect to pH. Maximum normalized sensitivity occurs at $\mathrm{pH}={\mathrm{pH}}_{\mathrm{mid}}$ and decreases toward both tails of the response curve. (**C**) Propagation of assumed pH deviations into predicted distal terminal allocation within the high-gradient region of the reference formulation. Shaded intervals indicate allocation ranges generated by assumed symmetric deviations of ±0.1 and ±0.2 pH units around the nominal pH value. The dashed line represents the nominal allocation. (**D**) Effect of transition steepness (s) on distal terminal-allocation dynamics. Increasing s reduces the 10–90% transition width and increases maximum normalized sensitivity while preserving equal allocation at $\mathrm{pH}={\mathrm{pH}}_{\mathrm{mid}}$. The shaded regions indicate the transition zone surrounding ${\mathrm{pH}}_{\mathrm{mid}}$. The pH 6.4–6.8 region shown here corresponds to the most pH-sensitive interval of the adopted reference formulation and should not be interpreted as an empirically identified or universally applicable biological threshold.

Under the reference logistic parameters ${\mathrm{pH}}_{\mathrm{mid}}=6.6$ and s=4, normalized terminal allocation increased from 0.0832 at pH 6.0 to 0.3100 at pH 6.4, 0.5000 at pH 6.6, 0.6900 at pH 6.8, and 0.9608 at pH 7.4. At $C_{\mathrm{total}}=1000\;\mu M$, these fractions corresponded to terminal allocations of 83.2, 310.0, 500.0, 690.0, and 960.8 μM, respectively. The complementary retained allocation decreased correspondingly, and equal allocation occurred at pH 6.6 (Figure 1B,C and Figure 2A; Table 2). Total-pool scaling was evaluated at $C_{\mathrm{total}}$ values of 250, 500, 1000, 1500, and 2000 μM. At pH 6.6, absolute terminal allocation increased from 125 to 1000 μM across this range, whereas the normalized terminal fraction remained 0.5000. At pH 6.4, absolute terminal allocation increased from 77.5 to 620.1 μM, whereas the normalized fraction remained 0.3100. At pH 6.8, absolute terminal allocation increased from 172.5 to 1379.9 μM, whereas the normalized fraction remained 0.6900. The maximum difference among normalized algebraic curves generated at different total-pool sizes was zero. The same scale invariance was observed in the loss-free spatial model. Under constant pH 6.0, normalized distal terminal allocation remained 0.2830, while absolute $S\left(1\right)$ increased from 70.8 to 566.0 μM as $C_{\mathrm{total}}$ increased from 250 to 2000 μM. Under the linear pH 6.2–7.4 profile, normalized distal terminal allocation remained 0.9217, while absolute $S\left(1\right)$ increased from 230.4 to 1843.3 μM. Thus, $C_{\mathrm{total}}$ determined the absolute output scale without changing normalized allocation. Selected representative values are summarized in Table 2. A formatted summary is provided in Supplementary Table S2, and the complete 1755-row algebraic grid is provided in its machine-readable source-data file.

### 2.3. Local pH Sensitivity, Assumed pH Deviations, and Logistic-Parameter Variation

The local pH derivative reached its maximum at $\mathrm{pH}={\mathrm{pH}}_{\mathrm{mid}}$. Under s=4, the maximum normalized sensitivity was 1.0 per pH unit. At $C_{\mathrm{total}}=1000\;\mu M$, the corresponding maximum absolute sensitivity was 1000 μM per pH unit (Figure 2A,B; Table 3). The complete family of logistic allocation curves is shown in Supplementary Figure S2. At pH 6.6, a symmetric assumed pH deviation of ±0.1 produced normalized terminal allocations of 0.4013 and 0.5987. The full lower-to-upper allocation interval was 0.1974, equivalent to 197.4 μM at the reference pool. An assumed deviation of ±0.2 produced normalized terminal allocations of 0.3100 and 0.6900, giving a full lower-to-upper allocation interval of 0.3799, equivalent to 379.9 μM. Within the evaluated nominal pH range of 6.40–6.80, the propagated intervals were largest near the reference midpoint of pH 6.6 (Figure 2C; Supplementary Table S3B and Figure S3). The 16-condition logistic grid combined ${\mathrm{pH}}_{\mathrm{mid}}$ values of 6.2, 6.4, 6.6, and 6.8 with s values of 2, 4, 6, and 8. The 10–90% transition width decreased from 2.1972 pH units at s=2 to 1.0986 at s=4, 0.7324 at s=6, and 0.5493 at s=8. The corresponding maximum normalized sensitivities were 0.5, 1.0, 1.5, and 2.0 per pH unit, respectively. All 80 documented analytical-identity tests passed. Complete transition-location, transition-width, local-sensitivity, and selected-allocation results are reported in Supplementary Table S3A and Figure S2; the effect of transition steepness at the reference midpoint is shown in Figure 2D. The pH 6.4–6.8 high-gradient region was conditional on the reference values of ${\mathrm{pH}}_{\mathrm{mid}}$ and s. It represents the most pH-sensitive region of the reference formulation and should not be interpreted as an empirically identified or universally applicable biological threshold.

### 2.4. Spatial Redistribution Across Reference and Alternative pH Profiles

**Figure 3:**
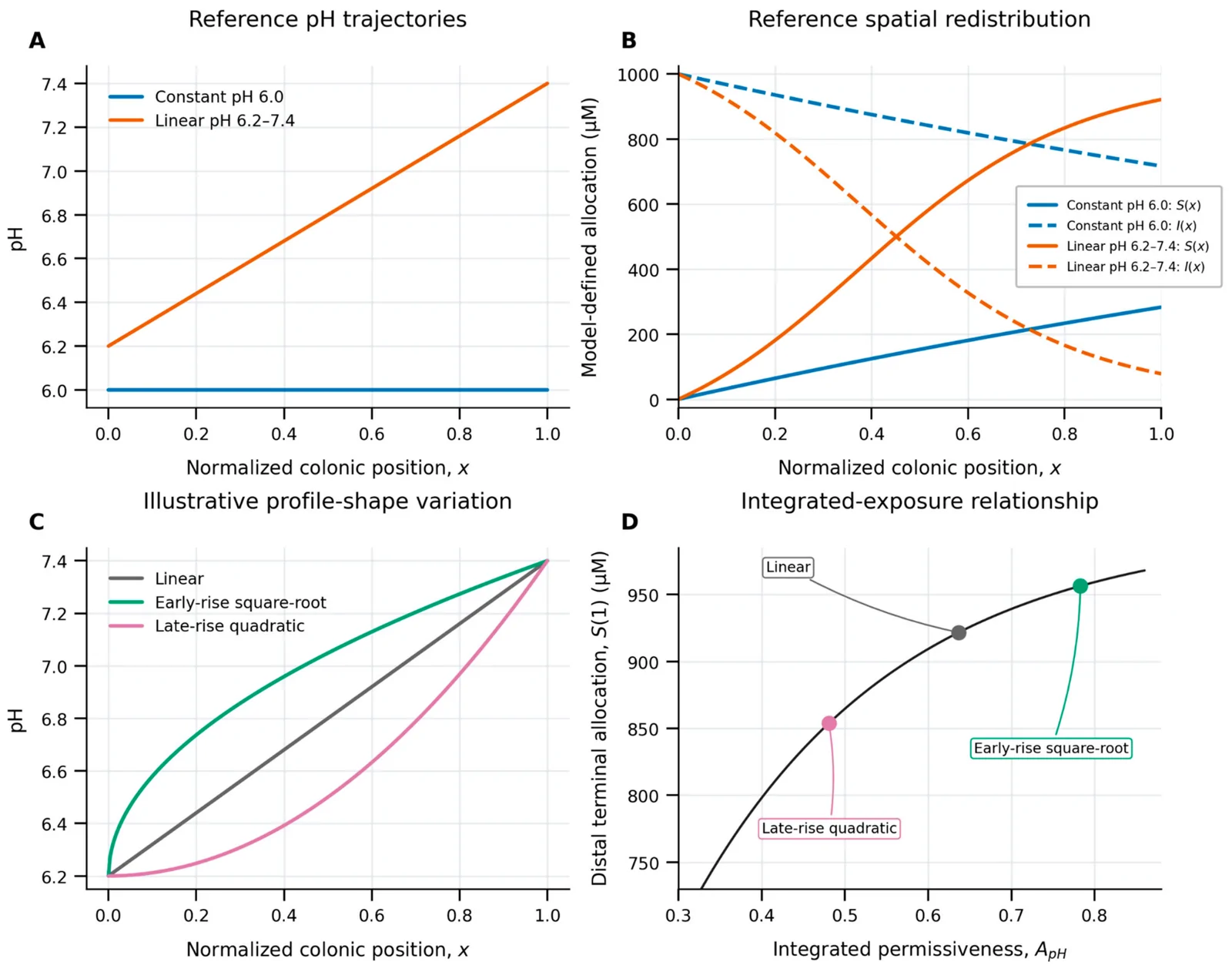
Spatial redistribution, alternative pH trajectories, and integrated permissiveness. (**A**) Reference colonic pH trajectories used in the spatial model. Simulations were performed using a constant pH 6.0 profile and a linear pH 6.2–7.4 profile across the normalized colonic position (x). (**B**) Reference spatial redistribution of retained indole-3-acetic acid (IAA)-equivalent material, $I\left(x\right)$, and distal terminal allocation, $S\left(x\right)$, under the constant pH 6.0 and linear pH 6.2–7.4 reference profiles. (**C**) Representative alternative pH-profile shapes used to evaluate profile-shape robustness. The illustrated trajectories share identical proximal and distal pH boundaries but differ in the spatial distribution of pH values along the modeled colonic axis. (**D**) Relationship between integrated permissiveness ($A_{\mathrm{pH}}$) and distal terminal allocation, $S\left(1\right)$. Distinct pH trajectories generate different exposure integrals and corresponding distal terminal allocations. Profiles shifted to yield identical integrated permissiveness converge to the same distal endpoint within recorded numerical precision. Within the implemented loss-free first-order formulation, distal terminal allocation is determined by integrated modeled permissive exposure rather than by a single local pH value or the proximal-to-distal ordering of an otherwise unchanged pH distribution. The observed convergence reflects dependence of distal terminal allocation on integrated permissive exposure within the specified loss-free first-order formulation. The observed endpoint equivalence represents a structural property of the implemented formulation and should not be interpreted as evidence that spatial ordering is biologically irrelevant in more complex intestinal systems.

Under the reference parameter set, normalized distal terminal allocation was 0.2830 under constant pH 6.0 and 0.9217 under the linear pH 6.2–7.4 profile. At $C_{\mathrm{total}}=1000\;\mu M$ and $L_{3}=1$, these values corresponded to absolute distal terminal allocations of 283.0 and 921.7 μM, respectively (Figure 3A,B; Table 4). Across the documented proximal–distal linear-profile grid, distal terminal allocation ranged from approximately 636.0 μM for the pH 6.0–6.6 profile to 943.2 μM for the pH 6.4–7.4 profile. The reference linear pH 6.2–7.4 profile produced 921.7 μM. Reference constant-profile and linear-reference results are reported in Supplementary Table S4A. The additional linear-profile conditions, including the proximal–distal grid and equal-integrated-permissiveness examples, are reported in Supplementary Figure S4 and its machine-readable source-data file. Among the eight nonlinear forward profiles, integrated permissiveness ranged from 0.4807 for the late-rise quadratic profile to 0.7830 for the early-rise square-root profile. The corresponding distal terminal allocations ranged from 853.8 to 956.4 μM. The linear reference profile produced 921.7 μM. Complete forward, reversed, and exposure-matched profile results are reported in Supplementary Table S4B. After each nonlinear profile was shifted to match the integrated permissiveness of the linear reference, distal terminal allocation converged to the same endpoint within the recorded numerical precision. Reversing a profile while preserving its pH-value distribution and integrated permissiveness did not change the distal endpoint at the recorded precision, although intermediate $I\left(x\right)$ and $S\left(x\right)$ trajectories could differ. Thus, within the specified loss-free first-order formulation, distal terminal allocation depended on integrated modeled permissive exposure rather than on a single local pH value or the proximal-to-distal ordering of an otherwise unchanged pH distribution (Figure 3C,D). This endpoint equivalence was a structural property of the implemented formulation and does not imply that spatial ordering is biologically irrelevant in more complex intestinal systems.

### 2.5. Conversion Capacity, Relative Spatial Progression, and Composite Exposure

**Figure 4:**
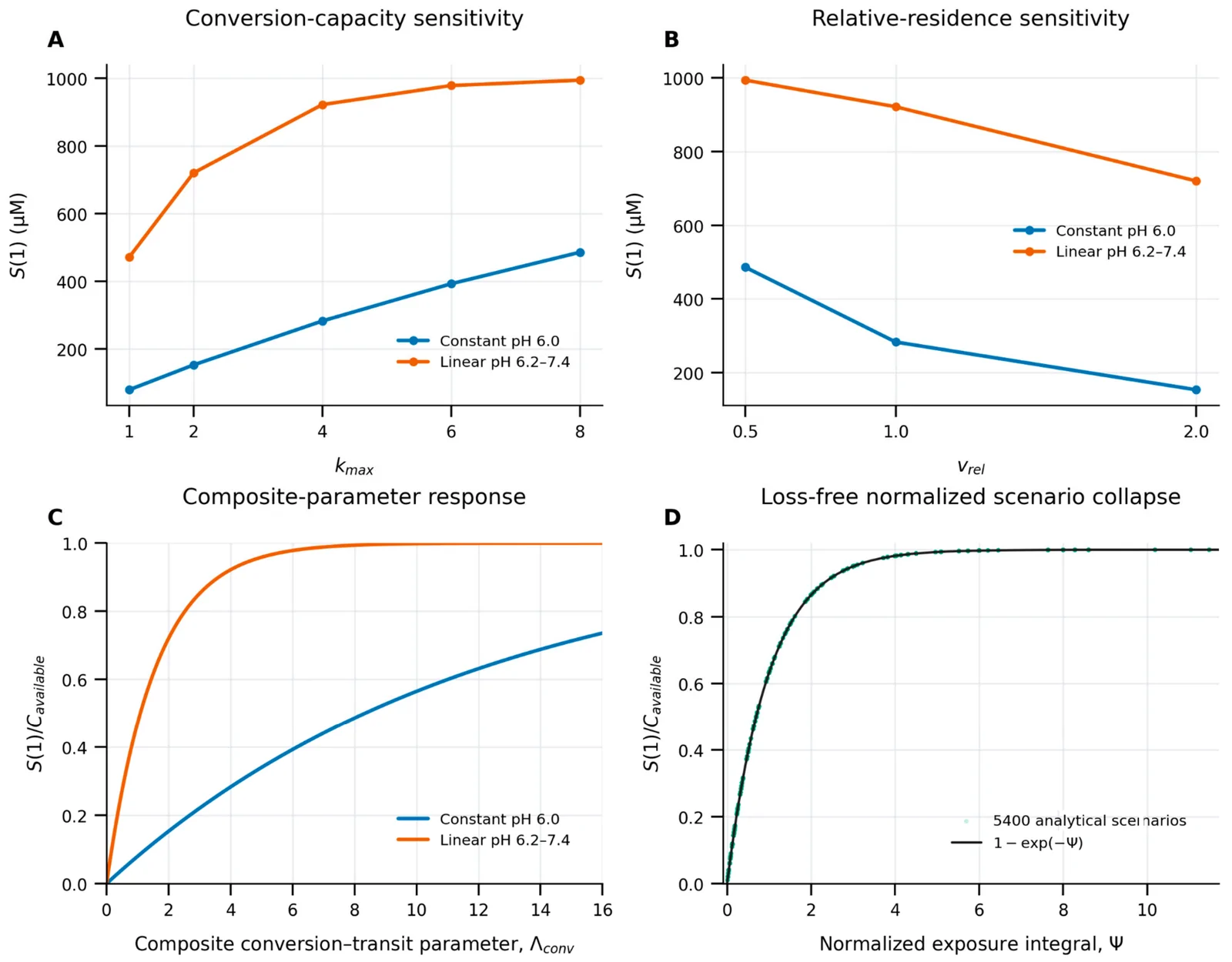
Conversion–transit sensitivity, composite exposure, and normalized scenario collapse. (**A**) Sensitivity of distal terminal allocation, $S\left(1\right)$, to the conversion-capacity parameter $k_{\max}$ under the constant pH 6.0 and linear pH 6.2–7.4 reference profiles. (**B**) Sensitivity of distal terminal allocation, $S\left(1\right)$, to the relative spatial-progression parameter $v_{\mathrm{rel}}$ under the same reference profiles. (**C**) Normalized distal terminal allocation, $S\left(1\right)/C_{\mathrm{available}}$, as a function of the composite conversion–transit parameter $\Lambda_{\mathrm{conv}}$. $\Lambda_{\mathrm{conv}}$ integrates conversion capacity ($k_{\max}$), Terminal Metabolic Lock restriction ($L_{2}$), and relative spatial progression ($v_{\mathrm{rel}}$) within the loss-free formulation. Distinct parameter combinations that generate identical $\Lambda_{\mathrm{conv}}$ values produce identical loss-free trajectories under the same pH profile. (**D**) Collapse of 5400 loss-free analytical scenarios onto the closed-form relationship $\frac{S\left(1\right)}{C_{\mathrm{available}}}=1-\exp\left(-\Psi \right)$ where Ψ denotes the normalized exposure integral. Scenarios spanning 10 pH profiles, five $k_{\max}$ values, four positive $L_{2}$ values, three $v_{\mathrm{rel}}$ values, three $C_{\mathrm{total}}$ values, and three positive $L_{3}$ values converge onto the same normalized response. The overlap demonstrates structural output equivalence for the specified loss-free formulation and shows that normalized allocation is entirely determined by Ψ.

Under constant pH 6.0, distal terminal allocation increased from approximately 79.8 μM at $k_{\max}=1$ to 485.9 μM at $k_{\max}=8$. Under the linear pH 6.2–7.4 profile, distal terminal allocation increased from 471.0 μM at $k_{\max}=1$ to 993.9 μM at $k_{\max}=8$. At $k_{\max}=2,\;4$, and 6, the corresponding linear-profile outputs were approximately 720.1, 921.7, and 978.1 μM, respectively (Figure 4A). Lower relative spatial progression, higher $k_{\max}$, and higher $L_{2}$ increased terminal allocation through the composite parameter $\Lambda_{\mathrm{conv}}$. Relative spatial progression was dimensionless and did not denote calibrated physiological transit time. Parameter combinations with identical $\Lambda_{\mathrm{conv}}$ produced identical loss-free trajectories under the same pH profile. The maximum full-curve difference among the documented equal-$\Lambda_{\mathrm{conv}}$ conditions was zero at the recorded precision (Figure 4B,C; Supplementary Figure S5). Thus, $k_{\max}$, $L_{2}$, and $v_{\mathrm{rel}}$ affected the loss-free endpoint through their composite value $\Lambda_{\mathrm{conv}}$. The resulting output equivalence indicated endpoint-level structural non-identifiability within the reduced loss-free formulation and did not imply biological interchangeability among conversion capacity, Terminal Metabolic Lock restriction, and relative spatial progression.

### 2.6. Separate and Combined Variation in the Three Modeled Locks

**Figure 5:**
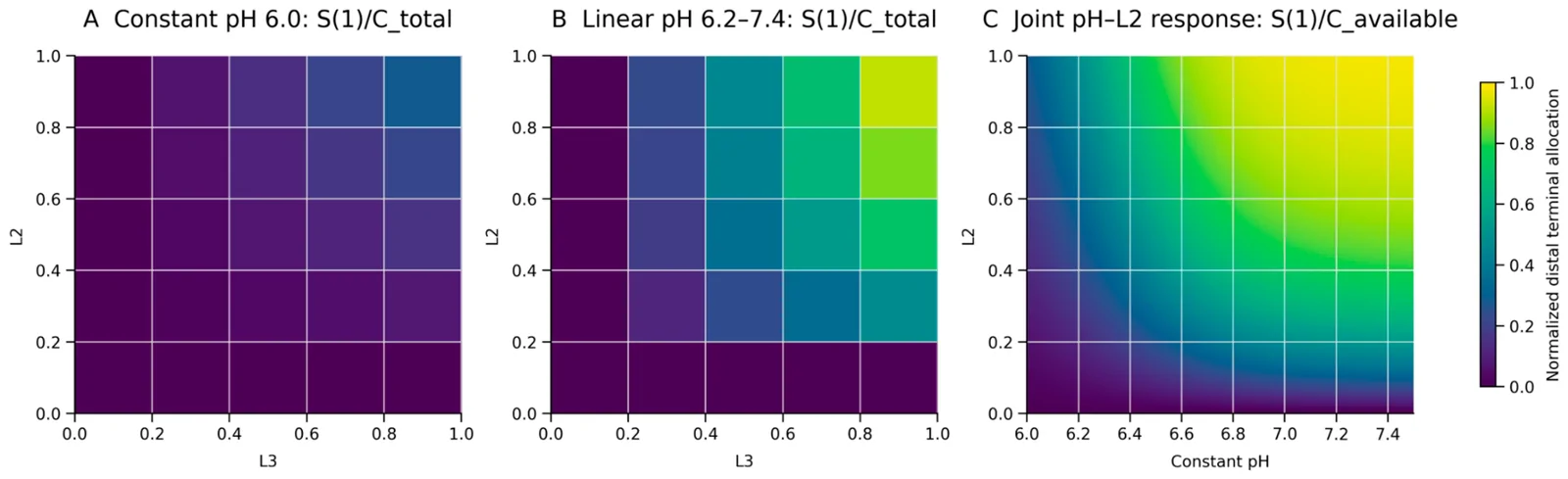
Triple-Lock parameterization of permissive exposure, conversion opportunity, and available-pool scaling. (**A**) Distal terminal allocation normalized to the total pool, $S\left(1\right)/C_{\mathrm{total}}$, across the discrete 5×5 grid of Lock 2 ($L_{2}$) and Lock 3 ($L_{3}$) values under the constant pH 6.0 reference profile. (**B**) Distal terminal allocation normalized to the total pool, $S\left(1\right)/C_{\mathrm{total}}$, across the same $L_{2}-L_{3}$ parameter space under the linear pH 6.2–7.4 reference profile. (**C**) Normalized distal terminal allocation, $S\left(1\right)/C_{\mathrm{available}}$, across continuous combinations of pH and $L_{2}$. The surface spans pH values from 6.0 to 7.5 and $L_{2}$ values from 0 to 1 in 0.01 increments, yielding 15,251 analytical conditions. Across all scenarios, $L_{3}$, representing the Host-Protection Lock and operationally defined as a dimensionless substrate-availability coefficient, scales the dynamically available pool and therefore determines absolute distal terminal allocation, whereas $L_{2}$, representing the Terminal Metabolic Lock, modifies conversion exposure within the available pool. When $L_{2}=0$ or $L_{3}=0$, $S\left(1\right)$ equals zero. For positive $L_{3}$ values, normalization by $C_{\mathrm{available}}$ removes the direct multiplicative effect of $L_{3}$. When $L_{3}=0$, both $C_{\mathrm{available}}$ and $S\left(1\right)$ equal zero, and $S\left(1\right)/C_{\mathrm{available}}$ is mathematically undefined rather than zero. These graphical response surfaces visualize deterministic model outputs across prespecified combinations of ecological permissiveness, terminal-conversion opportunity, and available-pool scaling. They do not establish biological synergy, pharmacological synergy, statistical interaction, effect modification, causal interaction, or experimentally observed co-regulation. Absolute output is jointly determined by the available-pool scale, $L_{3}C_{\mathrm{total}}$, and the normalized conversion term, $1-\exp\left(-\Psi \right)$.

Independent variation of $L_{2}$ and $L_{3}$ generated 25 combinations under each of constant pH 6.0 and linear pH 6.2–7.4, yielding 50 Triple-Lock scenarios (Table 5; Supplementary Table S5). Across both profiles, $L_{3}$ scaled the dynamically available pool and therefore absolute distal terminal allocation, whereas $L_{2}$ altered conversion exposure within the available pool. When either $L_{2}=0$ or $L_{3}=0$, $S\left(1\right)$ was zero. When $L_{3}>0$, normalization by $C_{\mathrm{available}}$ removed the direct multiplicative effect of $L_{3}$. When $L_{3}=0$, both $C_{\mathrm{available}}$ and $S\left(1\right)$ were zero; consequently, $S\left(1\right)/C_{\mathrm{available}}$ was mathematically undefined and was not imputed as zero. Figure 5A and Figure 5B display $S\left(1\right)/C_{\mathrm{total}}$ across the discrete 5×5 $L_{2}\times L_{3}$ grids under constant pH 6.0 and linear pH 6.2–7.4, respectively. Figure 5C displays the normalized pH–$L_{2}$ response, $S\left(1\right)/C_{\mathrm{available}}$, across pH 6.0–7.5 and $L_{2}=0–1$ in 0.01 increments, yielding 15,251 analytical conditions. These surfaces represent deterministic variation in the modeled ecological and terminal-conversion conditions together with available-pool scaling by $L_{3}$. They do not establish biological synergy, pharmacological synergy, statistical interaction, effect modification, or causal interaction. Absolute output was determined jointly by the available-pool scale $L_{3}C_{\mathrm{total}}$ and the normalized conversion term $1-\exp\left(-\Psi \right)$.

### 2.7. Competing Loss and Combined Spatial-Progression–Loss Scenarios

**Figure 6:**
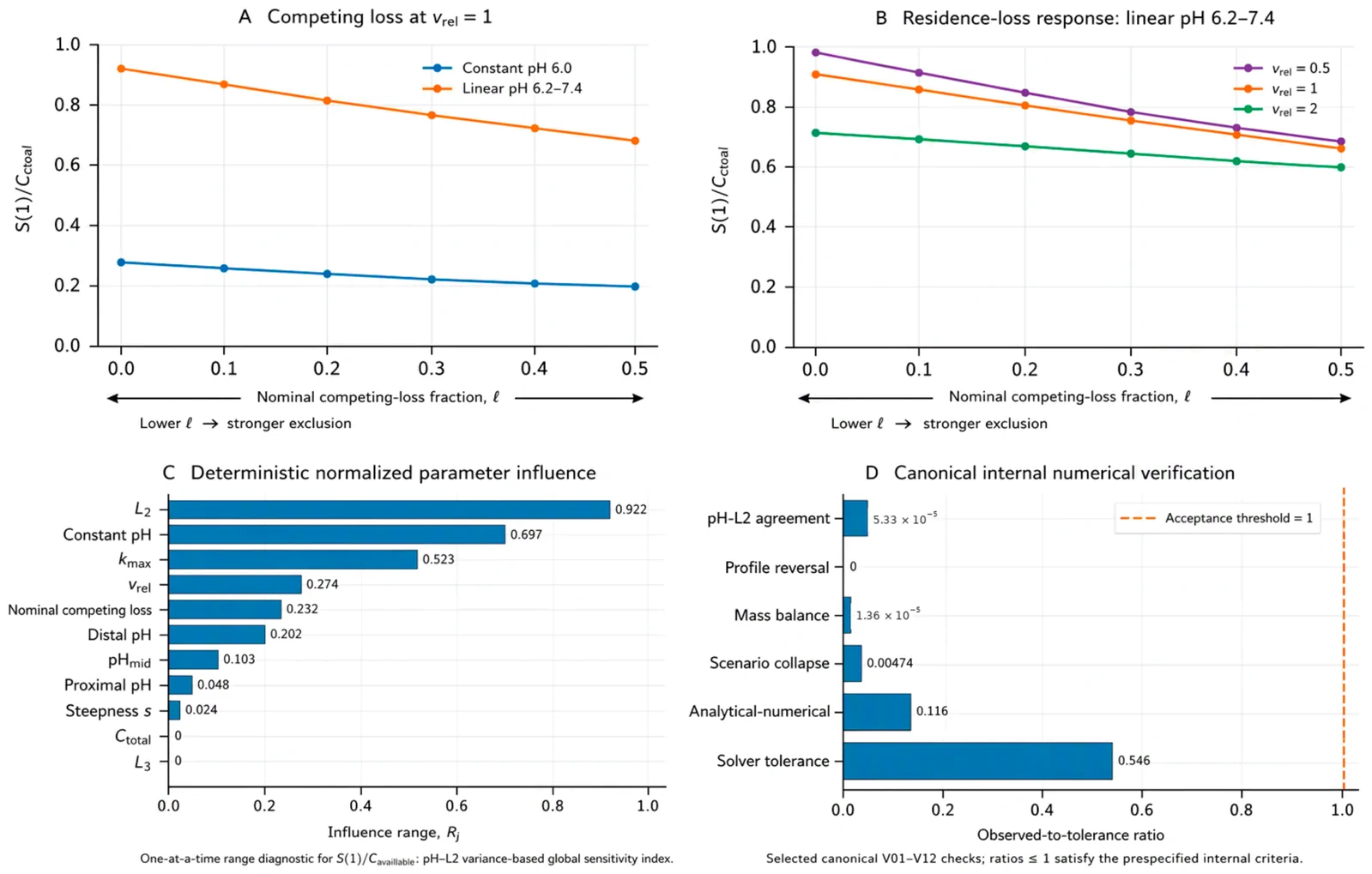
Competing loss, deterministic parameter-range diagnostics, and internal numerical verification. (**A**) Effect of the nominal competing-loss fraction (l) on distal terminal allocation normalized to the original total pool, $S\left(1\right)/C_{\mathrm{total}}$, under the constant pH 6.0 and linear pH 6.2–7.4 reference profiles. Increasing the nominal competing-loss fraction reduces distal terminal allocation under both profiles. Realized loss is not necessarily identical to the nominal competing-loss parameter because conversion and loss compete for the same retained precursor pool. (**B**) Combined effects of relative spatial progression ($v_{\mathrm{rel}}=0.5,\;1,$ and 2) and nominal competing loss (l=0–0.50) on normalized distal terminal allocation under the linear pH 6.2–7.4 reference profile. The largest loss-free outputs occur at $v_{\mathrm{rel}}=0.5$. (**C**) Deterministic one-at-a-time response-range diagnostic for $S\left(1\right)/C_{\mathrm{available}}$ across all 11 prespecified model parameters. Bars represent response ranges ($R_{j}$) measured over the documented parameter intervals. $C_{\mathrm{total}}$ and positive $L_{3}$ values each produced a response range of zero because their direct multiplicative effects cancel analytically when $S\left(1\right)$ is normalized by $C_{\mathrm{available}}=L_{3}C_{\mathrm{total}}$ in the loss-free formulation. These zero ranges are specific to the selected normalized response and do not imply that $C_{\mathrm{total}}$ or $L_{3}$ is irrelevant to absolute distal endpoint allocation, $S\left(1\right)$. The $L_{3}=0$ boundary was excluded because $S\left(1\right)/C_{\mathrm{available}}$ is mathematically undefined when $C_{\mathrm{available}}=0$. Influence ranges are conditional on the documented parameter intervals, selected reference settings, and response definition. This diagnostic is not a variance-based global sensitivity analysis and should not be interpreted as a statistical effect-size hierarchy, causal ranking, parameter-identifiability result, or universal ranking of biological importance. (**D**) Canonical internal numerical verification based on selected manuscript-level checks from the V01–V12 verification framework. Displayed results correspond to V05 (profile-reversal endpoint equivalence), V06 ($\mathrm{pH}–L_{2}$ analytical–numerical agreement), V07 (scenario-collapse summary), V09 (solver-tolerance stability), V10 (analytical–numerical full-curve agreement), and V11 (mass-balance audit). Bars represent observed-to-tolerance ratios, and values ≤1 satisfy the predefined acceptance criterion. Verification results support internal algebraic consistency, solver stability, structural output equivalence, mass conservation, boundary consistency, and state nonnegativity within the documented verification scopes. These verification results do not constitute experimental, biological, physiological, external, or clinical validation. No empirical dataset was used for model fitting, physiological calibration, or evaluation of predictive accuracy.

The combined analysis comprised 24 conditions defined by $v_{\mathrm{rel}}$ values of 0.5, 1, and 2; nominal competing-loss fractions l of 0, 0.10, 0.25, and 0.50; and the two reference pH profiles. Increasing l reduced absolute distal terminal allocation and increased realized loss under both profiles. Because conversion and loss competed for the same retained precursor, realized loss generally differed from the nominal isolated-loss parameter. At the reference relative spatial progression, $v_{\mathrm{rel}}=1$, increasing the nominal competing-loss fraction from 0 to 0.50 reduced $S\left(1\right)/C_{\mathrm{total}}$ from 0.2830 to approximately 0.208 under constant pH 6.0 and from 0.9217 to approximately 0.690 under the linear pH 6.2–7.4 profile (Figure 6A). Under the linear pH 6.2–7.4 profile, the complete relative-progression analysis showed that $S\left(1\right)/C_{\mathrm{total}}$ decreased from approximately 0.994 to 0.665 at $v_{\mathrm{rel}}=0.5$, from 0.922 to 0.690 at $v_{\mathrm{rel}}=1$, and from 0.720 to 0.603 at $v_{\mathrm{rel}}=2$ as l increased from 0 to 0.50 (Figure 6B). Across the complete grid, the largest loss-free outputs occurred at $v_{\mathrm{rel}}=0.5$. Under this condition, absolute $S\left(1\right)$ decreased from 485.9 to 282.6 μM under constant pH 6.0 and from 993.9 to 664.7 μM under the linear pH 6.2–7.4 profile as l increased from 0 to 0.50. The corresponding realized loss fractions at l=0.50 were 0.5888 and 0.3337, respectively. All recorded distal endpoint states were nonnegative, and the sums of the distal compartments were consistent with total-pool accounting within the recorded mass-balance residual for each condition. The maximum mass-balance residual recorded for each condition was below the documented V11 tolerance. Mass-balance results for all 24 combined relative-progression–loss conditions are summarized in Table 6. Complete distal states, normalized compartment fractions, conditional terminal fractions, and maximum mass-balance residuals for all 24 conditions are reported in Supplementary Table S6. The two supplied coordinate-level spatial trajectories are provided separately in the source data for Supplementary Figure S6A,B. Under constant pH 6.0 and $v_{\mathrm{rel}}=0.5$, the conditional terminal fraction among non-lost distal material increased from 0.4859 to 0.6874 as l increased from 0 to 0.50, despite a decrease in absolute $S\left(1\right)$. This increase resulted from the changing denominator and did not indicate increased absolute terminal production. Thus, absolute terminal allocation, terminal allocation normalized to the original available pool, realized loss, and conditional terminal allocation among non-lost distal material represented distinct model outputs and could respond differently to the same parameter change. The conditional terminal fraction did not provide a direct surrogate for absolute terminal production.

### 2.8. Output-Scale Dependence of Deterministic One-at-a-Time Ranges

One-at-a-time range analysis used $S\left(1\right)/C_{\mathrm{available}}$ as the structural response. $L_{2}$ produced the largest deterministic response range over its documented interval (0.9217), followed by constant pH (0.6966), $k_{\max}$ (0.5229), $v_{\mathrm{rel}}$ (0.2738), nominal competing-loss fraction (0.2316), distal pH (0.2016), ${\mathrm{pH}}_{\mathrm{mid}}$ (0.1029), proximal pH (0.0484), and transition steepness (0.0243). $C_{\mathrm{total}}$ and positive $L_{3}$ values each produced a response range of zero because their direct multiplicative effects cancel analytically when $S\left(1\right)$ is normalized by $C_{\mathrm{available}}=L_{3}C_{\mathrm{total}}$ in the loss-free formulation. These zero ranges are specific to the selected normalized endpoint and should not be interpreted as evidence that $C_{\mathrm{total}}$ or $L_{3}$ is unimportant for absolute distal endpoint allocation, $S\left(1\right)$ (Figure 6C; Supplementary Table S7). The $L_{3}=0$ boundary was excluded because the normalized response was undefined. The displayed ordering was conditional on the documented parameter intervals, reference settings, and response definition. Figure 6C therefore represents a deterministic one-at-a-time response-range diagnostic over the evaluated parameter intervals and not a variance-based global sensitivity analysis. The ordering is not a statistical effect-size hierarchy, causal ranking, parameter-identifiability result, or universal ranking of biological importance. In particular, the observed range for $L_{2}$ indicates only that $L_{2}$ produced the largest deterministic response range for the selected normalized output over the evaluated interval.

### 2.9. Loss-Free Scenarios Collapsed onto a Normalized Exposure Curve

Consistent with the analytical relationship, 5400 loss-free scenarios collapsed onto a single normalized relationship across 10 pH profiles, five kmax values, four positive L2 values, three vrel values, three Ctotal values, and three positive L3 values (Figure 4D). The collapse showed that normalized loss-free allocation was determined by Ψ, whereas Ctotal and positive L3 values canceled after normalization by Cavailable. Across the complete 5400-scenario analytical grid, the normalized model output and the exact prediction agreed at the recorded precision. V07 and V08 report the fixed aggregate verification results for 600 endpoint ODE runs in the scenario-collapse analysis. During revision, the complete analytical scenario-collapse grid and selected supporting calculations were generated from the documented equations, parameter settings, and analysis conditions to improve procedural transparency. These supporting calculations confirm the exact closed-form relationship and do not replace, revise, or redefine the fixed V07 and V08 aggregate verification values. The scenario-collapse observed-to-tolerance ratio displayed in Figure 6D is based on the fixed V07 verification-summary value. Thus, equal Ψ values generated the same normalized allocation at the recorded precision of the specified loss-free formulation.

### 2.10. Numerical Stability and Internal Verification

All 12 documented internal-verification checks were classified as PASS in the fixed authoritative verification summary. For V06, the summary reported a maximum analytical–numerical difference below the predefined tolerance across the documented 70 pH–L2 conditions. Solver-tolerance stability across 24 runs satisfied the documented V09 criterion. The V10 summary reported a maximum full-curve analytical–numerical difference below the predefined tolerance. During revision, selected supporting records were newly generated or reconstructed from the documented equations, parameter settings, and analysis conditions to improve procedural transparency. These records include current supporting calculations for V03–V06, the 24-run solver-tolerance assessment for V09, and a 1505-location coordinate-level procedural-support record for V10. The V10 file identified with the suffix “_RECONSTRUCTED” is revision-stage procedural support and does not replace, revise, or redefine the fixed aggregate V10 value. The current 24-condition progression–loss dataset provides a separate condition-specific mass-balance assessment. This dataset is distinct in analytical scope from the fixed aggregate V11 verification summary, and the two records therefore serve different verification purposes. The condition-specific dataset does not replace, revise, or redefine the V11 value. Observed-to-tolerance ratios for the six checks displayed in Figure 6D were below the normalized acceptance threshold of 1. Figure 6D presents selected verification values for V05, V06, V07, V09, V10, and V11. Selected verification results for V07–V12 are reported in Table 7, and the complete V01–V12 scopes, criteria, tolerances, observed values, units, observed-to-tolerance ratios, statuses, and concise interpretations are reported in Supplementary Table S8. Detailed record classifications, supporting-file locations, and manuscript mappings are provided in the verification inventory. Within the documented scopes, the verification results support the internal mathematical and numerical consistency of the implemented framework. They do not constitute experimental, biological, physiological, external, or clinical validation.

## 3. Discussion

### 3.1. Principal Findings and Structural Interpretation

The model’s central contribution is not the prediction of a universal pH threshold or a clinical outcome, but the demonstration that intestinal indole-3-acetic acid (IAA)-to-skatole allocation can be decomposed into an available-pool scale and an integrated conversion exposure, with direct consequences for endpoint interpretation, mechanistic identifiability, and prospective experimental design. The available-pool scale is

$$C_{\mathrm{available}}=L_{3}C_{\mathrm{total}}$$

and the loss-free structural response is

$$S\left(1\right)=C_{\mathrm{available}}\left\{1-\exp\left(-\Psi \right)\right\}$$

Here, $C_{\mathrm{available}}$ defines the available-pool scale, Ψ is a dimensionless integrated conversion exposure, and $S\left(1\right)/C_{\mathrm{available}}$ represents conversion within the dynamically available pool. Ecological permissiveness denotes the model-assigned pH-dependent modulation of terminal conversion rather than a directly measured property of a specific organism or enzyme. The findings can be organized hierarchically:

- Primary Conceptual Contribution: The separation of available-pool scale from conversion exposure.
- Primary Mathematical Result: The analytically derived collapse of the loss-free normalized endpoint onto Ψ, evaluated across the complete 5400-scenario analytical grid.
- Primary Interpretive Consequence: A common distal endpoint cannot uniquely identify the underlying biological cause.
- Primary Empirical Implication: IAA, skatole, pH, IAD-associated function, transit-related variables, and loss-related outputs must be measured together if competing mechanisms are to be distinguished.

This structural compression is both informative and restrictive. Total-pool size and $L_{3}$ determine the scale available for conversion, whereas pH-dependent permissiveness, $k_{\max}$, $L_{2}$, and $v_{\mathrm{rel}}$ determine normalized loss-free allocation through Ψ. Competing loss then separates absolute terminal allocation, total-pool-normalized compartment allocation, realized loss, and the conditional terminal fraction among non-lost distal material into outputs that may respond differently to the same parameter change. The biological basis for representing a terminal IAA-to-skatole branch is supported by evidence identifying IAA as a direct precursor and indoleacetate decarboxylase (IAD) as a glycyl-radical enzyme responsible for skatole formation [36,40,41,42,43]. Experimental studies further indicate that IAA and skatole can produce distinct cellular responses involving inflammatory signaling, proliferation, xenobiotic-receptor activation, and proteostatic regulation [26,27,28,29,30,31,32,38,39]. These observations motivate branch-specific investigation but do not imply that the model-defined pools directly quantify cellular signaling activity, toxicity, or tissue exposure. Diet, environmental pH, microbial-community composition, and pathway-specific microbial enzymes can alter tryptophan metabolism and the generation of gut-derived metabolites [4,5,8,42,47,48,49,50,69,70]. The present framework organizes selected determinants into a reduced structure that identifies which mechanisms can be distinguished from a distal endpoint and which remain confounded. Its relevance to chronic kidney disease (CKD) is therefore upstream and hypothesis-generating: it addresses an intestinal microbial-metabolite allocation process rather than systemic uremic-solute kinetics. The framework satisfied the documented internal criteria reported in Section 5.23 and Supplementary Table S8, but it is not a calibrated predictor of circulating solute burden, treatment response, or patient outcomes.

### 3.2. Conditional pH Sensitivity and Ecological Interpretation

The reference formulation placed the equal-allocation point at pH 6.6, where local sensitivity was maximal. The logistic midpoint determines the location of the high-gradient region, whereas the steepness parameter determines its width and local slope. Accordingly, pH 6.6 is a reference equal-allocation point generated by the selected parameterization, not a universal physiological threshold. Terms such as bifurcation, metabolic inversion, or switch would imply a discontinuity or an experimentally established state transition that is absent from the smooth, monotonic logistic function. Under the reference parameters, the evaluated interval surrounding the midpoint (pH 6.4–6.8) included the maximum-sensitivity point at pH 6.6 and the adjacent high-gradient portion of the reference response. Altering either logistic parameter shifted or changed the width of this region. The assumed-pH-deviation analysis illustrates why measurements near this region require care:

- At the reference midpoint, assumed deviations of ±0.1 and ±0.2 pH units produced full normalized allocation intervals of approximately 0.20 and 0.38, respectively.
- These intervals are deterministic consequences of the assumed perturbations and are not uncertainty estimates for a particular instrument, specimen-processing protocol, or clinical sampling procedure.

Experiments should therefore document the measurement technology, calibration procedure, sample matrix, sampling location, collection-to-measurement interval, temperature, atmospheric exposure, and technical repeatability. A single fecal pH measurement is not an exact surrogate for conditions throughout the colon; intestinal and fecal pH vary by anatomical location, physiological state, and measurement context [64,65]. The biological rationale for pH is ecological rather than enzyme-specific. Fermentable-carbohydrate availability and short-chain fatty acid production can alter colonic fermentation and acid-base conditions [53,54,55,56,63,64,65], while dietary composition and pH can redirect microbial tryptophan metabolism [47,48,49,50]. Community structure and environmental adaptation may further modify the response [52,68]. Testing the model-derived directionality will require pH manipulation as independently as practicable from substrate composition, because substrate changes may simultaneously alter pH, biomass, pathway expression, precursor availability, and cross-feeding. Empirical estimation of the midpoint and steepness will require repeated, pH-controlled measurements of IAA and skatole formation over a range prespecified in the future validation protocol. Until then, the logistic parameters remain transparent scenario assumptions rather than biological constants.

### 3.3. Structural Compression Within the Triple-Lock Framework

The Lock terminology denotes the conceptual location at which restriction is imposed in the framework; it does not imply that each Lock corresponds to a single measurable biological mechanism:

- Upstream Ecological Lock: Represents local pH-dependent permissiveness.
- Terminal Metabolic Lock ($L_{2}$): Modifies terminal-conversion capacity.
- Host-Protection Lock ($L_{3}$): Scales the model-defined precursor pool available to enter the luminal system.

The $L_{2}\times L_{3}$ analysis showed that these coefficients are not interchangeable:

- $L_{2}$ altered conversion within the available pool, whereas $L_{3}$ scaled the available pool itself.
- When $L_{2}=0$, terminal allocation was zero despite a positive available pool.
- When $L_{3}=0$, the available pool and all dynamic states were zero, and $S\left(1\right)/C_{\mathrm{available}}$ was undefined rather than a zero normalized response.

$L_{2}$ and $L_{3}$ are reduced coefficients, not direct measurements of IAD abundance, catalytic activity, epithelial integrity, permeability, or barrier function. Their values cannot be assigned to a patient, microbial community, or experimental system without independent mapping. Similarly, $v_{\mathrm{rel}}$ is not a calibrated measure of intestinal transit time; transit-related measurements are proposed only as candidate empirical variables for future mapping. A second compression occurred among $k_{\max}$, $L_{2}$, and $v_{\mathrm{rel}}$. In the documented comparison, $C_{\mathrm{available}}$ and the pH profile were held constant, and equal $\Lambda_{\mathrm{conv}}$ values generated the same absolute loss-free $I\left(x\right)$ and $S\left(x\right)$ trajectories at all 301 recorded coordinates. These inputs nevertheless represent distinct concepts, and distal allocation alone cannot separate their contributions. Together with the available-pool compression, this result shows why a common endpoint cannot identify a unique mechanism. Discriminating among alternatives will require independent measurements of precursor availability, pH, transit-related variables, and IAD-associated function. The nonlinear $\mathrm{pH}–L_{2}$ surface should not be interpreted as biological synergy; interaction would require a factorial experiment with independent manipulation, replication defined in the future protocol, and a prespecified statistical interaction analysis.

### 3.4. Integrated Spatial Exposure, Competing Loss, and Output Definition

Distal loss-free allocation was governed by cumulative modeled permissive exposure rather than by a single local pH value. Profiles with different integrated permissiveness produced different endpoints, whereas exposure-matched profiles converged to the same distal endpoint. Reversing spatial order did not change that endpoint at the recorded precision, although intermediate trajectories could differ. These invariances are properties of the first-order scalar formulation and may not persist with microbial growth, substrate depletion, nonlinear kinetics, feedback, spatially varying loss, or time-varying pH. Introducing competing non-conversion loss reduced the absolute terminal amount because conversion and loss drew from the same retained precursor pool. The realized total-pool-normalized loss, $Q_{\mathrm{loss}}\left(1\right)/C_{\mathrm{total}}$, did not generally equal the nominal isolated-loss fraction l, because conversion and loss acted concurrently on the same precursor pool. Interpretation therefore requires explicit specification of the denominator. The principal outputs were

1. Absolute terminal amount: $S\left(1\right)$;
2. Terminal allocation normalized to total pool: $S\left(1\right)/C_{\mathrm{total}}$;
3. Realized loss normalized to total pool: $Q_{\mathrm{loss}}\left(1\right)/C_{\mathrm{total}}$;
4. Conditional terminal fraction among non-lost distal material: $S\left(1\right)/\left[I\left(1\right)+S\left(1\right)\right]$.

For loss-free structural analyses, $S\left(1\right)/C_{\mathrm{available}}$ isolated conversion within the dynamically available pool. In some scenarios, the absolute terminal amount decreased while the conditional terminal fraction increased. This did not indicate an increase in the absolute model-defined terminal amount; it resulted from depletion of the denominator. Reporting a conditional fraction without the corresponding absolute or normalized output could therefore be misleading. The one-at-a-time analysis reinforced this denominator dependence: $C_{\mathrm{total}}$ and positive $L_{3}$ values changed absolute output but canceled from $S\left(1\right)/C_{\mathrm{available}}$, whereas parameters controlling conversion exposure altered allocation within the available pool.

### 3.5. Potential Relevance to Upstream Investigation of Gut-Derived Uremic Solutes

Conventional renal replacement therapies incompletely control several protein-bound and gut-derived uremic solutes [8,35], and their pathological accumulation provides a rationale for complementary source-directed approaches [10,35]. Experimental inhibition or genetic manipulation of microbial pathways has shown that intestinal source control can alter circulating gut-derived solutes [69,70], although those studies examined pathways distinct from the modeled IAA-to-skatole branch. The present decomposition identifies two experimentally testable classes of perturbation:

1. Those that alter the available precursor pool;
2. Those that alter conversion within that pool.

Skatole alone would not reveal whether a change arose from precursor supply, pH, IAD-associated function, transit-related conditions, or competing loss. A mechanistically informative study should therefore measure IAA, skatole, pH, pathway function, transit-related variables, and relevant alternative or loss-associated outcomes concurrently. CKD cohorts are heterogeneous in renal and treatment factors, intestinal and transit-related factors, and dietary, medication, and specimen-related factors [1,4,5,6]. These categories should be captured prospectively because they can alter substrate delivery, fermentation, fecal water content, and interpretation of microbial-metabolite measurements. Detailed operational variables are summarized in Table 8. Fermentable carbohydrates can alter colonic fermentation and short-chain fatty acid production [53,54,55,56,57,58,63], and dietary fiber can modify microbial tryptophan metabolism and indolic-metabolite profiles [47,49,59,60,61,62]. However, the direction and magnitude of these effects depend on substrate composition, host context, and community structure. For CKD-oriented experimental development, low-potassium, low-phosphorus resistant-starch formulations may be prioritized as candidate fermentation substrates rather than making generalized recommendations for high-fiber intake. This prioritization is a prospective experimental strategy, not a clinical recommendation; composition, dose, tolerability, gastrointestinal effects, and nutritional consequences require direct evaluation in dialysis and non-dialysis populations and in participants stratified by constipation and intestinal inflammation. The model itself does not estimate clinical efficacy or safety. Although the 1000 μM reference does not represent a measured or predicted luminal IAA concentration, this sensitivity-tested technical scale may be considered as one reference point when defining exploratory concentration ranges for future in vitro studies. It does not, by itself, provide biological or physiological justification for selecting 1000 μM as an experimental exposure. Any future concentration-response study should determine its tested range independently for the specific biological system, include lower concentrations, and incorporate appropriate controls for cell viability and nonspecific stress.

### 3.6. Empirical Validation Strategy

The model generates five falsifiable hypotheses across three sequential validation levels:

**H1.**  *pH directionality.*

**H2.**  *IAD-associated capacity.*

**H3.**  *Tracer-defined microbial flux.*

**H4.**  *Host-dependent precursor delivery.*

**H5.**  *Clinical pathway coherence.*

Table 8 and Section 5.30 distinguish each hypothesis from its measurements, evidentiary requirements, and progression criteria.

#### 3.6.1. Level 1: pH-Controlled In Vitro Fermentation

**H1.**  *pH directionality: Under the reference directional assumption encoded in the model, lowering pH under otherwise comparable conditions is predicted to reduce the fraction of the initially available IAA pool converted to skatole. The molar conversion fraction should be corrected for baseline skatole and interpreted with residual IAA, alternative metabolites, and total analyte recovery. The pH range, replication, and decision thresholds should be prespecified in the future validation protocol.*

**H2.**  *IAD-associated capacity: Reducing IAD-associated function is predicted to decrease skatole formation at a given pH. Independent or factorial manipulation of pH and IAD-associated function would test the modeled directions and, if intended, biological interaction. Gene abundance, transcript abundance, pathway-positive organism abundance, IAD protein abundance where technically feasible, functional response to IAD-targeted perturbation, and tracer-defined flux should be treated as complementary rather than interchangeable measures.*

Core measurements should include time-resolved pH, initial and residual IAA, baseline-corrected skatole formation, total analyte recovery, microbial-community composition, IAD-associated measures, biomass or growth, viability, and relevant alternative metabolites. These measurements are required to distinguish redirected metabolism from reduced total biomass, selective suppression of pathway-positive organisms, cell death, altered IAA uptake, degradation, adsorption, or analytical loss. Progression beyond Level 1 should require directionality reproduced across independent experiments, recovery and analytical-performance thresholds prespecified in the validation protocol, and evidence that the skatole response cannot be explained solely by loss of total microbial biomass, generalized loss of viability, or inadequate analyte recovery.

#### 3.6.2. Level 2: Stable-Isotope-Tracing In Vivo Studies

**H3.**  *Tracer-defined microbial flux: Labeled precursor tracing should determine whether manipulation of fermentable substrate, pH, transit-related conditions, or IAD-associated microbiota changes flux toward skatole. Labeled IAA would interrogate the terminal branch, whereas labeled tryptophan would interrogate the broader microbial and host network and require isotopologue resolution of competing products. Tracer dose should be sufficiently low to minimize perturbation of endogenous pool size and pathway kinetics while remaining analytically quantifiable. Delivery to the relevant intestinal compartment, absorption or metabolism before arrival, and possible effects on pH or osmolarity should be characterized.*

**H4.**  *host-dependent precursor delivery: Host conditions affecting epithelial function or endogenous substrate availability are predicted to alter labeled precursor delivery to the intestinal branch. Required measurements include isotopologue-resolved IAA and skatole in intestinal contents or feces, labeled precursor recovery, pH, transit-related measures, IAD-associated measures, and relevant epithelial-function readouts.*

Progression beyond Level 2 should require evidence that concentration changes reflect altered flux and that candidate microbial and host variables map reproducibly onto the processes approximated by $L_{2}$ and $L_{3}$.

#### 3.6.3. Level 3: Stratified CKD Clinical Cohorts

**H5.**  *Clinical pathway coherence: Fecal pH and fecal IAA/skatole measurements are hypothesized to associate with IAD-associated measures and selected circulating gut-derived solutes after accounting for renal and treatment factors, intestinal and transit-related factors, and dietary, medication, and specimen-related factors. The primary pathway-oriented variables should be fecal IAA, skatole, pH, and IAD-associated measures; circulating IAA may be evaluated as a secondary systemic analyte.*

Indoxyl sulfate (IS) may be evaluated as an exploratory measure of a related but distinct microbial–host pathway involving microbial indole formation, hepatic metabolism, plasma protein binding, and kidney-dependent accumulation [16,17,18,35,69,70]. Its association with fecal IAA/skatole measurements would not directly validate the modeled branch. Dialysis and non-dialysis strata and major intestinal subgroups should be prespecified, with detailed variables and analytical procedures defined in Table 8 and the future protocol. Clinical investigation should follow evidence of directionality in fermentation systems and flux in isotope-tracing studies. The objective should be analytical reproducibility, pathway coherence, and subgroup dependence rather than causal inference from cross-sectional associations.

#### 3.6.4. Candidate Fecal IAA/Skatole Ratio

The fecal IAA/skatole ratio may serve as a candidate stool-based research measure relating precursor-side and terminal-side signals within the same specimen, but it is not numerically equivalent to the model-defined retained-to-terminal ratio. Any reduction in common dilution variability does not remove bias from differential analyte stability, absorption, post-defecation metabolism, matrix effects, analyte-specific recovery, fecal water content, transit, or values below quantification limits. The ratio should therefore be evaluated alongside the individual concentrations, repeated sampling, fecal water content and pH, validated recovery, internal standards, quality controls, and handling rules prespecified in the analytical-validation protocol. It should not be treated as a stable individual characteristic or clinically actionable biomarker without dedicated validation.

### 3.7. Limitations

Six categories of limitation define the interpretation and applicability of the framework:

1. Parameterization and Biological Mapping: The deterministic ranges were not estimated from biological data, and the logistic midpoint and steepness were not fitted to community-level human conversion data. $L_{2}$ and $L_{3}$ are reduced coefficients that cannot be assigned to patients, communities, or experimental systems without independent mapping.
2. Chemical-Pool and Scaling Assumptions: The technical benchmark was derived from fecal skatole under explicit assumptions, not from a measured luminal IAA pool. The 1000 μM reference is a scaling convention rather than a physiological cutoff or expected exposure; suitability of any experimental concentration must be determined independently.
3. Omission of Biological Processes: The framework does not represent individual taxa, growth, gene regulation, cross-feeding, substrate competition, dynamic replenishment, calibrated anatomy, mixing, intermediate reactions, localized absorption, systemic distribution, protein binding, renal clearance, or dialysis removal. The competing-loss state aggregates removal processes and cannot identify their individual contributions.
4. Formulation-Specific Mathematical Properties: Equal-$\Lambda_{\mathrm{conv}}$ trajectories, spatial-order invariance, and collapse onto Ψ may not persist under nonlinear or saturable kinetics, microbial growth, feedback, heterogeneous progression, spatially varying loss, time-dependent pH, or intermediate-state dynamics.
5. Verification vs. Biological Validation: Internal algebraic and numerical checks establish consistency with the documented equations but not biological or human physiological validity. V07 and V08 report fixed aggregate verification results for the endpoint ODE scenario-collapse analysis. During revision, the complete 5400-scenario analytical grid and selected supporting calculations were generated from the documented equations, parameter settings, and analysis conditions to improve procedural transparency. These supporting calculations provide complementary inspection of the exact closed-form relationship and do not replace, revise, or redefine the fixed V07 and V08 aggregate verification values. More broadly, the supporting and reconstructed records supplied with the article differ in analytical scope and purpose from the fixed V01–V12 aggregate verification summaries. Their role, scope, and manuscript mappings are documented in the verification inventory. Internal verification does not constitute experimental, biological, physiological, external, or clinical validation.
6. Clinical and Methodological Boundaries: The framework does not predict biological concentrations, systemic solute burden, clinical outcomes, or intervention response. The fecal IAA/skatole ratio is unvalidated. The biological context was developed through the targeted narrative literature search described in Section 5.2 rather than a systematic review; no formal risk-of-bias assessment, certainty grading, or quantitative synthesis was performed.

### 3.8. Overall Interpretation

The framework’s conceptual contribution is the separation of available-pool scale from integrated conversion exposure, together with the identification of endpoint measures that cannot distinguish among multiple underlying mechanisms. Its mathematical contribution is the analytically derived collapse of the loss-free normalized endpoint onto Ψ, evaluated across the complete 5400-scenario analytical grid. Its empirical implication is a staged program of pH-controlled fermentation, IAD-focused perturbation, pathway-proximal isotope tracing, broader isotope-resolved network analysis, and carefully stratified CKD cohorts. These studies will determine whether the reduced parameters map to measurable microbial and host processes relevant to the gut–kidney axis. Until such validation is completed, the framework should be regarded as internally verified but not biologically validated, and as hypothesis-generating rather than predictive.

## 4. Conclusions

This study presents a transparent, hypothesis-generating mathematical framework that is sufficiently documented to support independent reimplementation of the intestinal indole-3-acetic acid (IAA)-to-skatole axis as a finite-pool branch-allocation problem. Its central conceptual contribution is the separation of the model-defined available-pool scale from integrated conversion exposure. The analytically derived collapse of the loss-free normalized endpoint onto the dimensionless exposure variable Ψ, evaluated across the complete 5400-scenario analytical grid, shows that distinct combinations of pH-dependent ecological permissiveness, terminal-conversion capacity, and relative spatial progression can produce the same distal normalized response. A common distal endpoint therefore cannot, by itself, identify a unique underlying biological mechanism. The analysis also clarifies that absolute terminal allocation, available-pool-normalized allocation, total-pool-normalized compartment fractions, realized competing loss, and the conditional terminal fraction among non-lost distal material are distinct outputs. Introducing competing non-conversion loss demonstrated that these quantities can change in different directions because they use different denominators. The 1000 μM reference condition served only as a technical scaling benchmark derived under explicit assumptions; it is not a measured or typical human luminal IAA concentration, a physiological threshold, a therapeutic exposure, or a recommended experimental concentration. Variation in the conversion assumptions changed the absolute scale, whereas the principal normalized relationships remained invariant across the evaluated total-pool range.

## 5. Materials and Methods

### 5.1. Study Design and Conceptual Scope

This study was designed as a theoretical and hypothesis-generating modeling analysis with documented internal consistency checks rather than as an empirical metabolomics study, a statistical parameter-fitting exercise, or a calibrated physiological prediction model. No new human participants, animals, tissues, clinical specimens, biological samples, or patient-level datasets were included or analyzed. Institutional review board approval and informed consent were therefore not applicable. The computational framework comprised: (i) a finite-pool algebraic branch-allocation model for the indole-3-acetic acid (IAA)–skatole axis; (ii) a one-dimensional plug-flow-reactor (PFR)-inspired spatial extension preserving directional proximal-to-distal progression and spatially varying ecological permissiveness; (iii) independent scenario parameterization of the Upstream Ecological, Terminal Metabolic, and Host-Protection Locks; (iv) formal evaluation of local mathematical pH sensitivity and deterministic propagation of assumed pH deviation; (v) deterministic analyses of total-pool size, logistic transition parameters, conversion capacity, relative spatial progression, alternative pH trajectories, benchmark-conversion assumptions, and competing non-conversion loss; (vi) derivation and evaluation of composite exposure parameters governing loss-free distal allocation; and (vii) analytical, numerical, boundary-condition, nonnegativity, and mass-balance evaluation of the implemented equations. The model was intentionally formulated as a reduced representation of the intestinal IAA–skatole axis. It did not explicitly simulate microbial production of indole from tryptophan, hepatic conversion of indole to indoxyl sulfate (IS), intestinal absorption into a systemic compartment, plasma protein binding, tissue distribution, renal clearance, tubular secretion, dialysis removal, circulating IS concentrations, individual microbial species, strain-specific growth, microbial competition, dynamic substrate replenishment, epithelial transport kinetics, or host pharmacokinetics. Model outputs represented theoretical allocations within a model-defined IAA-equivalent pool rather than directly measured fecal, luminal, plasma, serum, urinary, intracellular, or tissue concentrations. The term IAA-equivalent pool denotes the model-tracked precursor-equivalent pool centered on IAA because IAA is the immediate microbial precursor in the IAD-associated terminal pathway to skatole. The term does not imply that the entire modeled pool consists of chemically unchanged IAA, that all precursor material is directly measurable as free IAA, or that terminal fecal skatole burden is quantitatively interchangeable with luminal IAA exposure. Similarly, terminal skatole-equivalent allocation denotes the portion of the model-defined available pool assigned to the skatole-associated terminal-conversion branch. This quantity is not a measured skatole concentration, an experimentally fitted conversion yield, a toxicity measure, or a calibrated prediction of skatole concentration in any biological compartment. The model examined structural relationships among environmental pH, indoleacetate decarboxylase (IAD)-associated terminal-conversion capacity, substrate availability, relative spatial progression, and competing non-conversion loss. Internal algebraic and numerical checks assessed consistency of the stated equations, analytical relationships, and numerical implementation. Internal consistency did not constitute experimental, biological, physiological, external, or clinical validation.

### 5.2. Identification and Use of Literature Evidence

The relevant literature was identified through targeted searches of PubMed and publisher websites, supplemented by backward reference checking of relevant original articles and reviews. The final targeted literature search for the present modeling study was conducted on 18 July 2026. Search concepts included chronic kidney disease, the gut–kidney axis, microbial tryptophan metabolism, indole, indoxyl sulfate, indole-3-acetic acid, skatole, indoleacetate decarboxylase, intestinal pH, colonic pH, resistant starch, short-chain fatty acids, proteolytic fermentation, intestinal transit, constipation, and protein-bound uremic toxins. Recent cohort, metagenomic, metabolomic, and mechanistic studies were prioritized when establishing the contemporary biological context. Foundational publications were retained when they provided original biochemical, analytical, enzymatic, microbiological, or physiological evidence relevant to model construction or interpretation. The literature was used to: (i) define the biological scope of the reduced model; (ii) determine the direction of modeled relationships; (iii) establish technically plausible deterministic scenario ranges; (iv) identify interpretation boundaries; and (v) formulate experimentally testable hypotheses. No external experimental or clinical dataset was used for statistical parameter estimation, parameter optimization, empirical model fitting, physiological calibration, or evaluation of predictive accuracy. The literature component was targeted and narrative rather than systematic. No formal systematic-review protocol, study-selection flow diagram, duplicate screening procedure, risk-of-bias assessment, certainty-of-evidence grading, publication-bias analysis, or quantitative evidence synthesis was performed.

### 5.3. Derivation of the Technical Concentration Benchmark

A historical value of approximately 100 μg skatole/g fresh feces, as summarized by Zgarbová and Vrzal from the earlier literature, was selected as an order-of-magnitude technical reference anchor [38]. Zgarbová and Vrzal additionally provided an explicit wet-volume-equivalent calculation based on an assumed fecal density of 1.07 g/mL and an average fecal water content of 75%. Their review was used as a secondary quantitative source that summarized the historical fresh-feces value and its physical conversion assumptions; it was not treated as the primary experimental study that originally measured the historical value. The historical clinical state associated with this value was described as “disturbed intestinal digestion.” This terminology was not defined using contemporary diagnostic criteria and was not treated as equivalent to a specific modern gastrointestinal diagnosis, a uniform dysbiotic state, inflammatory bowel disease, colorectal neoplasia, or another currently defined disorder. Karlin et al. reported elevated fecal skatole burdens in selected colorectal-neoplasia groups, but their concentrations were calculated on a dry-weight basis [46]. Those data were used only as complementary evidence that elevated fecal skatole burdens had been reported in selected gastrointestinal disease contexts. Dry-weight concentrations were not treated as quantitatively interchangeable with the fresh-feces value and were not entered into the wet-volume conversion. The fresh-feces value summarized by Zgarbová and Vrzal was used solely as a transparent, order-of-magnitude technical scaling anchor. It was not treated as a pooled estimate, population mean, population median, diagnostic threshold, population reference limit, universally pathological concentration, or direct measurement of freely dissolved luminal skatole. An effective concentration display scale normalized to an assumed water-accessible fraction, expressed in μM, was calculated as

$$C_{\mathrm{benchmark}}=\frac{B_{\mathrm{fecal}}\rho_{\mathrm{feces}}}{MW_{\mathrm{skatole}}w}\times 1000,$$

where $B_{\mathrm{fecal}}$ is the fecal skatole burden in μg/g fresh feces, $\rho_{\mathrm{feces}}$ is the assumed fecal density in g/mL, $MW_{\mathrm{skatole}}$ is the molecular weight of skatole in ${\mu g\;\mu \mathrm{mol}}^{-1}$, and w is the dimensionless assumed water-accessible fraction. The factor of 1000 converts μmol/mL to μM. The reference values were

- $B_{\mathrm{fecal}}=100\;\mu g/g$;
- $\rho_{\mathrm{feces}}=1.07\;g/\mathrm{mL}$;
- $MW_{\mathrm{skatole}}=131.2{\;\mu g\;\mu \mathrm{mol}}^{-1}$;
- w=0.75.

For this technical calculation, the reported mean fecal water content of 75% was operationally used as the assumed water-accessible fraction w. This substitution was a technical scaling convention and did not establish that all fecal water constituted a freely accessible chemical phase or that total fecal skatole was uniformly distributed within such a phase. Reapplication of these assumptions yielded

$$C_{\mathrm{benchmark}}=\frac{100\times 1.07}{131.2\times 0.75}\times 1000=1087.39837398374\;\mu M,$$

equivalent to approximately 1.087 mM and consistent with the approximate wet-volume-equivalent scale summarized by Zgarbová and Vrzal. The conversion was introduced solely to place the fresh-feces mass-based reference on a concentration-like display scale for deterministic model visualization. It did not define a chemical-equivalence conversion from skatole to IAA. The unrounded computational value was retained in the machine-readable source data. A rounded reference pool of

$$C_{\mathrm{total}}=1000\;\mu M$$

was used for technical visualization, comparison of absolute model outputs, and deterministic scenario analyses. Division by w expressed the total fecal burden relative to an assumed water-accessible subvolume. This operation was a physical scaling assumption rather than a measurement of freely dissolved concentration. It did not imply that the entire fecal skatole burden was dissolved, unbound, biologically available, homogeneously distributed, or located within a directly measured aqueous compartment. The benchmark was derived from a fecal skatole reference and did not establish chemical equivalence between skatole and IAA. Fecal concentration was not equated with intraluminal concentration, terminal metabolite burden was not equated with precursor concentration, and the resulting value was not interpreted as a measured or typical luminal IAA concentration. The rounded 1000 μM reference was retained as a technical model-scaling benchmark whose dependence on the assumed water-accessible fraction and fecal density was evaluated deterministically. The value was not interpreted as a physiological threshold, therapeutic exposure, diagnostic threshold, recommended experimental concentration, or prediction of a human luminal, fecal, plasma, intracellular, or tissue concentration. The value may inform consideration of a candidate upper-bound condition within a future concentration–response design, but the model-derived scale cannot by itself justify selection of an experimental exposure concentration.

### 5.4. Parameterization of the Effective Leakage Function

Because the benchmark calculation depended directly on the assumed water-accessible fraction and fecal density, both quantities were varied in a deterministic two-dimensional scenario analysis. The water-accessible fraction was evaluated at

$$w\in \left\{0.50,\;0.55,\;0.60,\;0.65,\;0.70,\;0.75,\;0.80,\;0.85,\;0.90\right\},$$

and fecal density was evaluated at

$$\rho_{\mathrm{feces}}\in \left\{1.000,\;1.035,\;1.070,\;1.110,\;1.150\right\}\;g/\mathrm{mL}.$$

The complete Cartesian product therefore contained

9×5=45

deterministic technical-assumption conditions.

The reference condition was

$$w=0.75\;\;\mathrm{and}\;\;\rho_{\mathrm{feces}}=1.07\;g/\mathrm{mL}.$$

These ranges were treated as documented deterministic technical-assumption scenarios rather than validated population reference intervals, probability distributions, confidence intervals, or empirically estimated uncertainty bounds. The analysis quantified the dependence of the technical concentration scale on the two conversion assumptions. It did not quantify the complete analytical or biological uncertainty associated with fecal metabolite measurement. The benchmark inputs and resulting range are summarized in Table 1. The complete 45-condition matrix is reported in Supplementary Table S1 and Figure S1. The principal parameters and evaluated ranges used throughout the deterministic analyses are summarized in Table 9.

### 5.5. State Variables, Reference Conditions, and Algebraic Allocation

The model-defined total precursor-equivalent pool was denoted by $C_{\mathrm{total}}$. The fraction of the model-defined total precursor-equivalent pool available to enter the spatial conversion system was controlled by the Host-Protection Lock coefficient $L_{3}$, operationally defined as a dimensionless substrate-availability coefficient:

$$C_{\mathrm{available}}=L_{3}C_{\mathrm{total}}.$$

The complementary excluded pool was

$$C_{\mathrm{excluded}}=\left(1-L_{3}\right)C_{\mathrm{total}}.$$

The excluded pool was retained as an accounting term and was not interpreted as a directly measured chemical, anatomical, absorbed, or protected compartment. The retained IAA-equivalent state was denoted by I, the terminal skatole-equivalent allocation by S, and cumulative competing non-conversion loss by $Q_{\mathrm{loss}}$. Unless otherwise stated, the reference conditions were

$$C_{\mathrm{total}}=1000\;\mu M,\;\;\;L_{2}=L_{3}=1,\;\;k_{\max}=4,\;\;v_{\mathrm{rel}}=1,\;\;{\mathrm{pH}}_{\mathrm{mid}}=6.6,\;\;\mathrm{and}\;s=4.$$

Local ecological permissiveness toward terminal allocation was represented by the bounded logistic function

$$f_{\mathrm{leak}}\left(\mathrm{pH}\right)=\frac{1}{1+\exp\left[-s\left(\mathrm{pH}-{\mathrm{pH}}_{\mathrm{mid}}\right)\right]}.$$

The logistic form was selected because it is bounded between zero and one, monotonic, defined by an interpretable equal-allocation midpoint and adjustable transition slope, analytically invertible, and suitable for transition-width and local-sensitivity analyses. It was not selected on the basis of demonstrated empirical superiority over alternative transition functions. The label leak denoted model-defined allocation toward the terminal branch. It did not denote epithelial leakage, intestinal permeability, or barrier disruption. The complementary retained fraction was

$$f_{\mathrm{retain}}\left(\mathrm{pH}\right)=1-f_{\mathrm{leak}}\left(\mathrm{pH}\right).$$

The algebraic terminal and retained allocations were

$$S_{\mathrm{alg}}=C_{\mathrm{total}}f_{\mathrm{leak}}\left(\mathrm{pH}\right),$$

and

$$I_{\mathrm{alg}}=C_{\mathrm{total}}\left[1-f_{\mathrm{leak}}\left(\mathrm{pH}\right)\right].$$

By construction,

$$I_{\mathrm{alg}}+S_{\mathrm{alg}}=C_{\mathrm{total}}.$$

The logistic function was a phenomenological representation of pH-associated community-level ecological permissiveness. It was not a fitted enzyme-kinetic law, a Hill function derived from binding data, a clinical cutoff, a discontinuous biological threshold, a dynamical bifurcation, or a universal physiological transition point. Algebraic curves were evaluated from pH 5.0 to 8.5 in increments of 0.01 pH units. Focused analyses were performed over pH 6.4–6.8.

### 5.6. Total-Pool Scaling

Dependence of absolute algebraic model outputs on the technical total-pool scale was evaluated using

$$C_{\mathrm{total}}\in \left\{250,\;500,\;1000,\;1500,\;2000\right\}\;\mu M.$$

Absolute and normalized algebraic allocations were calculated across the complete pH 5.0–8.5 grid. Focused outputs were recorded at pH 6.0, 6.4, 6.6, 6.8, 7.0, and 7.4. For the algebraic model,

$$\frac{S_{\mathrm{alg}}}{C_{\mathrm{total}}}=f_{\mathrm{leak}}\left(\mathrm{pH}\right)\;\;\mathrm{and}\;\;\frac{I_{\mathrm{alg}}}{C_{\mathrm{total}}}=1-f_{\mathrm{leak}}\left(\mathrm{pH}\right).$$

Thus, absolute allocation was expected to scale linearly with $C_{\mathrm{total}}$, whereas normalized allocation was expected to remain invariant. For each focused pH value, absolute terminal allocation was additionally evaluated as a linear function of $C_{\mathrm{total}}$. The fitted slope, intercept, coefficient of determination, and maximum residual were used as numerical confirmation of the analytically expected linear scaling. These regressions were numerical linearity checks and not empirical statistical models. Because the loss-free spatial equations were linear and homogeneous in the state variables, absolute spatial states were also expected analytically to scale proportionally with $C_{\mathrm{total}}$ under fixed dimensionless parameters, whereas appropriately normalized fractions remained invariant. Selected representative values are summarized in Table 2. The complete pH grid, including the additional focused pH 7.0 output, is retained in the machine-readable source data. A focused formatted summary is provided in Supplementary Table S2, and the complete 1755-row algebraic grid is provided in the source-data archive.

### 5.7. Local Mathematical pH Sensitivity and Deterministic Perturbation Propagation

The normalized local sensitivity of algebraic terminal allocation was

$$\frac{d}{d\mathrm{pH}}\left(\frac{S_{\mathrm{alg}}}{C_{\mathrm{total}}}\right)=sf_{\mathrm{leak}}\left(1-f_{\mathrm{leak}}\right).$$

The corresponding absolute sensitivity was

$$\frac{dS_{\mathrm{alg}}}{d\mathrm{pH}}=C_{\mathrm{total}}sf_{\mathrm{leak}}\left(1-f_{\mathrm{leak}}\right).$$

Maximum local sensitivity occurred at $\mathrm{pH}={\mathrm{pH}}_{\mathrm{mid}}$, where $f_{\mathrm{leak}}=0.5$. The maximum normalized sensitivity was s/4, and the maximum absolute sensitivity was $C_{\mathrm{total}}s/4$. For a nominal value ${\mathrm{pH}}_{0}$ and a symmetric perturbation magnitude δ, the lower and upper normalized allocations were

$$f_{\mathrm{lower}}=f_{\mathrm{leak}}\left({\mathrm{pH}}_{0}-\delta \right)\;\;\mathrm{and}\;\;f_{\mathrm{upper}}=f_{\mathrm{leak}}\left({\mathrm{pH}}_{0}+\delta \right),$$

respectively. The full normalized perturbation interval was

$$\Delta f_{\mathrm{full}}=f_{\mathrm{upper}}-f_{\mathrm{lower}},$$

and the corresponding absolute interval was

$$\Delta S_{\mathrm{full}}=C_{\mathrm{total}}\Delta f_{\mathrm{full}}.$$

The focused formatted analysis evaluated nominal pH values from 6.40 to 6.80 in increments of 0.05 using $\delta \in \left\{0.10,\;0.20\right\}$, yielding

9×2=18

graphical-source conditions. Supplementary Figure S3 evaluated nominal pH values from 6.40 to 6.80 in increments of 0.05 using $\delta \in \left\{0.05,\;0.10,\;0.15,\;0.20\right\}$, yielding

9×4=36

graphical-source conditions. These analyses represented deterministic dependence on assumed symmetric pH deviations and did not constitute complete models of experimental measurement uncertainty, sampling variability, calibration drift, spatial heterogeneity, temporal variability, or matrix dependence.

### 5.8. Logistic Transition-Parameter Grid

Dependence on the location and steepness of the phenomenological pH transition was evaluated using all combinations of

$${\mathrm{pH}}_{\mathrm{mid}}\in \left\{6.2,\;6.4,\;6.6,\;6.8\right\}\;\;\mathrm{and}\;\;s\in \left\{2,\;4,\;6,\;8\right\}.$$

The complete design contained

4×4=16

parameter combinations. For a target terminal-allocation fraction q, the corresponding pH was

$${\mathrm{pH}}_{q}={\mathrm{pH}}_{\mathrm{mid}}+\frac{1}{s}\ln\left(\frac{q}{1-q}\right).$$

The 10–90% transition width was

$${\Delta \mathrm{pH}}_{10-90}=\frac{2\ln\left(9\right)}{s}.$$

For each parameter combination, the pH values corresponding to 10%, 25%, 50%, 75%, and 90% terminal allocation, maximum local sensitivity, transition width, allocation values at pH 6.4, 6.6, and 6.8, bounds, monotonicity, and pool balance were evaluated. Five documented analytical identities were evaluated for each of the 16 combinations, yielding 80 internal consistency tests. The term equal-allocation reference point was used instead of bifurcation point because the bounded logistic function did not define a dynamical bifurcation. Complete results are reported in Supplementary Table S3A and Figure S2.

### 5.9. Triple-Lock Parameterization

The term “Lock” was used as a conceptual label for a modeled constraint and did not imply that the corresponding biological mechanism had been independently identified, quantified, or experimentally validated.

#### 5.9.1. Upstream Ecological Lock

The Upstream Ecological Lock was implemented through the spatially varying term $f_{\mathrm{leak}}\left[\mathrm{pH}\left(x\right)\right]$, where $x\in \left[0,1\right]$ denotes normalized proximal-to-distal spatial progression. This term represented pH-associated ecological permissiveness toward terminal allocation. It did not explicitly simulate microbial taxonomic composition, strain-level variation, microbial growth, fermentable-substrate depletion, short-chain fatty acid production, proteolytic fermentation, *tnaA* expression, tryptophanase activity, cross-feeding, or individual microbial reactions.

#### 5.9.2. Terminal Metabolic Lock

The Terminal Metabolic Lock was represented by the dimensionless scenario coefficient $L_{2}\in \left[0,1\right]$. The condition $L_{2}=1$ defined the unrestricted reference conversion scenario, whereas progressively lower values represented increasing restriction of terminal-conversion capacity. The condition $L_{2}=0$ eliminated terminal conversion in the absence of an alternative source term for S. $L_{2}$ was not a direct measurement of IAD gene abundance, transcript abundance, protein abundance, enzyme concentration, catalytic efficiency, substrate affinity, maximum enzymatic velocity, microbial biomass, or in situ pathway flux.

#### 5.9.3. Host-Protection Lock

The Host-Protection Lock was represented operationally by the dimensionless substrate-availability coefficient $L_{3}\in \left[0,1\right]$. The dynamically available and excluded pools were

$$C_{\mathrm{available}}=L_{3}C_{\mathrm{total}}\;\;\mathrm{and}\;\;C_{\mathrm{excluded}}=\left(1-L_{3}\right)C_{\mathrm{total}}$$

respectively. $L_{3}$ therefore scales the model-defined precursor-equivalent pool available to enter the spatial conversion system. It does not directly quantify epithelial apoptosis, epithelial permeability, epithelial turnover, barrier disruption, mucus integrity, butyrate concentration, luminal protein release, cellular extrusion, absorption, or any single host-protection mechanism. The term “Host-Protection Lock” is a conceptual label for this model layer, whereas $L_{3}$ is its operational substrate-availability coefficient. Joint Triple-Lock analyses evaluated $L_{2},L_{3}\in \left\{0,\;0.25,\;0.50,\;0.75,\;1.00\right\}$. All 25 $L_{2}\times L_{3}$ combinations were evaluated under constant pH 6.0 and the linear pH 6.2–7.4 profile, yielding

25×2=50

Triple-Lock scenarios. The complete grid is provided in Supplementary Table S5.

### 5.10. Treatment of the $L_{3}=0$ Boundary

At $L_{3}=0$, the available pool was $C_{\mathrm{available}}=0$. The dynamic states were $I\left(x\right)=0$, $S\left(x\right)=0$, and, in analyses initialized without a dynamically available pool, $Q_{\mathrm{loss}}\left(x\right)=0$. The excluded pool was $C_{\mathrm{excluded}}=C_{\mathrm{total}}$. The normalized response $\frac{S\left(1\right)}{C_{\mathrm{available}}}$ was undefined because both the numerator and denominator were zero. The resulting 0/0 boundary was not assigned a numerical value of zero. In formatted tables, the undefined ratio was represented by an em dash. In machine-readable files, it was retained as a blank or missing value. The $L_{3}=0$ boundary was excluded from analyses requiring normalization by $C_{\mathrm{available}}$, but it was retained as an explicit boundary-condition test.

### 5.11. Loss-Free One-Dimensional Spatial Model

Spatial redistribution was modeled over the normalized coordinate $x\in \left[0,1\right]$, where x=0 represents the proximal boundary and x=1 represents the distal boundary. The coordinate was dimensionless and did not represent a calibrated physical distance or elapsed time. Relative spatial progression was represented by the dimensionless coefficient $v_{\mathrm{rel}}$, with relative spatial residence proportional to $\frac{1}{v_{\mathrm{rel}}}$. The effective local terminal-conversion coefficient was

$$k_{\mathrm{eff}}\left(x\right)=\frac{k_{\max}L_{2}}{v_{\mathrm{rel}}}f_{\mathrm{leak}}\left[\mathrm{pH}\left(x\right)\right].$$

The loss-free spatial model was

$$\frac{dI}{dx}=-k_{\mathrm{eff}}\left(x\right)I\left(x\right),$$

and

$$\frac{dS}{dx}=k_{\mathrm{eff}}\left(x\right)I\left(x\right).$$

The numerical state vector for the loss-free system was

$$y\left(x\right)={\left[I\left(x\right),S\left(x\right)\right]}^{T}.$$

Initial conditions were $I\left(0\right)=C_{\mathrm{available}}$ and $S\left(0\right)=0$. Available-pool conservation required

$$I\left(x\right)+S\left(x\right)=C_{\mathrm{available}},$$

and complete accounting required

$$I\left(x\right)+S\left(x\right)+C_{\mathrm{excluded}}=C_{\mathrm{total}}.$$

Because x and $v_{\mathrm{rel}}$ were normalized, $k_{\max}$ was interpreted as a dimensionless spatial conversion coefficient rather than an experimentally measured time-based kinetic constant. The PFR-inspired formulation represented directional spatial progression without explicit axial mixing, segmental exchange, or anatomically calibrated geometry. It was not intended as an individualized reconstruction of the human colon.

### 5.12. Analytical Loss-Free Solution and Composite Exposure

Integrated pH-dependent permissiveness up to position x was defined as

$$A_{\mathrm{pH}}\left(x\right)=\int_{0}^{x}f_{\mathrm{leak}}\left[\mathrm{pH}\left(\xi \right)\right]\;d\xi .$$

Full-profile integrated permissiveness was $A_{\mathrm{pH}}=A_{\mathrm{pH}}\left(1\right)$. The composite conversion–progression coefficient was

$$\Lambda_{\mathrm{conv}}=\frac{k_{\max}L_{2}}{v_{\mathrm{rel}}},$$

and the integrated loss-free exposure was

$$\Psi =\Lambda_{\mathrm{conv}}A_{\mathrm{pH}}.$$

The analytical loss-free solutions were

$$I\left(x\right)=C_{\mathrm{available}}\exp\left[-\Lambda_{\mathrm{conv}}A_{\mathrm{pH}}\left(x\right)\right],$$

and

$$S\left(x\right)=C_{\mathrm{available}}\left\{1-\exp\left[-\Lambda_{\mathrm{conv}}A_{\mathrm{pH}}\left(x\right)\right]\right\}.$$

For $C_{\mathrm{available}}>0$, the distal available-pool-normalized response was

$$\frac{S\left(1\right)}{C_{\mathrm{available}}}=1-\exp\left(-\Psi \right).$$

Different combinations of $k_{\max}$, $L_{2}$, and $v_{\mathrm{rel}}$ producing the same $\Lambda_{\mathrm{conv}}$ were evaluated under common pH profiles. Equal- $\Lambda_{\mathrm{conv}}$ output equivalence indicated endpoint-level structural non-identifiability under the reduced loss-free first-order formulation and a fixed pH profile. It did not imply biological interchangeability or non-identifiability if intermediate trajectories, additional observables, competing loss, replenishment, or more complex dynamics were measured. The equal-$\Lambda_{\mathrm{conv}}$ trajectory comparison used

$$\left(k_{\max},L_{2},v_{\mathrm{rel}}\right)=\left(1,1,0.5\right),\;\left(k_{\max},L_{2},v_{\mathrm{rel}}\right)=\left(2,1,1\right),\;\;\mathrm{and}\;\left(k_{\max},L_{2},v_{\mathrm{rel}}\right)=\left(4,1,2\right),$$

each yielding $\Lambda_{\mathrm{conv}}=2$. Each trajectory was reported at 301 spatial coordinates, giving 903 coordinate-level records for the three-combination comparison. For visualization of the analytical integrated-exposure relationship in Figure 3D, $A_{\mathrm{pH}}$ was evaluated from 0.300 to 0.860 in increments of 0.002, yielding 281 analytical points.

### 5.13. Reference Constant and Linear pH Profiles

The two primary reference profiles were constant pH 6.0,

$$\mathrm{pH}\left(x\right)=6.0,$$

and a linear profile increasing from pH 6.2 to 7.4,

$$\mathrm{pH}\left(x\right)=6.2+1.2x.$$

Additional constant profiles were evaluated at pH 6.4, 6.6, and 7.4. A general linear profile was expressed as

$$\mathrm{pH}\left(x\right)={\mathrm{pH}}_{\mathrm{proximal}}+\left({\mathrm{pH}}_{\mathrm{distal}}-{\mathrm{pH}}_{\mathrm{proximal}}\right)x.$$

The broader linear-profile analysis evaluated proximal pH values of $\left\{6.0,6.2,6.4\right\}$ and distal pH values of $\left\{6.6,\;6.8,\;7.0,\;7.2,\;7.4\right\}$, subject to ${\mathrm{pH}}_{\mathrm{distal}}>{\mathrm{pH}}_{\mathrm{proximal}}$. The resulting Cartesian product contained 15 valid proximal–distal combinations. An effective constant pH was defined as the constant pH value producing the same integrated permissiveness as the evaluated spatial profile:

$$f_{\mathrm{leak}}\left({\mathrm{pH}}_{\mathrm{eff}}\right)=A_{\mathrm{pH}}.$$

Thus,

$${\mathrm{pH}}_{\mathrm{eff}}={\mathrm{pH}}_{\mathrm{mid}}+\frac{1}{s}\ln\left(\frac{A_{\mathrm{pH}}}{1-A_{\mathrm{pH}}}\right).$$

${\mathrm{pH}}_{\mathrm{eff}}$ was a logistic-equivalent constant pH and not an arithmetic mean, geometric mean, profile midpoint, or directly measured physiological quantity. Supplementary Figure S4C compared linear pH 6.0–7.2, linear pH 6.2–7.0, linear pH 6.4–6.8, and constant pH 6.6, each having $A_{\mathrm{pH}}=0.5$. Each trajectory was reported at 301 spatial coordinates.

### 5.14. Nonlinear pH-Profile Robustness

A broader profile-shape robustness analysis evaluated a linear pH 6.2–7.4 reference profile and eight nonlinear profile families:

- Early-rise square-root;
- Late-rise quadratic;
- Normalized symmetric sigmoid;
- Cubic smoothstep;
- Proximal plateau followed by distal rise;
- Early rise followed by distal plateau;
- Mildly undulating nonmonotonic;
- Sinusoidal ease.

Each nonlinear profile was evaluated in forward, reversed, and exposure-matched forms. For a forward profile ${\mathrm{pH}}_{j}\left(x\right)$, the reversed profile was

$${\mathrm{pH}}_{j,\mathrm{rev}}\left(x\right)={\mathrm{pH}}_{j}\left(1-x\right).$$

Exposure-matched profiles were generated by adding a profile-specific constant offset $\Delta_{j}$:

$${\mathrm{pH}}_{j,\mathrm{matched}}\left(x\right)={\mathrm{pH}}_{j}\left(x\right)+\Delta_{j},$$

where $\Delta_{j}$ was selected so that

$$\int_{0}^{1}f_{\mathrm{leak}}\left[{\mathrm{pH}}_{j,\mathrm{matched}}\left(x\right)\right]\;dx=A_{\mathrm{pH},\mathrm{linear}}.$$

Adaptive quadrature was used to evaluate $A_{\mathrm{pH}}$ for the documented profile functions. Exposure-matching offsets used in the revision-stage supporting analyses were determined numerically from the documented logistic formulation and the profile-specific integrated-permissiveness target. The resulting offsets, adjusted profile endpoints, integrated-permissiveness values, and distal outputs are provided in the machine-readable source data. The retained machine-readable source data report available profile-specific offsets, integrated-permissiveness values, adjusted profile endpoints, and distal outputs. Exposure-matched profiles were not required to preserve the original pH 6.2–7.4 endpoints. Under the specified loss-free first-order formulation, forward and reversed profiles having the same $A_{\mathrm{pH}}$ produced the same distal endpoint, although intermediate trajectories could differ. This endpoint equivalence was a property of the specified loss-free first-order integral structure and did not imply that spatial ordering is biologically irrelevant in the intestine. Spatial order could affect outcomes in models incorporating substrate replenishment, segmental uptake, axial mixing, feedback, or other nonlinear spatial interactions.

### 5.15. Conversion-Capacity and Relative-Progression Analyses

Focused deterministic comparisons evaluated

$$k_{\max}\in \left\{1,\;2,\;4,\;6,\;8\right\}\;\;\mathrm{and}\;\;v_{\mathrm{rel}}\in \left\{0.5,\;1,\;2\right\}.$$

The conditions $v_{\mathrm{rel}}=0.5$ and $v_{\mathrm{rel}}=2$ represented twofold prolonged and twofold shortened relative spatial residence, respectively, compared with the $v_{\mathrm{rel}}=1$ reference. These values were not calibrated to measured gastrointestinal transit times, stool frequency, constipation severity, or clinical categories. For the high-density plotting source used in Supplementary Figure S5A,B, $L_{2}=1$ and $L_{3}=1$ were retained. The coefficient $k_{\max}$ was evaluated from 1.00 to 8.00 in increments of 0.05, giving 141 levels. The coefficient $v_{\mathrm{rel}}$ was evaluated from 0.50 to 2.00 in increments of 0.01, giving 151 levels. The complete plotting design therefore contained

141×151×2=42,582

records across the constant pH 6.0 and linear pH 6.2–7.4 panels. This high-density graphical grid was distinct from the focused five-level $k_{\max}$ and three-level $v_{\mathrm{rel}}$ comparison and from the analytical scenario-collapse grid.

### 5.16. Joint pH–$L_{2}$ Response Surface

A two-dimensional analytical response surface was generated under constant-pH, loss-free conditions. Constant pH was evaluated from 6.00 to 7.50 in increments of 0.01, giving 151 pH levels. $L_{2}$ was evaluated from 0.00 to 1.00 in increments of 0.01, giving 101 levels. The complete surface contained

151×101=15,251

analytical conditions. The response was

$$\frac{S\left(1\right)}{C_{\mathrm{available}}}=1-\exp\left[-k_{\max}L_{2}f_{\mathrm{leak}}\left(\mathrm{pH}\right)\right],$$

using $k_{\max}=4,v_{\mathrm{rel}}=1,L_{3}=1$. A documented set of 70 boundary, transition-region, and interior conditions was compared with numerical ordinary differential equation solutions. The retained condition set spanned the $\;L_{2}=0$ and $L_{2}=1$ boundaries, low- and high-pH boundaries, points within the principal transition region, and interior response-surface locations. The exact 70-condition record contains pH, $L_{2}$, analytical response, numerical response, residual, criterion, and status. The complete 70-condition analytical–numerical comparison is provided in the V06 current-supporting record within “Supplementary_Verification_Audit.zip”. The surface was not interpreted as biological synergy, pharmacological synergy, statistical interaction, effect modification, or causal interaction.

### 5.17. Competing Non-Conversion Loss

A phenomenological competing-loss compartment represented aggregate removal from the tracked precursor-equivalent state space through processes not separately modeled. The nominal isolated cumulative-loss fraction was denoted by l, and the corresponding first-order loss coefficient was

$$k_{\mathrm{loss}}=-\ln\left(1-l\right).$$

The competing conversion–loss system was

$$\frac{dI}{dx}=-\left\{\frac{k_{\max}L_{2}f_{\mathrm{leak}}\left[\mathrm{pH}\left(x\right)\right]+k_{\mathrm{loss}}}{v_{\mathrm{rel}}}\right\}I\left(x\right),$$

$$\frac{dS}{dx}=\frac{k_{\max}L_{2}f_{\mathrm{leak}}\left[\mathrm{pH}\left(x\right)\right]}{v_{\mathrm{rel}}}I\left(x\right),$$

and

$$\frac{dQ_{\mathrm{loss}}}{dx}=\frac{k_{\mathrm{loss}}}{v_{\mathrm{rel}}}I\left(x\right).$$

The numerical state vector was

$$y\left(x\right)={\left[I\left(x\right),S\left(x\right),Q_{\mathrm{loss}}\left(x\right)\right]}^{T}.$$

Initial conditions were $I\left(0\right)=C_{\mathrm{available}}$, $S\left(0\right)=0$, and $Q_{\mathrm{loss}}\left(0\right)=0$. Complete accounting required

$$I\left(x\right)+S\left(x\right)+Q_{\mathrm{loss}}\left(x\right)+C_{\mathrm{excluded}}=C_{\mathrm{total}}.$$

$Q_{\mathrm{loss}}$ combined unmodeled removal from the modeled luminal state space through absorption, degradation, diversion into alternative pathways, or other processes. Absorption represented removal from the modeled luminal state space but not necessarily removal from the host system. The nominal value l represented cumulative loss for an isolated loss-only process at $v_{\mathrm{rel}}=1$. Realized loss was not assumed to equal l when loss and conversion competed for the same precursor pool.

### 5.18. Combined Relative-Progression–Loss Analysis

Combined analyses evaluated

$$v_{\mathrm{rel}}\in \left\{0.5,\;1,\;2\right\}\;\;\mathrm{and}\;\;l\in \left\{0,\;0.10,\;0.25,\;0.50\right\},$$

under constant pH 6.0 and linear pH 6.2–7.4. The complete design contained

3×4×2=24

conditions. For each condition, the retained endpoint-level dataset included $I\left(1\right),S\left(1\right),Q_{\mathrm{loss}}\left(1\right)$, their total-pool-normalized fractions,

$$\frac{I\left(1\right)}{C_{\mathrm{total}}},\;\;\frac{S\left(1\right)}{C_{\mathrm{total}}},\;\;\frac{Q_{\mathrm{loss}}\left(1\right)}{C_{\mathrm{total}}},$$

the conditional terminal fraction

$$\frac{S\left(1\right)}{I\left(1\right)+S\left(1\right)},$$

and the maximum mass-balance residual recorded for the condition. The conditional ratio described terminal allocation among non-lost distal material and was not interpreted as the fraction of the original total or available pool converted to skatole. The current 24-condition dataset provides a condition-specific endpoint assessment of mass balance across the reported combinations of reference pH profile, relative spatial progression, and nominal competing-loss fraction. This dataset is distinct in analytical scope from the fixed aggregate V11 verification summary and serves a complementary verification purpose. Spatial trajectories at 301 reporting coordinates are supplied separately for the two conditions represented in Supplementary Figure S6A,B. The complete 24-condition endpoint grid is reported in Supplementary Table S6. Figure 6A,B display selected presentation subsets of this complete grid. The panel-specific subset definitions are provided in the corresponding figure legend.

### 5.19. Analytical Scenario-Collapse Grid

The exact loss-free analytical relationship

$$\frac{S\left(1\right)}{C_{\mathrm{available}}}=1-\exp\left(-\Psi \right)$$

was evaluated across a factorial grid of 5400 scenarios. Ten pH profiles were included:

- Constant pH 6.0;
- Constant pH 6.6;
- Linear pH 6.2–7.4;
- Early-rise square-root;
- Late-rise quadratic;
- Normalized symmetric sigmoid;
- Cubic smoothstep;
- Proximal plateau followed by distal rise;
- Early rise followed by distal plateau;
- Mildly undulating nonmonotonic.

The remaining dimensions were

- $C_{\mathrm{total}}\in \left\{500,\;1000,\;2000\right\}\;\mu M$;
- $L_{2}\in \left\{0.25,\;0.50,\;0.75,\;1.00\right\}$;
- $L_{3}\in \left\{0.25,\;0.50,\;1.00\right\}$;
- $k_{\max}\in \left\{1,\;2,\;4,\;6,\;8\right\}$;
- $v_{\mathrm{rel}}\in \left\{0.5,\;1,\;2\right\}$.

The Cartesian product contained

10×3×4×3×5×3=5400

analytical scenarios. For each scenario,

$$\Psi =\frac{k_{\max}L_{2}}{v_{\mathrm{rel}}}A_{\mathrm{pH}}\;\;\mathrm{and}\;\;{\hat{S}}_{\mathrm{norm}}=1-\exp\left(-\Psi \right)$$

were calculated. Normalization removed direct dependence on positive $L_{3}$ and $C_{\mathrm{total}}$ in the loss-free linear formulation. The $L_{3}=0$ boundary was excluded because the normalized response was undefined. The complete 5400-scenario analytical grid provides a machine-readable evaluation of the exact closed-form scenario-collapse relationship across the documented combinations of pH profile, conversion capacity, Terminal Metabolic Lock coefficient, relative spatial progression, total-pool scale, and positive Host-Protection Lock coefficient. V07 and V08 report the fixed aggregate verification results for 600 endpoint ODE runs in the scenario-collapse analysis. During revision, the complete analytical grid and selected supporting calculations were generated from the documented equations, parameter settings, and analysis conditions to improve procedural transparency. These supporting calculations confirm the stated closed-form relationship and do not replace, revise, or redefine the fixed V07 and V08 values.

### 5.20. Range-Conditional One-at-a-Time Parameter-Influence Analysis

Parameter influence was evaluated by deterministic one-at-a-time variation, with all other quantities retained at their reference conditions. The primary structural response was

$$Y=\frac{S\left(1\right)}{C_{\mathrm{available}}}.$$

For parameter $\theta_{j}$, the influence range was

$$R_{j}=\underset{\theta_{j}}{\max}\left(Y\right)-\underset{\theta_{j}}{\min}\left(Y\right).$$

Reference-scaled influence was

$$R_{j,\mathrm{scaled}}=\frac{R_{j}}{\underset{k}{\max}R_{k}}.$$

The 11 evaluated quantities and ranges were:

- Constant pH: The documented monotonic interval from pH 6.0 to 7.5, with the influence range determined from the analytical endpoint responses;
- ${\mathrm{pH}}_{\mathrm{mid}}$: 6.2, 6.4, 6.6, and 6.8;
- Transition steepness s: 2,4,6, and 8;
- $C_{\mathrm{total}}$: 250, 500, 1000, 1500, and 2000 μM;
- $k_{\max}$: 1, 2, 4, 6, and 8;
- Proximal pH: 6.0,6.2, and 6.4;
- Distal pH: 6.6, 6.8, 7.0, 7.2, and 7.4, subject to distal pH exceeding proximal pH;
- $v_{\mathrm{rel}}$: 0.5,1, and 2;
- $L_{2}$: 0, 0.25, 0.50, 0.75, and 1.00;
- Positive $L_{3}$: 0.25, 0.50, 0.75, and 1.00;
- l: 0, 0.10, 0.25, and 0.50.

The constant-pH response was monotonic over the documented interval from pH 6.0 to 7.5. Its influence range was therefore determined from the analytical endpoint responses at pH 6.0 and 7.5:

$$R_{\mathrm{constant}\;\mathrm{pH}}=0.6966202337.$$

This interval was distinct from the discrete constant profiles used in the reference-profile analysis. Unless the parameter under examination altered a reference condition, the fixed reference values were

$$C_{\mathrm{total}}=1000\;\mu M,\;{\mathrm{pH}}_{\mathrm{mid}}=6.6,\;\;s=4,\;\;k_{\max}=4,\;\;v_{\mathrm{rel}}=1,\;\;L_{2}=L_{3}=1,\;\;l=0.$$

The resulting ranges depended on the evaluated range, parameter scale, transformation, and reference condition. They did not assess parameter interactions or assume probability distributions and were not variance-based global sensitivity indices, Sobol indices, statistical effect sizes, causal effects, uncertainty rankings, parameter-identifiability estimates, or universal rankings of biological importance. The final normalized influence ranges are reported in Supplementary Table S7 and Figure 6C.

### 5.21. Numerical Implementation

Calculations were performed using Python 3.12.9, NumPy 1.26.4, SciPy 1.13.1, pandas 2.2.2, and Matplotlib 3.8.4. The ordinary differential equation systems were solved with “scipy.integrate.solve_ivp” using the implicit Radau method over $x\in \left[0,1\right]$. Radau was selected as a numerically stable adaptive integrator that could be applied consistently across reference, boundary, and competing-loss scenarios. Analytical solutions were evaluated independently wherever available. Unless otherwise specified for the solver-tolerance analysis, the reference numerical settings documented for numerical verification were

$$\mathrm{rtol}=10^{-10}\;\;\mathrm{and}\;\;\mathrm{atol}=10^{-12}.$$

Relative tolerance was dimensionless, whereas absolute tolerance was applied on the μM-scaled state-variable scale. Spatial outputs were reported at 301 equally spaced coordinates. The reporting coordinates were used for trajectory recording and comparison and did not imply that adaptive integration was restricted to fixed spatial increments. Adaptive numerical quadrature was used for integrated-permissiveness calculations involving nonlinear profiles, plateaus, or slope discontinuities. Exposure-matching offsets used in the revision-stage supporting analyses were determined numerically from the documented logistic formulation and profile-specific integrated-permissiveness target. The resulting offsets, profiles, and endpoint values are provided in the machine-readable source data. For two state trajectories $a\left(x\right)$ and $b\left(x\right)$, the full-curve maximum difference was

$$D_{\max}=\underset{j,x}{\max}\left|a_{j}\left(x\right)-b_{j}\left(x\right)\right|,$$

where j indexed the applicable state variables. The default floating-point representation was 64-bit. No random-number generation was used; therefore, no random seed was required. Negative numerical state values were not replaced by zero or otherwise clipped before nonnegativity or mass-balance evaluation. All 24 retained solver-tolerance runs satisfied the documented audit criterion. Machine-readable files retained full computational precision. Values in formatted tables and narrative text were rounded for readability.

### 5.22. Solver-Tolerance Sensitivity

Solver-tolerance sensitivity was evaluated using four tolerance pairs.

- Relaxed: $\mathrm{rtol}=10^{-6},\;\mathrm{atol}=10^{-8}$.
- Standard: $\mathrm{rtol}=10^{-8},\;\mathrm{atol}=10^{-10}$.
- Strict: $\mathrm{rtol}=10^{-10},\;\mathrm{atol}=10^{-12}$.
- Very strict: $\mathrm{rtol}=10^{-12},\;\mathrm{atol}=10^{-14}$.

The very strict solution was used as the numerical reference. Six scenarios were evaluated:

- ST01: Constant pH 6.0, $\;v_{\mathrm{rel}}=1$, and l=0;
- ST02: Linear pH 6.2–7.4, $\;v_{\mathrm{rel}}=1$, and l=0;
- ST03: Linear pH 6.2–7.4,$\;v_{\mathrm{rel}}=1$, and l=0.50;
- ST04: Constant pH 6.6, $\;v_{\mathrm{rel}}=2$, and l=0;
- ST05: Linear pH 6.2–7.4, $\;v_{\mathrm{rel}}=0.5$, and l=0;
- ST06: Linear pH 6.2–7.4, $\;v_{\mathrm{rel}}=0.5$, and l=0.50.

The design contained

4×6=24

solver-tolerance runs. The documented V09 acceptance criterion was

$$D_{\max}\leq 2.0\times 10^{-4}\;\mu M.$$

The fixed authoritative V09 summary reported a maximum full-curve difference of

$$1.0919834409151\times 10^{-4}\;\mu M,$$

whereas the maximum retained in the current 24-run audit was

$$1.0919834949163487\times 10^{-4}\;\mu M.$$

The fixed authoritative V09 summary reported the manuscript-level maximum full-curve difference used in Table 7 and Figure 6D. During revision, the 24-run solver-tolerance assessment was generated as a supporting calculation using the documented representative scenarios and tolerance settings. The current supporting audit confirms the PASS classification under the predefined V09 criterion. Any minor difference in the recorded maximum reflects the scope and implementation of the supporting assessment and does not replace, revise, or redefine the fixed authoritative V09 value.

### 5.23. Internal Verification and Record Classification

The fixed authoritative V01–V12 verification summary is the manuscript-level reporting record used consistently in Table 7, Supplementary Table S8, Figure 6D, and the corresponding machine-readable source data. The complete summary is provided as “verification/verification_summary.csv”. It records the check identifier, verification scope, criterion, tolerance, observed value, unit, observed-to-tolerance ratio where applicable, status, and concise interpretation for each predefined check. During revision, selected supporting records were newly generated or reconstructed from the documented equations, parameter settings, analysis conditions, and numerical-output definitions to improve procedural transparency and facilitate independent inspection. These records include supporting calculations for V03–V06, the 24-run solver-tolerance assessment for V09, and the 1505-location coordinate-level procedural-support record for V10. Supporting and reconstructed records do not replace, revise, or redefine the fixed authoritative V01–V12 summary values. The file “audit/verification_inventory.csv” maps each verification identifier to the fixed authoritative summary, the corresponding supporting record where applicable, the relevant manuscript elements, supporting-file locations, and the role and analytical scope of each supplied record. The inventory provides record classification and package navigation and does not replace “verification/verification_summary.csv”. For the purposes of this study, “authoritative” denotes the fixed aggregate verification values used consistently in the manuscript, tables, figures, and machine-readable verification summary. “Supporting” denotes calculations supplied to facilitate inspection of specific identities, numerical comparisons, solver settings, or analysis conditions. “Reconstructed” denotes procedural supporting records generated during revision from the documented mathematical formulation and analysis settings. These classifications distinguish the role and analytical scope of the supplied records. All verification checks assess internal mathematical and numerical consistency within their documented scopes. They do not constitute experimental, biological, physiological, external, or clinical validation.

### 5.24. Current Supporting Verification Calculations

The calculations described in this section were generated during revision from the documented equations, parameter settings, analysis conditions, and numerical-output definitions to provide additional procedural support. They are complementary to the fixed authoritative V01–V12 summary and do not replace, revise, or redefine the manuscript-level verification values. Current supporting calculations included

- V03, 80 logistic-identity tests;
- V04, 10 profile-integral evaluations;
- V05, 10 profile-reversal comparisons;
- V06, 70 joint pH–$L_{2}$ analytical–numerical conditions;
- V09, 24 solver-tolerance runs.

These revision-stage supporting records facilitate procedural inspection and do not replace, revise, or redefine the fixed authoritative manuscript-level values. Supplementary Table S8 reports the fixed scope, criterion, tolerance, observed value, unit, status, observed-to-tolerance ratio, and interpretation for each verification item. The verification inventory documents the fixed authoritative summaries, the corresponding supporting calculations or revision-stage procedural support where applicable, record classifications, manuscript mappings, supporting-file locations, and the role and analytical scope of each supplied record.

### 5.25. Fixed Aggregate Verification Summaries and Revision-Stage Support for V07 and V08

V07 and V08 report the fixed aggregate verification results for 600 endpoint ordinary differential equation runs in the scenario-collapse analysis. The fixed V07 maximum residual was $4.74192352051261\times 10^{-11}$, and the fixed V08 root-mean-square error was $7.08559529112084\times 10^{-12}$. Both quantities were dimensionless and satisfied their predefined acceptance criteria. During revision, the complete 5400-scenario analytical grid and selected supporting calculations were generated from the documented equations, parameter settings, and analysis conditions to improve procedural transparency and facilitate independent inspection. These revision-stage records provide a machine-readable evaluation of the exact closed-form scenario-collapse relationship. They are complementary to the fixed aggregate V07 and V08 verification summaries and do not replace, revise, or redefine those values.

### 5.26. Revision-Stage Procedural Support for V10

The fixed authoritative V10 summary reports the aggregate analytical–numerical full-curve agreement result used in Table 7, Supplementary Table S8, and Figure 6D. During revision, the file “verification/reconstructed_support/V10_full_curve_analytical_numerical_verification_1505_RECONSTRUCTED.csv” was reconstructed from the documented equations, parameter settings, analysis conditions, and numerical-output definitions to provide coordinate-level procedural support. The reconstructed file contains 1505 coordinate-level records and reports the analytical and numerical states used for procedural inspection of the full-curve comparison. The suffix “_RECONSTRUCTED” identifies the file as revision-stage procedural support and distinguishes its role from that of the fixed aggregate V10 summary. The reconstructed record supports inspection of the analytical–numerical comparison and does not replace, revise, or redefine the authoritative V10 value.

### 5.27. Scope of V11 and V12 Supporting Records

The fixed authoritative V11 and V12 summaries report aggregate verification results for mass balance and state nonnegativity, respectively. The V11 summary reports the fixed maximum absolute mass-balance residual for its documented verification scope, and the V12 summary reports the fixed most-negative-state magnitude for the corresponding scope. These values are used consistently in Table 7, Supplementary Table S8, Figure 6D, and the machine-readable verification summary. During revision, the current 24-condition progression–loss dataset was generated as a separate condition-specific assessment of mass balance across the reported combinations of reference pH profile, relative spatial progression, and nominal competing-loss fraction. This dataset is distinct in analytical scope from the aggregate V11 verification summary. The condition-specific dataset and the aggregate summary therefore serve complementary verification purposes and are not expected to yield an identical maximum residual. Spatial trajectories at 301 reporting coordinates are supplied separately for the two conditions represented in Supplementary Figure S6A,B. The 24-condition endpoint results are reported in Supplementary Table S6. These supporting calculations do not replace, revise, or redefine the fixed authoritative V11 or V12 summary values.

### 5.28. Treatment and Reporting of Deterministic Outputs

All model analyses were deterministic. No random sampling, Monte Carlo simulation, Bayesian inference, data imputation, null-hypothesis significance testing, confidence intervals, multiple-comparison procedures, or P values were applied. Outputs were summarized using

- Absolute and normalized allocations;
- Analytical derivatives;
- Deterministic pH-perturbation intervals;
- Deterministic scenario ranges;
- Distal state values;
- Response surfaces;
- Parameter-influence ranges;
- Analytical–numerical discrepancies;
- Mass-balance residuals;
- State-nonnegativity checks;
- Solver diagnostics.

Terms such as increase, decrease, higher, lower, transition, and sensitivity referred to deterministic contrasts or mathematical properties of the specified model. These terms did not imply statistical significance, empirical association, biological causality, treatment effects, or clinical relevance. Joint response surfaces were not interpreted as evidence of biological or pharmacological synergy, statistical interaction, effect modification, or causal interaction.

### 5.29. Interpretation Boundaries

The following interpretation boundaries were applied throughout the study:

- The 1000 μM reference was a technical model-scaling benchmark and not a measured physiological IAA concentration.
- The fecal skatole anchor did not establish chemical equivalence between skatole and IAA.
- The technical benchmark did not identify a measured freely dissolved or water-accessible biological compartment.
- The reported mean fecal water content was operationally used as the assumed water-accessible fraction and did not establish that the entire fecal aqueous phase was chemically accessible.
- The benchmark was not a diagnostic threshold, population reference limit, recommended experimental concentration, therapeutic exposure, or universal pathological concentration.
- The historical term “disturbed intestinal digestion” was not treated as a contemporary diagnostic category.
- Dry-weight fecal skatole values and fresh-feces values were not treated as quantitatively interchangeable.
- IAA-equivalent and terminal skatole-equivalent allocations were model-defined quantities and not measured biological concentrations.
- $f_{\mathrm{leak}}$ represented phenomenological ecological permissiveness and not epithelial permeability, barrier leakage, purified enzyme kinetics, or a universal biological threshold.
- ${\mathrm{pH}}_{\mathrm{mid}}$ was an equal-allocation reference point and not a clinical cutoff or dynamical bifurcation.
- $L_{2}$ was not measured IAD abundance, expression, protein level, catalytic activity, or pathway flux.
- $L_{3}$ was not a direct biomarker of epithelial injury, permeability, turnover, apoptosis, butyrate exposure, or barrier integrity.
- $C_{\mathrm{excluded}}$ was an accounting term and not a measured biological compartment.
- $\frac{S\left(1\right)}{C_{\mathrm{available}}}$ was defined only when $C_{\mathrm{available}}>0$.
- $v_{\mathrm{rel}}$ and $\frac{1}{v_{\mathrm{rel}}}$ were dimensionless relative quantities and were not calibrated to absolute transit time, stool frequency, constipation severity, or clinical motility categories.
- $k_{\max}$ and $\Lambda_{\mathrm{conv}}$ were dimensionless model coefficients and not measured time-based kinetic constants.
- $k_{\max}$, $L_{2}$, and $v_{\mathrm{rel}}$ were structurally linked through $\Lambda_{\mathrm{conv}}$.
- $A_{\mathrm{pH}}$, ${\mathrm{pH}}_{\mathrm{eff}}$, and Ψ were model quantities and not physiological biomarkers or doses.
- $Q_{\mathrm{loss}}$ combined multiple unmodeled removal processes and could not identify their individual contributions.
- Spatial pH profiles were deterministic benchmark trajectories and not subject-specific measurements.
- The PFR-inspired model did not reconstruct colonic geometry, axial mixing, segmental motility, individualized anatomy, or host pharmacokinetics.
- Deterministic influence ranges were conditional on the selected response, reference condition, parameter scale, and evaluated parameter ranges.
- Internal algebraic and numerical checks did not constitute experimental, biological, physiological, external, or clinical validation.
- The framework did not predict circulating IS, fecal or luminal metabolite concentrations, patient-level outcomes, treatment response, or intervention efficacy.
- The model did not predict that inhibition of IAD would produce an actual luminal IAA concentration of 1000 μM in vivo.
- At $L_{2}=0$, absence of terminal skatole-equivalent allocation represented a model-defined retained-pool upper bound rather than a prediction of actual IAA accumulation following IAD inhibition.

### 5.30. Prospective Mapping to Empirical Validation

Model outputs were prospectively mapped to three levels of future empirical validation:

- pH-controlled in vitro fermentation or defined microbial systems;
- Stable-isotope-tracing in vivo studies;
- Stratified chronic kidney disease clinical studies.

Five model-derived hypotheses addressed

- pH-controlled IAA-to-skatole conversion;
- IAD-associated terminal-conversion capacity;
- Isotope-resolved precursor flux;
- Host-dependent precursor availability;
- Stratified clinical pathway coherence.

Future microbial experiments would test whether controlled changes in pH and IAD-associated conversion capacity alter IAA-to-skatole allocation under defined conditions. Future isotope-tracing studies would distinguish precursor availability, terminal conversion, alternative metabolism, absorption, and systemic disposition. Future clinical studies would evaluate standardized fecal IAA and skatole measurements, fecal or luminal pH, transit-related variables, microbial pathway measurements, host factors, and relevant circulating metabolites. Concentrations used in those studies would require independent experimental justification and would not be selected solely from the 1000 μM technical model-scaling benchmark. If evaluated, 1000 μM IAA would represent one candidate upper-bound condition within a broader concentration–response design. Evaluation of that condition would require parallel assessment of lower concentrations, viability or microbial biomass, membrane integrity where relevant, nonspecific stress, matrix composition, exposure duration, solubility, chemical stability, vehicle effects, and relevant mechanistic endpoints. No experiment in this prospective roadmap was implemented in the present study. All hypotheses, proposed readouts, safeguards, progression criteria, and entries in Table 8 are prospective design proposals rather than procedures performed or validation data obtained.

### 5.31. Supplementary Information and Data Organization

Supplementary Figures S1–S6 and Tables S1–S8, and their associated legends and notes are provided in “Supplementary_Information.pdf”. Complete machine-readable analytical grids and figure- and table-level source data are provided in “Supplementary_Source_Data.zip”. Source paths, dimensions, column headers, and SHA-256 values are documented in the package manifest. Internal numerical-verification summaries, selected supporting records, software-environment specifications, provenance documentation, the verification inventory, package-validation records, and file-level SHA-256 manifests are provided in “Supplementary_Verification_Audit.zip”. The fixed authoritative V01–V12 summary record is “verification/verification_summary.csv”, and the verification inventory is “audit/verification_inventory.csv”. The inventory maps each verification identifier to the fixed authoritative summary, the corresponding supporting calculations or revision-stage procedural support where applicable, the relevant manuscript elements, supporting-file locations, and the role and analytical scope of each supplied record. Supporting and reconstructed records do not replace, revise, or redefine the fixed authoritative summary values. Scientific analysis code is outside the scope of the present Supplementary Materials. The supplied model equations, initial and boundary conditions, evaluated parameter grids, machine-readable analytical and source data, software-environment specifications, numerical-verification records, and provenance documentation support transparent inspection of the reported calculations and independent reimplementation from the documented equations, parameter settings, analysis conditions, and source data. The supplied materials are not presented as an executable end-to-end reproduction package.

### 5.32. Use of Generative Artificial Intelligence Tools

During manuscript preparation, Google Gemini (version 1.5 Pro) and Microsoft 365 Copilot (version GPT-5.6, as displayed in the user interface at the time of use) were used to assist with preliminary code drafting, manuscript organization, and English-language refinement. All AI-assisted outputs were critically reviewed, edited, and verified by the human authors. The generative artificial intelligence tools were not used as authors and did not determine the scientific concepts, mathematical formulation, parameter selection, interpretation, or conclusions. All mathematical formulations, parameter settings, computational procedures, numerical outputs, figures, tables, interpretations, and conclusions were independently checked and finalized by the human authors, who take full responsibility for the originality, accuracy, integrity, reproducibility, and final content of the work.

## Figures and Tables

**Table 1:** Technical benchmark concentration derived from the reference conversion assumptions. Benchmark concentrations calculated from the reference fecal skatole burden under the specified conversion assumptions. These values were used solely as technical scaling anchors for visualization and scenario construction. They do not represent measured luminal IAA concentrations, calibrated biological concentrations, diagnostic thresholds, or recommended exposure levels.

Quantity	Value (μM)
Reference benchmark	1087.4
Rounded model reference	1000.0
Uncertainty minimum	846.9
Uncertainty maximum	1753.0

Note: Values are presented as technical scaling anchors and do not represent measured biological concentrations.

**Table 2:** Representative distal terminal allocations at selected pH values. Representative normalized and absolute distal terminal allocations generated by the reference logistic formulation (${\mathrm{pH}}_{\mathrm{mid}}=6.6$, s=4) using the reference total-pool scale ($C_{\mathrm{total}}=1000\;\mu M$). Absolute distal terminal allocation scales with $C_{\mathrm{total}}$, whereas normalized allocation remains invariant to total-pool scaling.

pH	Normalized Distal Terminal Allocation	Absolute Distal Terminal Allocation (μM)
6.0	0.0832	83.2
6.4	0.3100	310.0
6.6	0.5000	500.0
6.8	0.6900	690.0
7.4	0.9608	960.8

Note: Results correspond to the reference formulation with ${\mathrm{pH}}_{\mathrm{mid}}=6.6$, s=4, and $C_{\mathrm{total}}=1000\;\mu M$.

**Table 3:** Summary sensitivity metrics of the reference logistic formulation. Summary sensitivity characteristics of the reference logistic allocation function, including maximum normalized local sensitivity, transition width, and allocation spans associated with assumed pH deviations around the reference midpoint. Reported values are conditional on the adopted reference formulation and should not be interpreted as empirical biological thresholds.

Quantity	Value	Unit
Maximum normalized sensitivity	1.0	per pH unit
10–90% transition width	1.099	pH units
Full allocation span at pH 6.6 (δ=0.10)	0.1974	normalized
Full allocation span at pH 6.6 (δ=0.20)	0.3799	normalized

**Table 4:** Distal endpoint allocation under reference pH profiles. Comparison of distal endpoint allocations under the constant pH 6.0 and linear pH 6.2–7.4 reference profiles. Table 4 summarizes only the two reference profiles used throughout the main analyses. Integrated permissiveness ($A_{\mathrm{pH}}$) summarizes cumulative modeled permissive exposure. Effective constant pH denotes the constant-pH equivalent that reproduces the same integrated permissiveness within the specified formulation. Distal endpoint allocations are reported as retained material $I\left(1\right)$, distal terminal allocation $S\left(1\right)$, and normalized distal terminal-allocation fractions.

Profile	Integrated Permissiveness (*A*pH)	Effective Constant pH	$I\left(1\right)$ (μM)	$S\left(1\right)$ (μM)	$S\left(1\right)/C_{\mathrm{total}}$
Constant pH 6.0	0.0832	6.000	717.0	283.0	0.2830
Linear pH 6.2–7.4	0.6367	6.740	78.3	921.7	0.9217

**Table 5:** Triple-Lock design structure. Structure of the deterministic Triple-Lock analysis, showing the number of evaluated reference pH profiles, $L_{2}$ levels, $L_{3}$ levels, and resulting analytical scenarios. Twenty-five $L_{2}\times L_{3}$ combinations were evaluated under each of two reference profiles, yielding 50 total scenarios. The complete parameter combinations were used to examine the separate and combined variation in the modeled locks.

Design Component	Value
Reference pH profiles	2
$L_{2}$ levels	5
$L_{3}$ levels	5
Total scenarios	50

**Table 6:** Maximum mass-balance residuals across combined relative-spatial-progression and competing-loss scenarios. Maximum absolute mass-balance residuals observed across all evaluated combinations of reference pH profile, relative spatial progression ($v_{\mathrm{rel}}$), and nominal competing-loss fraction (l). All reported residuals remained below the predefined V11 acceptance tolerance, supporting numerical mass conservation within the documented verification scope.

Reference Profile	Relative Spatial Progression ($v_{\mathrm{rel}}$)	Nominal Competing-Loss Fraction (l)	Maximum Absolute Mass-Balance Residual (μM)
Constant pH 6.0	0.5	0.00	$1.137\times 10^{-13}$
Constant pH 6.0	0.5	0.10	0
Constant pH 6.0	0.5	0.25	0
Constant pH 6.0	0.5	0.50	$2.274\times 10^{-13}$
Constant pH 6.0	1.0	0.00	$2.274\times 10^{-13}$
Constant pH 6.0	1.0	0.10	$5.684\times 10^{-13}$
Constant pH 6.0	1.0	0.25	$1.137\times 10^{-13}$
Constant pH 6.0	1.0	0.50	0
Constant pH 6.0	2.0	0.00	$3.411\times 10^{-13}$
Constant pH 6.0	2.0	0.10	$2.274\times 10^{-13}$
Constant pH 6.0	2.0	0.25	$1.137\times 10^{-13}$
Constant pH 6.0	2.0	0.50	$1.137\times 10^{-13}$
Linear pH 6.2–7.4	0.5	0.00	0
Linear pH 6.2–7.4	0.5	0.10	$7.958\times 10^{-13}$
Linear pH 6.2–7.4	0.5	0.25	$7.958\times 10^{-13}$
Linear pH 6.2–7.4	0.5	0.50	$9.095\times 10^{-13}$
Linear pH 6.2–7.4	1.0	0.00	$2.274\times 10^{-13}$
Linear pH 6.2–7.4	1.0	0.10	0
Linear pH 6.2–7.4	1.0	0.25	$1.137\times 10^{-13}$
Linear pH 6.2–7.4	1.0	0.50	0
Linear pH 6.2–7.4	2.0	0.00	$5.684\times 10^{-13}$
Linear pH 6.2–7.4	2.0	0.10	$6.821\times 10^{-13}$
Linear pH 6.2–7.4	2.0	0.25	$5.684\times 10^{-13}$
Linear pH 6.2–7.4	2.0	0.50	$1.137\times 10^{-13}$

Note: Maximum absolute mass-balance residuals remained below the predefined V11 acceptance tolerance under all evaluated conditions.

**Table 7:** Selected verification results from the fixed authoritative V01–V12 verification framework. Selected verification outcomes used to evaluate internal mathematical and numerical consistency of the analytical implementation. Displayed checks include scenario-collapse agreement, solver-tolerance stability, analytical–numerical agreement, mass-balance conservation, and state nonnegativity. All displayed checks satisfied their predefined acceptance criteria. These verification results do not constitute biological, physiological, clinical, or external validation and should be interpreted solely as evidence of internal mathematical and numerical consistency. Additional verification checks including V05 and V06 are summarized in Figure 6D and Supplementary Table S8.

Check ID	Verification Check	Scope	Criterion	Tolerance	Observed Value	Unit	Status	Observed/Tolerance Ratio
V07	Scenario-collapse maximum residual	600 ODE runs	Max residual $\leq 1\times 10^{-8}$	$1\times 10^{-8}$	$4.742\times 10^{-11}$	Dimensionless	PASS	0.004742
V08	Scenario-collapse RMSE	600 ODE runs	RMSE $\leq 1\times 10^{-9}$	$1\times 10^{-9}$	$7.086\times 10^{-12}$	Dimensionless	PASS	0.007086
V09	Solver-tolerance stability	24 runs	Max curve difference $\leq 2\times 10^{-4}$	$2\times 10^{-4}$	$1.092\times 10^{-4}$	μM	PASS	0.5460
V10	Analytical–numerical full-curve agreement	1505 locations	Max state difference $\leq 1\times 10^{-7}$	$1\times 10^{-7}$	$1.163\times 10^{-8}$	μM	PASS	0.1163
V11	Mass-balance audit	112 scenarios; 33,712 points	Max residual $\leq 1\times 10^{-7}$	$1\times 10^{-7}$	$1.364\times 10^{-12}$	μM	PASS	$1.364\times 10^{-5}$
V12	State nonnegativity	112 scenarios; 33,712 points	Negative magnitude $\leq 1\times 10^{-9}$	$1\times 10^{-9}$	0	μM	PASS	0

Note: All displayed checks satisfied their predefined acceptance criteria. These verification results support internal mathematical and numerical consistency only and do not constitute biological, physiological, external, or clinical validation.

**Table 8:** Model-derived hypotheses and prospective validation pathways. Prospective hypotheses generated from the analytical framework together with representative experimental or observational validation strategies. Suggested validation settings, primary readouts, and progression criteria are presented as future research directions only. None of the hypotheses were implemented, experimentally tested, or validated in the present study.

Hypothesis ID	Model-Derived Hypothesis	Proposed Validation Setting	Primary Readout	Progression Criterion
H1	pH-controlled IAA-to-skatole conversion	Controlled microbial fermentation system	Corrected molar conversion of available IAA to skatole	Reproducible and directionally consistent changes in conversion across independent experiments
H2	IAD-associated terminal-conversion capacity	Defined microbial system with experimentally manipulated IAD-associated capacity	Corrected molar skatole production with pathway-proximal functional measurements	Reduced terminal conversion after selective reduction in IAD-associated capacity or increased conversion after enhancement
H3	Isotope-resolved precursor flux	Stable-isotope tracing in microbial, ex vivo, or in vivo experimental systems	Flux from labeled IAA or tryptophan toward skatole and related metabolites	Reproducible isotope-resolved evidence of altered flux toward skatole under controlled perturbations
H4	Host-dependent precursor availability	Controlled animal model or host–microbial experimental system	Intestinal precursor availability together with terminal skatole formation	Directionally coherent changes in precursor delivery and terminal conversion after accounting for confounders
H5	Stratified CKD pathway coherence	Stratified observational CKD cohort	Fecal IAA, skatole, pH, microbial variables, and selected systemic measurements	Reproducible pathway-level coherence after adjustment for major intestinal and clinical covariates

Note: All hypotheses are prospective proposals. None were implemented, tested, or validated in the present study.

**Table 9:** Model parameters, reference settings, and evaluated values. Summary of the principal technical assumptions, model parameters, reference settings, and evaluated ranges used throughout the analytical framework. $L_{2}$ represents the Terminal Metabolic Lock. $L_{3}$ represents the Host-Protection Lock and is operationally defined as a dimensionless substrate-availability coefficient that scales the dynamically available precursor pool. $k_{\max}$ denotes conversion capacity, $v_{\mathrm{rel}}$ denotes relative spatial progression, and l denotes the nominal competing-loss fraction. $\Lambda_{\mathrm{conv}}$ denotes the composite conversion–progression coefficient integrating $k_{\max}$, $L_{2}$, and $v_{\mathrm{rel}}$ within the loss-free formulation. The evaluated settings were used in deterministic analyses, Triple-Lock simulations, analytical scenario-collapse assessments, response-range diagnostics, and internal-verification procedures. The listed values are technical or phenomenological model settings and were not calibrated to measured physiological quantities.

Parameter	Symbol	Reference Setting	Evaluated Values	Role and Analysis-Specific Use
Water-accessible fraction	w	0.75	0.50, 0.55, 0.60, 0.65, 0.70, 0.75, 0.80, 0.85, and 0.90	Technical conversion of the reference fecal skatole burden to an aqueous-equivalent concentration scale
Fecal density	$\rho_{\mathrm{feces}}$	$1.07{\;g\;\mathrm{mL}}^{-1}$	1.00, 1.035, 1.070, 1.110, $\;\mathrm{and}\;1.150{\;g\;\mathrm{mL}}^{-1}$	Technical benchmark-conversion analysis
Technical total-pool scale	$C_{\mathrm{total}}$	1000 μM	250, 500, 1000, 1500, and 2000 μM for total-pool scaling; 500, 1000, and 2000 μM for the analytical scenario-collapse grid	Sets the absolute output scale; normalized allocation remains invariant under fixed dimensionless parameters
Logistic midpoint	${\mathrm{pH}}_{\mathrm{mid}}$	6.6	6.2, 6.4, 6.6, and 6.8	Location of equal retained and terminal allocation in the phenomenological logistic formulation
Logistic transition steepness	s	4	2, 4, 6, and 8	Controls transition width and maximum local pH sensitivity
Maximum conversion-capacity coefficient	$k_{\max}$	4	1, 2, 4, 6, and 8	Governs modeled terminal-conversion capacity
Relative spatial-progression coefficient	$v_{\mathrm{rel}}$	1	0.5, 1, and 2	Dimensionless modifier of relative spatial progression; not a calibrated gastrointestinal transit time
Terminal Metabolic Lock coefficient	$L_{2}$	1	0, 0.25, 0.50, 0.75, and 1.00; positive values 0.25, 0.50, 0.75, and 1.00 in the analytical scenario-collapse grid	Multiplicatively restricts effective terminal-conversion capacity
Host-Protection Lock coefficient	$L_{3}$	1	0, 0.25, 0.50, 0.75, and 1.00; positive values 0.25, 0.50, and 1.00 in the analytical scenario-collapse grid	Dimensionless substrate-availability coefficient that determines the dynamically available precursor pool, $C_{\mathrm{available}}=L_{3}C_{\mathrm{total}}$
Nominal competing-loss fraction	l	0	0, 0.10, 0.25, and 0.50	Defines cumulative loss in the isolated loss-only reference process at $v_{\mathrm{rel}}=1$; realized loss may differ when loss and conversion compete
Constant pH	pH	6.0	6.0, 6.4, 6.6, and 7.4 in the reference-profile analyses; 6.0−7.5 in the deterministic one-at-a-time influence analysis	Defines spatially constant ecological permissiveness
Linear pH-profile endpoints	${\mathrm{pH}}_{\mathrm{proximal}},\;{\mathrm{pH}}_{\mathrm{distal}}$	6.2, 7.4	Proximal pH : 6.0,6.2, $\;\mathrm{and}\;6.4;\;\mathrm{distal}\;\mathrm{pH}:6.6,\;6.8,\;7.0,\;7.2,\;\mathrm{and}\;7.4,\;\mathrm{subject}\;\mathrm{to}\;{\mathrm{pH}}_{\mathrm{distal}}>{\mathrm{pH}}_{\mathrm{proximal}}$	Define monotonic linear pH profiles across normalized spatial progression

Note: The reference values were $C_{\mathrm{total}}=1000\;\mu M$, ${\mathrm{pH}}_{\mathrm{mid}}=6.6$, s=4, $k_{\max}=4$, $v_{\mathrm{rel}}=1$, $L_{2}=L_{3}=1$, and l=0, unless the parameter under examination was varied. The value $L_{3}=0$ was included as a boundary condition for absolute pool accounting but was excluded from analyses using $S\left(1\right)/C_{\mathrm{available}}$, because $C_{\mathrm{available}}=0$ makes this normalized response mathematically undefined. The values of $C_{\mathrm{total}}$, w, and $\rho_{\mathrm{feces}}$ were used as technical scaling assumptions and should not be interpreted as measured luminal IAA concentrations, calibrated physiological concentrations, diagnostic thresholds, or recommended experimental exposure levels. All remaining parameters were phenomenological or dimensionless model quantities and were not fitted to empirical data.

## Data Availability

The original contributions presented in this study are included in the article/Supplementary Materials. Further inquiries can be directed to the corresponding author.

## Supplementary Materials

The following supporting information can be downloaded at: https://www.mdpi.com/article/10.3390/toxins18090362/s1, The following supporting materials are available with the article: Supplementary Information: Supplementary Figure S1, “Detailed uncertainty in technical benchmark conversion”; Supplementary Figure S2, “Complete logistic-parameter grid and derived transition metrics”; Supplementary Figure S3, “Landscape of allocation changes under assumed pH deviations”; Supplementary Figure S4, “Linear pH-profile grid and equal-integrated-permissiveness examples”; Supplementary Figure S5, “Conversion-capacity–relative-progression response surfaces and equal-Λconv spatial equivalence”; Supplementary Figure S6, “Spatial and endpoint behavior under competing loss”; Supplementary Table S1, “Complete 45-condition technical benchmark-conversion matrix”; Supplementary Table S2, “Focused algebraic total-pool scaling and pool identity”; Supplementary Table S3A, “Complete logistic midpoint and transition-steepness grid”; Supplementary Table S3B, “Focused propagation of assumed pH deviations”; Supplementary Table S4A, “Reference constant and linear pH-profile summaries”; Supplementary Table S4B, “Nonlinear, reversed, and exposure-matched pH-profile robustness analysis”; Supplementary Table S5, “Complete L2 × L3 Triple-Lock scenario grid under the two reference pH profiles”; Supplementary Table S6, “Complete combined relative-progression and competing-loss scenario results”; Supplementary Table S7, “Deterministic one-at-a-time normalized parameter-influence summary”; and Supplementary Table S8, “Authoritative V01–V12 internal algebraic and numerical verification record”. Associated legends and notes are included in the Supplementary Information. Supplementary Source Data: Machine-readable source data underlying the main figures, main tables, supplementary figures, and supplementary tables; Supplementary Verification Audit: Authoritative internal numerical-verification summaries, selected supporting records newly generated or reconstructed during revision from the documented equations and analysis conditions, software-environment specifications, provenance documentation, verification inventory, package-validation records, and file-level SHA-256 manifests. Supplementary_Source_Data_SHA256 and Supplementary_Verification_Audit_SHA256: SHA-256 checksums for the two enclosed ZIP archives. Supplementary_Materials_SHA256: SHA-256 checksums for the six other top-level files in this package. This checksum file does not list itself, thereby avoiding self-reference.

## Abbreviations

AhR	aryl hydrocarbon receptor
CKD	chronic kidney disease
CRC	colorectal cancer
EGFR	epidermal growth factor receptor
IAA	indole-3-acetic acid
IAD	indoleacetate decarboxylase
IBD	inflammatory bowel disease
IS	indoxyl sulfate
JNK	c-Jun N-terminal kinase
MAPK	mitogen-activated protein kinase
MDR1	multidrug resistance protein 1
PBUT	protein-bound uremic toxin
PFR	plug-flow reactor
SCFA	short-chain fatty acid
TLR4	Toll-like receptor 4
