# Supplementary material for: Upstream Ecological Control of the IAA–Skatole Branch: A pH-Dependent Triple-Lock Framework for Gut-Derived Uremic Toxin Precursors
Source: Toxins (Basel). 2026 Aug 24;18(9):362. doi: 10.3390/toxins18090362 (PMC13611153; doi:10.3390/toxins18090362)
Supplement: Supplementary file 1 [file toxins-18-00362-s001.zip › Supplementary_Information_proof.pdf]

Supplementary Information

Upstream Ecological Control of the IAA–Skatole Branch: A pH-Dependent Triple-Lock Framework for Gut-Derived Uremic Toxin Precursors

This Supplementary Information provides expanded mathematical definitions, detailed scenario matrices, authoritative and supporting numerical-verification records, and documentation of the machine-readable source data supplied with the article.

Interpretive boundary: all concentrations and allocations are model-defined unless explicitly identified as reported external measurements. Internal algebraic and numerical verification establishes consistency with the stated equations and implementation; it does not constitute biological, physiological, external, or clinical validation.

S1. Scope and Organization

The main manuscript presents a reduced theoretical framework for the intestinal indole-3-acetic acid (IAA)–skatole axis. This Supplementary Information expands the mathematical formulation and reports the detailed benchmark, algebraic, spatial, Triple-Lock, competing-loss, parameter-influence, and internal-verification analyses supporting the main Results. Supplementary Tables S1–S8 provide detailed numerical summaries, whereas Supplementary Figures S1–S6 visualize the expanded parameter spaces and structural relationships. The 1000 μM reference is a sensitivity-tested technical model-scaling benchmark. It is not a measured or typical luminal IAA concentration, a physiological threshold, a therapeutic exposure, or a recommended experimental concentration. The suitability of any concentration for future experimental work must be evaluated independently for the relevant model system, exposure duration, and endpoint.

S1.1. Supplementary Item Map

Supplementary item	Main-manuscript linkage	Purpose
Supplementary Table S1; Figure S1	Main Results 2.1; Figure 1A	Technical benchmark conversion and uncertainty.
Supplementary Table S2	Main Results 2.2; Figure 1B and 1C	Algebraic total-pool scaling and pool identity.
Supplementary Table S3A; Figure S2	Main Results 2.3; Figure 2A, 2B, and 2D	Logistic midpoint/steepness grid and transition metrics.
Supplementary Table S3B; Figure S3	Main Results 2.3; Figure 2C	Focused propagation of assumed pH deviations.
Supplementary Table S4A; Figure S4	Main Results 2.4; Figure 3	Reference linear pH-profile grid and equal-integrated-permissiveness examples.
Supplementary Table S4B	Main Results 2.4; Figure 3	Broader nonlinear, reversed, and exposure-matched pH-profile robustness analysis.
Supplementary Figure S5	Main Results 2.5; Figure 4A–4C	Conversion-capacity, relative-progression, and equal-Λ_{conv} structural-equivalence analyses.
Supplementary Table S5	Main Results 2.6; Figure 5A and 5B	Complete $L_2 \times L_3$ Triple-Lock grid under two pH profiles.
Supplementary Table S6; Figure S6	Main Results 2.7; Figure 6A and 6B	Combined relative-progression and competing-loss behavior.
Supplementary Table S7	Main Results 2.8; Figure 6C	Deterministic normalized parameter-influence ranges.
Supplementary Table S8	Main Results 2.9–2.10; manuscript Table 7; Figures 4D and 6D	Authoritative V01–V12 internal-verification summary.

S2. Supplementary Mathematical Formulation

S2.1. Technical concentration benchmark

A fecal skatole value of approximately 100 μg/g fresh feces, selected as a literature-informed technical reference anchor on the basis of prior reports [S1,S2], was used only for model scaling. The transformation below defines an effective water-accessible display scale and does not equate fecal skatole burden with measured luminal IAA concentration or freely dissolved, unbound, or biologically available skatole. The 100 μg/g value was not treated as a universal physiological threshold.

$$C_{\text{benchmark}} = \frac{B_{\text{fecal}} \rho_{\text{feces}}}{MW_{\text{skatole}} w} \times 1000$$

Using $B_{\text{fecal}} = 100$ μg/g fresh feces, $\rho_{\text{feces}} = 1.07$ g/mL, $MW_{\text{skatole}} = 131.2\ \mu\text{g}\ \mu\text{mol}^{-1}$, and $w = 0.75$ yielded 1087.398 μM (1.087 mM). The rounded model reference was $C_{\text{total,ref}} = 1000\ \mu$M. The 45-condition deterministic uncertainty matrix varied w from 0.50 to 0.90 and ρ_{feces} from 1.000 to 1.150 g/mL (Supplementary Table S1; Supplementary Figure S1).

S2.2. pH-Dependent Algebraic Allocation and Local Sensitivity

A bounded logistic function represented pH-associated ecological permissiveness toward terminal allocation. Here, "terminal allocation" denotes the model-defined fraction assigned to the skatole-associated terminal branch and is not itself a measured metabolite concentration or an experimentally fitted conversion yield. This phenomenological function is not a fitted enzyme-kinetic law, a clinical cutoff, or a universal physiological threshold.

$$f_{\text{leak}}(\text{pH}) = \{1 + \exp[-s(\text{pH} - \text{pH}_{\text{mid}})]\}^{-1}$$

$$S_{\text{alg}}(\text{pH}) = C_{\text{total}} f_{\text{leak}}(\text{pH})$$

$$I_{\text{alg}}(\text{pH}) = C_{\text{total}}[1 - f_{\text{leak}}(\text{pH})]$$

By construction, $I_{\text{alg}} + S_{\text{alg}} = C_{\text{total}}$. Reference parameters were $\text{pH}_{\text{mid}} = 6.6$ and $s = 4$. Total-pool scaling was evaluated at $250, 500, 1000, 1500$, and $2000\ \mu$M. The complete grid is supplied as machine-readable source data, whereas Supplementary Table S2 provides the formatted numerical summary.

$$\frac{\partial(S_{\text{alg}}/C_{\text{total}})}{\partial \text{pH}} = s f_{\text{leak}}(\text{pH})[1 - f_{\text{leak}}(\text{pH})]$$

$$\Delta\text{pH}_{10-90} = \frac{2\ln(9)}{s}$$

The complete $\text{pH}_{\text{mid}} \times s$ grid and focused assumed-pH-deviation scenarios are reported in Supplementary Tables S3A and S3B and Supplementary Figures S2 and S3.

S2.3. Triple-Lock spatial model

The Triple-Lock framework comprised: (i) the Upstream Ecological Lock, represented by pH-dependent ecological permissiveness, $f_{\text{leak}}[\text{pH}(x)]$; (ii) the Terminal Metabolic Lock, represented by the dimensionless conversion-capacity coefficient L_2; and (iii) the Host-Protection Lock, represented operationally by the dimensionless substrate-availability coefficient L_3. L_2 modifies conversion exposure within the available pool, whereas L_3 determines the size of the dynamically available precursor pool. The Host-Protection Lock was represented operationally by $L_3 \in [0,1]$, a dimensionless substrate-availability coefficient defining the fraction of the model-defined total precursor-equivalent pool available to enter the spatial conversion system. The dynamically available and excluded pools were

$$C_{\text{available}} = L_3 C_{\text{total}}$$

and

$$C_{\text{excluded}} = (1 - L_3)C_{\text{total}},$$

respectively. L_3 therefore scales available-pool size but does not directly quantify epithelial apoptosis, epithelial permeability, epithelial turnover, mucus integrity, barrier disruption, absorption, butyrate exposure, or any individual host-protection mechanism. "Host-Protection Lock" is the conceptual label for this model layer, whereas L_3 is its operational substrate-availability coefficient. L_2 and L_3 are reduced model quantities and do not directly measure IAD abundance, enzymatic activity, epithelial integrity, permeability, mucus-barrier function, or any individual biological mechanism.

$$k_{\text{conv}}(x) = \frac{k_{\max} L_2 f_{\text{leak}}[\text{pH}(x)]}{v_{\text{rel}}}$$

$$\frac{dI}{dx} = -k_{\text{conv}}(x)I$$

$$\frac{dS}{dx} = k_{\text{conv}}(x)I$$

Initial conditions were $I(0) = C_{\text{available}}$ and $S(0) = 0$. In the loss-free model, $I(x) + S(x) = C_{\text{available}}$ and $I(x) + S(x) + C_{\text{excluded}} = C_{\text{total}}$. At $L_3 = 0$, all dynamic states are zero and $S(1)/C_{\text{available}}$ is undefined. IAA-equivalent denotes the model-tracked precursor-equivalent pool and does not imply that every retained unit is chemically unchanged IAA.

S2.4. Analytical reduction and profile integration

$$A_{\text{pH}}(x) = \int_0^x f_{\text{leak}}[\text{pH}(\xi)]\,d\xi, \quad A_{\text{pH}} = A_{\text{pH}}(1)$$

$$\Lambda_{\text{conv}} = \frac{k_{\max} L_2}{v_{\text{rel}}}, \quad \Psi = \Lambda_{\text{conv}} A_{\text{pH}}$$

$$I(x) = C_{\text{available}} \exp\left[-\Lambda_{\text{conv}} A_{\text{pH}}(x)\right]$$

$$S(x) = C_{\text{available}}\left\{1 - \exp\left[-\Lambda_{\text{conv}} A_{\text{pH}}(x)\right]\right\}$$

Thus, for $C_{\text{available}} > 0$, the loss-free distal normalized response is $S(1)/C_{\text{available}} = 1 - \exp(-\Psi)$. The exact analytical scenario collapse was evaluated across 5400 scenarios. V07 and V08 report the fixed aggregate verification results for the endpoint ODE scenario-collapse analysis. During revision, the analytical scenario-collapse grid and selected supporting calculations were generated from the documented equations, parameter settings, and analysis conditions to improve procedural transparency. These supporting calculations confirm the stated closed-form relationship and do not replace or redefine the fixed V07 and V08 aggregate verification values.

S2.5. Competing non-conversion loss

$$k_{\text{loss}} = -\ln(1 - \ell)$$

$$\frac{dI}{dx} = -\left\{\frac{k_{\max} L_2 f_{\text{leak}}[\text{pH}(x)] + k_{\text{loss}}}{v_{\text{rel}}}\right\} I$$

$$\frac{dS}{dx} = \frac{k_{\max} L_2 f_{\text{leak}}[\text{pH}(x)]}{v_{\text{rel}}} I$$

$$\frac{dQ_{\text{loss}}}{dx} = \frac{k_{\text{loss}}}{v_{\text{rel}}} I$$

The loss compartment Q_{loss} combines unmodeled non-conversion processes and does not identify their individual contributions. Because conversion and loss compete for the same precursor, realized loss need not equal the nominal isolated loss fraction. Supplementary Table S6 and Supplementary Figure S6 report the 24 combined profile–progression–loss conditions. The competing-loss compartment does not distinguish absorption, degradation, diversion into alternative metabolites, or other unmodeled processes.

S3. Scenario Design and Numerical Implementation

Deterministic analyses included 45 benchmark-conversion conditions, 16 logistic midpoint/steepness combinations, 50 Triple-Lock scenarios, 15,251 pH–L_2 response-surface conditions, 24 progression–loss conditions, and 5400 analytical scenario-collapse conditions. No random sampling, Monte Carlo simulation, statistical parameter fitting, confidence intervals, P values, or multiple-comparison procedures were used. Calculations were performed using Python 3.12.9, NumPy 1.26.4, SciPy 1.13.1, pandas 2.2.2, and Matplotlib 3.8.4. ODE calculations used the adaptive implicit Radau method. The default solver settings were rtol = 10^{-10} and atol = 10^{-12}; four tolerance levels across six representative scenarios produced 24 solver-tolerance runs. The source-data archive provides complete machine-readable grids and plotting matrices. Values shown in formatted tables are rounded for readability; machine-readable CSV files retain full computational precision.

S4. Supplementary Results Synopsis

S4.1. Benchmark, pH response, and profile effects

The technical benchmark varied from 846.883 to 1753.049 μM across the 45 deterministic density and water-accessibility conditions. Under the reference logistic parameters, normalized terminal allocation increased continuously with pH, the maximum local sensitivity occurred at pH_{mid}, and the $10-90\%$ transition width was 1.0986 pH units. Under the loss-free model, distal allocation was determined by integrated permissiveness A_{pH}; changing spatial order while preserving A_{pH} did not alter the distal endpoint. Forward and reversed profile pairs were endpoint-equivalent within numerical precision, and exposure matching produced identical distal allocation within numerical precision.

S4.2. Triple-Lock and competing-loss behavior

Positive L_3 values scaled absolute available-pool states without changing $S(1)/C_{\text{available}}$ under otherwise fixed loss-free conditions. The $L_3 = 0$ boundary yielded zero dynamic states and an undefined available-pool-normalized response. Under competing loss, absolute terminal allocation, realized loss, and the conditional terminal fraction among non-lost material depended on both relative spatial progression and the selected denominator.

S4.3. Internal numerical verification

The fixed authoritative verification summary records all 12 predefined checks as PASS. The V01–V12 summary reports aggregate verification results for algebraic identities, profile-integral bounds, endpoint equivalence, analytical–numerical agreement, scenario-collapse consistency, solver-tolerance stability, mass balance, and state nonnegativity. During revision, selected supporting records were newly generated or reconstructed from the documented equations, parameter settings, and analysis conditions to improve procedural transparency and facilitate independent inspection. These supporting records include current calculations for V03–V06, the 24-run solver-tolerance audit for V09, and the 1505-location coordinate-level procedural-support record for V10. Supporting and reconstructed records do not replace, revise, or redefine the fixed authoritative V01–V12 summary values. The V10 file identified with the suffix "_RECONSTRUCTED" provides coordinate-level procedural support generated during revision from the documented equations and analytical conditions. The suffix identifies the file as revision-stage procedural support and distinguishes its role and scope from those of the fixed aggregate V10 summary. The current 24-condition progression–loss dataset

provides a separate condition-specific mass-balance assessment for the reported competing-loss conditions. This dataset is distinct in analytical scope from the aggregate V11 verification summary and is not used to replace or redefine the fixed V11 value. The selected verification results reported in manuscript Table 7 and Figure 6D, together with the complete V01–V12 record in Supplementary Table S8 and the corresponding Figure 6D source data, use the fixed authoritative verification summary. Detailed record classifications, supporting-file locations, provenance assignments, and manuscript mappings are provided in "audit/verification_inventory.csv". All verification checks assess internal mathematical and numerical consistency only and do not constitute biological, physiological, external, or clinical validation. "Supplementary_Verification_Audit.zip" contains the fixed authoritative V01–V12 summary, the verification inventory, selected supporting records generated or reconstructed during revision, software-environment specifications, package-validation records, provenance documentation, and file-level SHA-256 manifests. The supporting records were prepared from the documented equations, parameter settings, and analysis conditions to facilitate procedural inspection and independent reimplementation. They do not replace, revise, or redefine the fixed authoritative summary values. The V10 file identified with the suffix "_RECONSTRUCTED" provides coordinate-level procedural support generated during revision. The current 24-condition progression–loss dataset is a separate condition-specific mass-balance assessment and is distinct in scope from the aggregate V11 verification summary. Scientific analysis code is not included in the Supplementary Materials.

S5. Data, Verification, and Provenance

Formatted Supplementary Figures and Tables are provided in this document. Complete machine-readable analytical grids and figure- and table-level source data are provided in "Supplementary_Source_Data.zip". Internal numerical-verification summaries, selected supporting records, software-environment specifications, provenance documentation, the verification inventory, and package-validation records are provided in "Supplementary_Verification_Audit.zip". Each ZIP archive contains its own file-level SHA-256 manifest. Scientific analysis code is not included in the present Supplementary Materials. All supplied materials are journal-hosted Supplementary files; no external public repository was used for the present submission. The Supplementary package provides the model equations, initial and boundary conditions, evaluated parameter grids, figure- and table-level source data, software and dependency versions, fixed authoritative verification summaries, selected supporting numerical records, and explicit descriptions of the scope and role of each verification record. These materials support transparent inspection of the reported calculations and independent reimplementation from the documented equations, parameter settings, analysis conditions, and source data. The fixed authoritative V01–V12 verification summary is provided in "verification/verification_summary.csv". The selected verification results reported in manuscript Table 7 and Figure 6D, the complete V01–V12 record reported in Supplementary Table S8, and the corresponding Figure 6D source data use this summary record. The file "audit/verification_inventory.csv" maps V01–V12 to the fixed authoritative summary, the available supporting calculations, the corresponding manuscript elements, and the role and scope of each supplied record. The verification inventory provides record classification and package navigation and does not replace or redefine "verification/verification_summary.csv". During revision, selected supporting records were newly generated or reconstructed from the documented equations, parameter settings, and analysis conditions to improve procedural transparency and facilitate independent inspection. These records include supporting calculations for V03–V06, the 24-run solver-tolerance assessment for V09, and the 1505-location coordinate-level procedural-support record for V10. Supporting and reconstructed records were not substituted for the fixed authoritative displayed values and do not replace, revise, or redefine the V01–V12 summary. The file "verification/reconstructed_support/V10_full_curve_analytical_numerical_verification_1505_RECONSTRUCTED.csv" provides coordinate-level procedural support reconstructed during revision from the documented equations, parameter settings, and analysis conditions. The suffix "_RECONSTRUCTED" identifies the record as revision-stage procedural support and distinguishes it from the fixed aggregate V10 summary. The reconstructed record supports inspection of the analytical–numerical comparison and does not replace, revise, or redefine the authoritative V10 value. The current 24-condition progression–loss dataset provides a separate condition-specific assessment of mass balance across the reported competing-loss conditions. This dataset is distinct in analytical scope from the fixed aggregate V11 verification summary. The condition-specific assessment and the aggregate V11 summary therefore serve different verification

purposes and are not expected to yield an identical maximum residual. The 24-condition dataset does not replace, revise, or redefine the authoritative V11 value. In this Supplementary package, “authoritative” denotes the fixed aggregate verification values used consistently in the manuscript, tables, figures, and machine-readable verification summary. “Supporting” denotes calculations supplied to facilitate inspection of specific identities, numerical comparisons, or analysis conditions. “Reconstructed” denotes procedural supporting records generated during revision from the documented mathematical formulation and analysis settings. These classifications distinguish the role and analytical scope of the supplied records.

Abbreviations

- IAA: indole-3-acetic acid
- IAD: indoleacetate decarboxylase
- MW: molecular weight
- ODE: ordinary differential equation
- RMSE: root-mean-square error

Supplementary References

S1. Zgarbová, E.; Vrzal, R. Skatole: A thin red line between its benefits and toxicity. *Biochimie* **2023**, *208*, 1–12. doi:10.1016/j.biochi.2022.12.014.

S2. Karlin, D.A.; Mastromarino, A.J.; Jones, R.D.; Stroehlein, J.R.; Lorentz, O. Fecal skatole and indole and breath methane and hydrogen in patients with large bowel polyps or cancer. *J. Cancer Res. Clin. Oncol.* **1985**, *109*, 135–141. doi:10.1007/BF00391888.

Final interpretive note

This Supplementary Information expands deterministic analyses reported in the main manuscript. No supplementary item converts model-defined allocations into validated human concentrations, clinical risks, biomarker thresholds, treatment effects, or patient-level predictions.

Supplementary Table S1. Complete 45-condition technical benchmark-conversion matrix.

Scenario	*w*	ρ_{feces} (g/mL)	Benchmark (µM)	Benchmark (mM)	Condition type
BU01	0.5	1	1524.39	1.52439	Scenario
BU02	0.55	1	1385.81	1.38581	Scenario
BU03	0.6	1	1270.33	1.27033	Scenario
BU04	0.65	1	1172.61	1.17261	Scenario
BU05	0.7	1	1088.85	1.08885	Scenario
BU06	0.75	1	1016.26	1.01626	Scenario
BU07	0.8	1	952.744	0.952744	Scenario
BU08	0.85	1	896.7	0.8967	Scenario
BU09	0.9	1	846.883	0.846883	Scenario
BU10	0.5	1.035	1577.74	1.57774	Scenario
BU11	0.55	1.035	1434.31	1.43431	Scenario
BU12	0.6	1.035	1314.79	1.31479	Scenario
BU13	0.65	1.035	1213.65	1.21365	Scenario
BU14	0.7	1.035	1126.96	1.12696	Scenario
BU15	0.75	1.035	1051.83	1.05183	Scenario
BU16	0.8	1.035	986.09	0.98609	Scenario
BU17	0.85	1.035	928.085	0.928085	Scenario
BU18	0.9	1.035	876.524	0.876524	Scenario
BU19	0.5	1.07	1631.1	1.6311	Scenario
BU20	0.55	1.07	1482.82	1.48282	Scenario
BU21	0.6	1.07	1359.25	1.35925	Scenario
BU22	0.65	1.07	1254.69	1.25469	Scenario
BU23	0.7	1.07	1165.07	1.16507	Scenario
BU24	0.75	1.07	1087.4	1.0874	Reference
BU25	0.8	1.07	1019.44	1.01944	Scenario
BU26	0.85	1.07	959.469	0.959469	Scenario
BU27	0.9	1.07	906.165	0.906165	Scenario
BU28	0.5	1.11	1692.07	1.69207	Scenario
BU29	0.55	1.11	1538.25	1.53825	Scenario
BU30	0.6	1.11	1410.06	1.41006	Scenario
BU31	0.65	1.11	1301.59	1.30159	Scenario
BU32	0.7	1.11	1208.62	1.20862	Scenario
BU33	0.75	1.11	1128.05	1.12805	Scenario
BU34	0.8	1.11	1057.55	1.05755	Scenario
BU35	0.85	1.11	995.337	0.995337	Scenario
BU36	0.9	1.11	940.041	0.940041	Scenario
BU37	0.5	1.15	1753.05	1.75305	Scenario
BU38	0.55	1.15	1593.68	1.59368	Scenario
BU39	0.6	1.15	1460.87	1.46087	Scenario
BU40	0.65	1.15	1348.5	1.3485	Scenario
BU41	0.7	1.15	1252.18	1.25218	Scenario
BU42	0.75	1.15	1168.7	1.1687	Scenario
BU43	0.8	1.15	1095.66	1.09566	Scenario
BU44	0.85	1.15	1031.21	1.03121	Scenario
BU45	0.9	1.15	973.916	0.973916	Scenario

Note: The benchmark is a technical model-scaling quantity and not a measured luminal IAA concentration.

Supplementary Table S2. Focused algebraic total-pool scaling and pool identity.

pH	C_{total} (μM)	S_{alg}/C_{total}	S_{alg} (µM)	I_{alg} (µM)	Pool residual (µM)
5	250	0.0016588	0.4147	249.585	0
5.5	250	0.0121284	3.03211	246.968	2.842×10^{-14}
6	250	0.0831727	20.7932	229.207	0
6.4	250	0.310026	77.5064	172.494	0
6.6	250	0.5	125	125	0
6.8	250	0.689974	172.494	77.5064	0
7	250	0.832018	208.005	41.9954	0
7.4	250	0.960834	240.209	9.79143	0
8	250	0.996316	249.079	0.92106	0
8.5	250	0.9995	249.875	0.12505	2.842×10^{-14}
5	500	0.0016588	0.829401	499.171	0
5.5	500	0.0121284	6.06422	493.936	5.684×10^{-14}
6	500	0.0831727	41.5863	458.414	0
6.4	500	0.310026	155.013	344.987	0
6.6	500	0.5	250	250	0
6.8	500	0.689974	344.987	155.013	0
7	500	0.832018	416.009	83.9908	0
7.4	500	0.960834	480.417	19.5829	0
8	500	0.996316	498.158	1.84212	0
8.5	500	0.9995	499.75	0.250101	5.684×10^{-14}
5	1000	0.0016588	1.6588	998.341	0
5.5	1000	0.0121284	12.1284	987.872	1.137×10^{-13}
6	1000	0.0831727	83.1727	916.827	0
6.4	1000	0.310026	310.026	689.974	0
6.6	1000	0.5	500	500	0
6.8	1000	0.689974	689.974	310.026	0
7	1000	0.832018	832.018	167.982	0
7.4	1000	0.960834	960.834	39.1657	0
8	1000	0.996316	996.316	3.68424	1.137×10^{-13}
8.5	1000	0.9995	999.5	0.500201	1.137×10^{-13}
5	1500	0.0016588	2.4882	1497.51	0
5.5	1500	0.0121284	18.1927	1481.81	2.274×10^{-13}
6	1500	0.0831727	124.759	1375.24	2.274×10^{-13}
6.4	1500	0.310026	465.038	1034.96	0
6.6	1500	0.5	750	750	0
6.8	1500	0.689974	1034.96	465.038	0
7	1500	0.832018	1248.03	251.972	0
7.4	1500	0.960834	1441.25	58.7486	0
8	1500	0.996316	1494.47	5.52636	0
8.5	1500	0.9995	1499.25	0.750302	0
5	2000	0.0016588	3.3176	1996.68	2.274×10^{-13}
5.5	2000	0.0121284	24.2569	1975.74	0
6	2000	0.0831727	166.345	1833.65	0
6.4	2000	0.310026	620.051	1379.95	0
6.6	2000	0.5	1000	1000	0
6.8	2000	0.689974	1379.95	620.051	0
7	2000	0.832018	1664.04	335.963	0
7.4	2000	0.960834	1921.67	78.3314	0
8	2000	0.996316	1992.63	7.36848	0
8.5	2000	0.9995	1999	1.0004	0

Note: The complete 1755-row algebraic grid is provided in the machine-readable source-data archive.

Supplementary Table S3A. Complete logistic midpoint and transition-steepness grid.

pH_{mid}	*s*	pH_10	pH_25	pH_50	pH_75	pH_90	ΔpH_{10-90}	Max sensitivity	Alloc. pH 6.4	Alloc. pH 6.6	Alloc. pH 6.8
6.2	2	5.10139	5.65069	6.2	6.74931	7.29861	2.19722	0.5	0.598688	0.689974	0.768525
6.2	4	5.65069	5.92535	6.2	6.47465	6.74931	1.09861	1	0.689974	0.832018	0.916827
6.2	6	5.8338	6.0169	6.2	6.3831	6.5662	0.732408	1.5	0.768525	0.916827	0.973403
6.2	8	5.92535	6.06267	6.2	6.33733	6.47465	0.549306	2	0.832018	0.960834	0.991837
6.4	2	5.30139	5.85069	6.4	6.94931	7.49861	2.19722	0.5	0.5	0.598688	0.689974
6.4	4	5.85069	6.12535	6.4	6.67465	6.94931	1.09861	1	0.5	0.689974	0.832018
6.4	6	6.0338	6.2169	6.4	6.5831	6.7662	0.732408	1.5	0.5	0.768525	0.916827
6.4	8	6.12535	6.26267	6.4	6.53733	6.67465	0.549306	2	0.5	0.832018	0.960834
6.6	2	5.50139	6.05069	6.6	7.14931	7.69861	2.19722	0.5	0.401312	0.5	0.598688
6.6	4	6.05069	6.32535	6.6	6.87465	7.14931	1.09861	1	0.310026	0.5	0.689974
6.6	6	6.2338	6.4169	6.6	6.7831	6.9662	0.732408	1.5	0.231475	0.5	0.768525
6.6	8	6.32535	6.46267	6.6	6.73733	6.87465	0.549306	2	0.167982	0.5	0.832018
6.8	2	5.70139	6.25069	6.8	7.34931	7.89861	2.19722	0.5	0.310026	0.401312	0.5
6.8	4	6.25069	6.52535	6.8	7.07465	7.34931	1.09861	1	0.167982	0.310026	0.5
6.8	6	6.4338	6.6169	6.8	6.9831	7.1662	0.732408	1.5	0.0831727	0.231475	0.5
6.8	8	6.52535	6.66267	6.8	6.93733	7.07465	0.549306	2	0.0391657	0.167982	0.5

Supplementary Table S3B. Focused propagation of assumed pH deviations.

Nominal pH	δ	Lower pH	Upper pH	Lower alloc.	Nominal alloc.	Upper alloc.	Full interval	Full interval (μM)
6.4	0.1	6.3	6.5	0.231475	0.310026	0.401312	0.169837	169.837
6.45	0.1	6.35	6.55	0.268941	0.354344	0.450166	0.181225	181.225
6.5	0.1	6.4	6.6	0.310026	0.401312	0.5	0.189974	189.974
6.55	0.1	6.45	6.65	0.354344	0.450166	0.549834	0.19549	195.49
6.6	0.1	6.5	6.7	0.401312	0.5	0.598688	0.197375	197.375
6.65	0.1	6.55	6.75	0.450166	0.549834	0.645656	0.19549	195.49
6.7	0.1	6.6	6.8	0.5	0.598688	0.689974	0.189974	189.974
6.75	0.1	6.65	6.85	0.549834	0.645656	0.731059	0.181225	181.225
6.8	0.1	6.7	6.9	0.598688	0.689974	0.768525	0.169837	169.837
6.4	0.2	6.2	6.6	0.167982	0.310026	0.5	0.332018	332.018
6.45	0.2	6.25	6.65	0.197816	0.354344	0.549834	0.352018	352.018
6.5	0.2	6.3	6.7	0.231475	0.401312	0.598688	0.367212	367.212
6.55	0.2	6.35	6.75	0.268941	0.450166	0.645656	0.376715	376.715
6.6	0.2	6.4	6.8	0.310026	0.5	0.689974	0.379949	379.949
6.65	0.2	6.45	6.85	0.354344	0.549834	0.731059	0.376715	376.715
6.7	0.2	6.5	6.9	0.401312	0.598688	0.768525	0.367212	367.212
6.75	0.2	6.55	6.95	0.450166	0.645656	0.802184	0.352018	352.018
6.8	0.2	6.6	7	0.5	0.689974	0.832018	0.332018	332.018

Supplementary Table S4A. Reference constant and linear pH-profile summaries.

Profile	A_{pH}	Effective pH	$I(1)$ (μM)	$S(1)$ (μM)	$S(1)/C_{\mathrm{total}}$	Profile role
Constant pH 6.0	0.0831727	6	716.992	283.008	0.283008	Constant-profile reference
Constant pH 6.4	0.310026	6.4	289.355	710.645	0.710645	Constant-profile reference
Constant pH 6.6	0.5	6.6	135.335	864.665	0.864665	Constant-profile reference
Constant pH 7.4	0.960834	7.4	21.422	978.578	0.978578	Constant-profile reference
Linear pH 6.2–7.4	0.636678	6.74024	78.3389	921.661	0.921661	Linear-profile reference

Supplementary Table S4B. Nonlinear, reversed, and exposure-matched pH-profile robustness analysis.

Profile	Variant	A_{pH}	Linear ref. A_{pH}	pH offset	Effective pH	Proximal pH	Distal pH	$I(1)$ (μM)	$S(1)$ (μM)	$S(1)/C_{total}$
Early-rise square-root	Forward	0.782962	0.636678	0	6.92075	6.2	7.4	43.637	956.363	0.956363
Early-rise square-root	Reversed	0.782962	0.636678	0	6.92075	7.4	6.2	43.637	956.363	0.956363
Early-rise square-root	Exposure-matched	0.636678	0.636678	-0.231394	6.74024	5.96861	7.16861	78.3389	921.661	0.921661
Late-rise quadratic	Forward	0.480733	0.636678	0	6.58072	6.2	7.4	146.178	853.822	0.853822
Late-rise quadratic	Reversed	0.480733	0.636678	0	6.58072	7.4	6.2	146.178	853.822	0.853822
Late-rise quadratic	Exposure-matched	0.636678	0.636678	0.220518	6.74024	6.42052	7.62052	78.3389	921.661	0.921661
Normalized symmetric sigmoid	Forward	0.609442	0.636678	0	6.71124	6.2	7.4	87.3558	912.644	0.912644
Normalized symmetric sigmoid	Reversed	0.609442	0.636678	0	6.71124	7.4	6.2	87.3558	912.644	0.912644
Normalized symmetric sigmoid	Exposure-matched	0.636678	0.636678	0.0495721	6.74024	6.24957	7.44957	78.3389	921.661	0.921661
Cubic smoothstep	Forward	0.618533	0.636678	0	6.72083	6.2	7.4	84.2362	915.764	0.915764
Cubic smoothstep	Reversed	0.618533	0.636678	0	6.72083	7.4	6.2	84.2362	915.764	0.915764
Cubic smoothstep	Exposure-matched	0.636678	0.636678	0.0310591	6.74024	6.23106	7.43106	78.3389	921.661	0.921661
Proximal plateau–distal rise	Forward	0.496069	0.636678	0	6.59607	6.2	7.4	137.48	862.52	0.86252
Proximal plateau–distal rise	Reversed	0.496069	0.636678	0	6.59607	7.4	6.2	137.48	862.52	0.86252
Proximal plateau–distal rise	Exposure-matched	0.636678	0.636678	0.218225	6.74024	6.41822	7.61822	78.3389	921.661	0.921661
Early rise–distal plateau	Forward	0.733925	0.636678	0	6.85366	6.2	7.4	53.0936	946.906	0.946906
Early rise–distal plateau	Reversed	0.733925	0.636678	0	6.85366	7.4	6.2	53.0936	946.906	0.946906
Early rise–distal plateau	Exposure-matched	0.636678	0.636678	-0.179764	6.74024	6.02024	7.22024	78.3389	921.661	0.921661
Mildly undulating	Forward	0.655622	0.636678	0	6.76096	6.2	7.4	72.6219	927.378	0.927378
Mildly undulating	Reversed	0.655622	0.636678	0	6.76096	7.4	6.2	72.6219	927.378	0.927378
Mildly undulating	Exposure-matched	0.636678	0.636678	-0.0256829	6.74024	6.17432	7.37432	78.3389	921.661	0.921661
Sinusoidal ease	Forward	0.616845	0.636678	0	6.71904	6.2	7.4	84.8068	915.193	0.915193
Sinusoidal ease	Reversed	0.616845	0.636678	0	6.71904	7.4	6.2	84.8068	915.193	0.915193
Sinusoidal ease	Exposure-matched	0.636678	0.636678	0.0343476	6.74024	6.23435	7.43435	78.3389	921.661	0.921661

Note: Eight nonlinear profile families were examined. The sinusoidal-ease profile was included in this broader robustness analysis but excluded from the 5400-scenario collapse grid. Forward–reversed pairs and exposure-matched variants were endpoint-equivalent within numerical precision. Exposure-matched variants were generated by applying a profile-specific constant pH offset and were not constrained to retain the original $6.2 - 7.4$ endpoint range.

Supplementary Table S5. Complete $L_2 \times L_3$ Triple-Lock scenario grid under the two reference pH profiles.

Scenario	Profile	L_2	L_3	$C_{\text{available}}$ (μM)	Ψ	$I(1)$ (μM)	$S(1)$ (μM)	$S(1)/C_{\text{available}}$	C_{excluded} (μM)
TL01	Constant pH 6.0	0	0	0	0	0	0	—	1000
TL02	Constant pH 6.0	0	0.25	250	0	250	0	0	750
TL03	Constant pH 6.0	0	0.5	500	0	500	0	0	500
TL04	Constant pH 6.0	0	0.75	750	0	750	0	0	250
TL05	Constant pH 6.0	0	1	1000	0	1000	0	0	0
TL06	Constant pH 6.0	0.25	0	0	0.0831727	0	0	—	1000
TL07	Constant pH 6.0	0.25	0.25	250	0.0831727	230.048	19.9519	0.0798078	750
TL08	Constant pH 6.0	0.25	0.5	500	0.0831727	460.096	39.9039	0.0798078	500
TL09	Constant pH 6.0	0.25	0.75	750	0.0831727	690.144	59.8558	0.0798078	250
TL10	Constant pH 6.0	0.25	1	1000	0.0831727	920.192	79.8078	0.0798078	0
TL11	Constant pH 6.0	0.5	0	0	0.166345	0	0	—	1000
TL12	Constant pH 6.0	0.5	0.25	250	0.166345	211.688	38.3116	0.153246	750
TL13	Constant pH 6.0	0.5	0.5	500	0.166345	423.377	76.6231	0.153246	500
TL14	Constant pH 6.0	0.5	0.75	750	0.166345	635.065	114.935	0.153246	250
TL15	Constant pH 6.0	0.5	1	1000	0.166345	846.754	153.246	0.153246	0
TL16	Constant pH 6.0	0.75	0	0	0.249518	0	0	—	1000
TL17	Constant pH 6.0	0.75	0.25	250	0.249518	194.794	55.206	0.220824	750
TL18	Constant pH 6.0	0.75	0.5	500	0.249518	389.588	110.412	0.220824	500
TL19	Constant pH 6.0	0.75	0.75	750	0.249518	584.382	165.618	0.220824	250
TL20	Constant pH 6.0	0.75	1	1000	0.249518	779.176	220.824	0.220824	0
TL21	Constant pH 6.0	1	0	0	0.332691	0	0	—	1000
TL22	Constant pH 6.0	1	0.25	250	0.332691	179.248	70.752	0.283008	750
TL23	Constant pH 6.0	1	0.5	500	0.332691	358.496	141.504	0.283008	500
TL24	Constant pH 6.0	1	0.75	750	0.332691	537.744	212.256	0.283008	250
TL25	Constant pH 6.0	1	1	1000	0.332691	716.992	283.008	0.283008	0
TL26	Linear pH 6.2 – 7.4	0	0	0	0	0	0	—	1000
TL27	Linear pH 6.2 – 7.4	0	0.25	250	0	250	0	0	750
TL28	Linear pH 6.2 – 7.4	0	0.5	500	0	500	0	0	500
TL29	Linear pH 6.2 – 7.4	0	0.75	750	0	750	0	0	250
TL30	Linear pH 6.2 – 7.4	0	1	1000	0	1000	0	0	0
TL31	Linear pH 6.2 – 7.4	0.25	0	0	0.636678	0	0	—	1000
TL32	Linear pH 6.2 – 7.4	0.25	0.25	250	0.636678	132.262	117.738	0.470953	750
TL33	Linear pH 6.2 – 7.4	0.25	0.5	500	0.636678	264.524	235.476	0.470953	500
TL34	Linear pH 6.2 – 7.4	0.25	0.75	750	0.636678	396.785	353.215	0.470953	250

TL35	Linear<br>pH 6.2 – 7.4	0.25	1	1000	0.636678	529.047	470.953	0.470953	0
TL36	Linear<br>pH 6.2 – 7.4	0.5	0	0	1.27336	0	0	—	1000
TL37	Linear<br>pH 6.2 – 7.4	0.5	0.25	250	1.27336	69.9727	180.027	0.720109	750
TL38	Linear<br>pH 6.2 – 7.4	0.5	0.5	500	1.27336	139.945	360.055	0.720109	500
TL39	Linear<br>pH 6.2 – 7.4	0.5	0.75	750	1.27336	209.918	540.082	0.720109	250
TL40	Linear<br>pH 6.2 – 7.4	0.5	1	1000	1.27336	279.891	720.109	0.720109	0
TL41	Linear<br>pH 6.2 – 7.4	0.75	0	0	1.91003	0	0	—	1000
TL42	Linear<br>pH 6.2 – 7.4	0.75	0.25	250	1.91003	37.0189	212.981	0.851924	750
TL43	Linear<br>pH 6.2 – 7.4	0.75	0.5	500	1.91003	74.0378	425.962	0.851924	500
TL44	Linear<br>pH 6.2 – 7.4	0.75	0.75	750	1.91003	111.057	638.943	0.851924	250
TL45	Linear<br>pH 6.2 – 7.4	0.75	1	1000	1.91003	148.076	851.924	0.851924	0
TL46	Linear<br>pH 6.2 – 7.4	1	0	0	2.54671	0	0	—	1000
TL47	Linear<br>pH 6.2 – 7.4	1	0.25	250	2.54671	19.5847	230.415	0.921661	750
TL48	Linear<br>pH 6.2 – 7.4	1	0.5	500	2.54671	39.1695	460.831	0.921661	500
TL49	Linear<br>pH 6.2 – 7.4	1	0.75	750	2.54671	58.7542	691.246	0.921661	250
TL50	Linear<br>pH 6.2 – 7.4	1	1	1000	2.54671	78.3389	921.661	0.921661	0

Note: L_2 denotes the Terminal Metabolic Lock coefficient and modifies terminal-conversion exposure within the dynamically available precursor pool. L_3 denotes the Host-Protection Lock coefficient and is operationally defined as a dimensionless substrate-availability coefficient that determines $C_{\text{available}} = L_3 C_{\text{total}}$. When $L_3 = 0$, both $C_{\text{available}}$ and $S(1)$ equal zero; consequently, $S(1)/C_{\text{available}}$ is mathematically undefined and is represented by a blank cell rather than zero. For positive L_3, normalization by $C_{\text{available}}$ removes the direct multiplicative effect of L_3. The reported scenarios are deterministic model outputs and do not establish biological synergy, pharmacological synergy, statistical interaction, effect modification, or causal interaction.

Supplementary Table S6. Complete combined relative-progression and competing-loss scenario results.

Profile	v_{rel}	ℓ	$I(1)$ (μM)	$S(1)$ (μM)	$Q_{loss}(1)$ (μM)	I/C_{total}	S/C_{total}	Q_{loss}/C_{total}	$S/[I+S]$	Max balance error (μM)
Constant pH 6.0	0.5	0	514.077	485.923	0	0.514077	0.485923	0	0.485923	4.547×10^{-13}
Constant pH 6.0	0.5	0.1	416.403	443.23	140.367	0.416403	0.44323	0.140367	0.515604	1.137×10^{-12}
Constant pH 6.0	0.5	0.25	289.168	381.202	329.63	0.289168	0.381202	0.32963	0.568643	7.958×10^{-13}
Constant pH 6.0	0.5	0.5	128.519	282.631	588.85	0.128519	0.282631	0.58885	0.687415	9.095×10^{-13}
Constant pH 6.0	1	0	716.992	283.008	0	0.716992	0.283008	0	0.283008	4.547×10^{-13}
Constant pH 6.0	1	0.1	645.293	269.393	85.3145	0.645293	0.269393	0.0853145	0.29452	9.095×10^{-13}
Constant pH 6.0	1	0.25	537.744	247.897	214.359	0.537744	0.247897	0.214359	0.315534	7.958×10^{-13}
Constant pH 6.0	1	0.5	358.496	208.047	433.457	0.358496	0.208047	0.433457	0.367222	4.547×10^{-13}
Constant pH 6.0	2	0	846.754	153.246	0	0.846754	0.153246	0	0.153246	4.547×10^{-13}
Constant pH 6.0	2	0.1	803.301	149.389	47.3102	0.803301	0.149389	0.0473102	0.156807	4.547×10^{-13}
Constant pH 6.0	2	0.25	733.31	143.019	123.671	0.73331	0.143019	0.123671	0.163203	4.547×10^{-13}
Constant pH 6.0	2	0.5	598.745	130.131	271.123	0.598745	0.130131	0.271123	0.178537	2.274×10^{-13}
Linear pH 6.2 – 7.4	0.5	0	6.13699	993.863	0	0.00613699	0.993863	0	0.993863	6.821×10^{-13}
Linear pH 6.2 – 7.4	0.5	0.1	4.97096	930.438	64.5909	0.00497096	0.930438	0.0645909	0.994686	4.547×10^{-13}
Linear pH 6.2 – 7.4	0.5	0.25	3.45206	833.613	162.935	0.00345206	0.833613	0.162935	0.995876	7.958×10^{-13}
Linear pH 6.2 – 7.4	0.5	0.5	1.53425	664.741	333.725	0.00153425	0.664741	0.333725	0.997697	6.821×10^{-13}
Linear pH 6.2 – 7.4	1	0	78.3389	921.661	0	0.0783389	0.921661	0	0.921661	6.821×10^{-13}
Linear pH 6.2 – 7.4	1	0.1	70.505	880.372	49.1231	0.070505	0.880372	0.0491231	0.925853	6.821×10^{-13}
Linear pH 6.2 – 7.4	1	0.25	58.7542	814.534	126.712	0.0587542	0.814534	0.126712	0.932721	4.547×10^{-13}
Linear pH 6.2 – 7.4	1	0.5	39.1695	690.096	270.735	0.0391695	0.690096	0.270735	0.946289	7.958×10^{-13}
Linear pH 6.2 – 7.4	2	0	279.891	720.109	0	0.279891	0.720109	0	0.720109	6.821×10^{-13}
Linear pH 6.2 – 7.4	2	0.1	265.528	700.633	33.8393	0.265528	0.700633	0.0338393	0.725172	6.821×10^{-13}
Linear pH 6.2 – 7.4	2	0.25	242.393	668.461	89.1462	0.242393	0.668461	0.0891462	0.733884	7.958×10^{-13}
Linear pH 6.2 – 7.4	2	0.5	197.913	603.32	198.767	0.197913	0.60332	0.198767	0.75299	6.821×10^{-13}

Note: Nominal ℓ defines isolated cumulative loss at $v_{rel} = 1$. Realized loss differs when conversion is active because conversion and loss compete for the same precursor pool.

Supplementary Table S7. Deterministic one-at-a-time normalized parameter-influence summary.

Rank	Parameter	Influence range, R_j	Reference-scaled influence
1	L_2	0.921661	1
2	Constant pH	0.69662	0.755831
3	k_{max}	0.52291	0.567356
4	v_{rel}	0.273754	0.297022
5	Nominal loss fraction, ℓ	0.231565	0.251248
6	Distal pH	0.201619	0.218756
7	pH_{mid}	0.102923	0.111671
8	Proximal pH	0.0484008	0.0525147
9	Transition steepness, s	0.0243241	0.0263916
10	C_{total}	0	0
11	L_3	0	0

Note: The selected response was $S(1)/C_{\text{available}}$. Influence ranges were deterministic and conditional on the evaluated parameter ranges and reference condition. C_{total} and positive L_3 values had zero influence on this normalized response. The undefined $L_3 = 0$ boundary was excluded. These values are not variance-based global sensitivity indices, causal effects, or universal biological rankings. L_2, Terminal Metabolic Lock coefficient; L_3, available-pool fraction used operationally for the Host-Protection Lock; ℓ, nominal isolated competing-loss fraction; v_{rel}, relative spatial-progression coefficient.

Supplementary Table S8. Authoritative V01–V12 internal algebraic and numerical verification record.

ID	Verification	Scope	Criterion	Tolerance	Observed	Unit	Observed/ tolerance	Status
V01	Algebraic pool identity	Complete algebraic grid	Maximum absolute residual	1.000×10^{-12}	0	µM	0	PASS
V02	Normalized total-pool invariance	Complete grid	Maximum absolute difference	1.000×10^{-12}	0	Dimensionless	0	PASS
V03	Logistic-parameter identities	80 tests	Maximum absolute error	1.000×10^{-12}	0	Dimensionless	0	PASS
V04	Profile-integral bounds	10 profiles	Maximum bound violation	1.000×10^{-12}	0	Dimensionless	0	PASS
V05	Profile reversal equivalence	10 profiles	Maximum endpoint difference	1.000×10^{-8}	0	µM	0	PASS
V06	Joint pH–L_2 analytical–numerical agreement	70 conditions	Maximum absolute difference	1.000×10^{-10}	5.329×10^{-15}	Dimensionless	5.329×10^{-5}	PASS
V07	Scenario-collapse maximum residual	600 endpoint ODE runs; fixed aggregate summary	Maximum absolute residual	1.000×10^{-8}	4.742×10^{-11}	Dimensionless	0.00474192	PASS
V08	Scenario-collapse RMSE	600 endpoint ODE runs; fixed aggregate summary	RMSE	1.000×10^{-9}	7.086×10^{-12}	Dimensionless	0.0070856	PASS
V09	Solver-tolerance stability	24 runs	Maximum full-curve difference	2.000×10^{-4}	1.092×10^{-4}	µM	0.545992	PASS
V10	Analytical–numerical full-curve agreement	1505 locations; procedural support reconstructed during revision	Maximum state difference	1.000×10^{-7}	1.163×10^{-8}	µM	0.11633	PASS
V11	Mass-balance audit	112 scenarios; 33,712 points; fixed aggregate summary	Maximum absolute residual	1.000×10^{-7}	1.364×10^{-12}	µM	1.364×10^{-5}	PASS
V12	State nonnegativity	112 scenarios; 33,712 points; fixed aggregate summary	Most negative magnitude	1.000×10^{-9}	0	µM	0	PASS

Note: Observed-to-tolerance ratios were calculated only for checks with strictly positive numerical tolerances. Detailed record classifications, supporting-file locations, provenance assignments, and manuscript mappings for V01–V12 are provided in audit/verification_inventory.csv. V01–V12 are fixed authoritative aggregate verification summaries. During revision, selected supporting records were newly generated or reconstructed from the documented equations, parameter settings, and analysis conditions to improve procedural transparency. The V10 "_RECONSTRUCTED" file provides coordinate-level procedural support generated during revision. The current 24-condition progression–loss dataset provides a separate condition-specific mass-balance assessment and is distinct in scope from the aggregate V11 summary. Supporting and reconstructed calculations do not replace, revise, or redefine the fixed authoritative displayed values. All checks assess internal mathematical and numerical consistency only and do not constitute biological, physiological, external, or clinical validation.

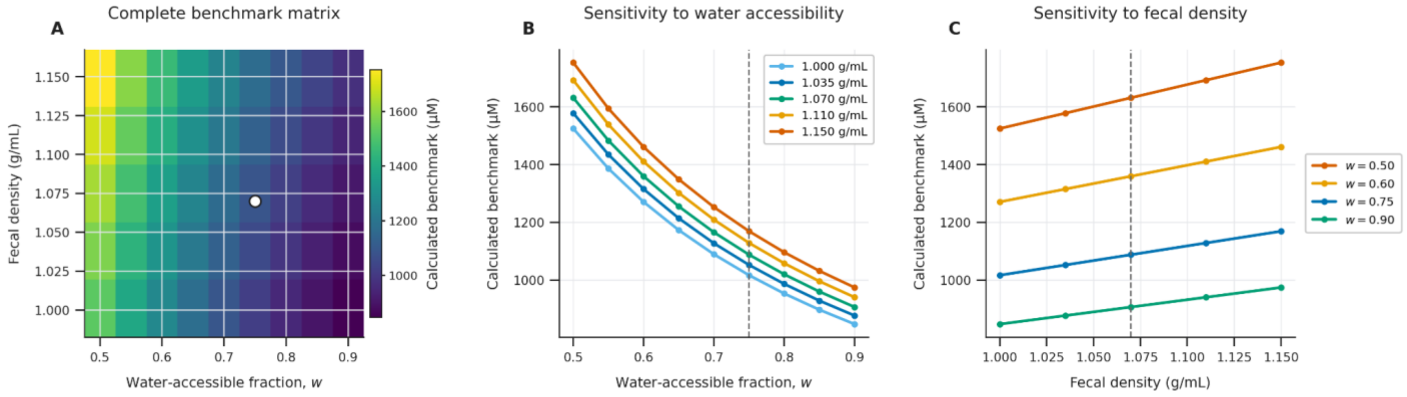

Supplementary Figure S1. Detailed uncertainty in technical benchmark conversion. (A) Complete 45-condition benchmark matrix across water-accessible fractions $w = 0.50 - 0.90$ and fecal densities $\rho_{\text{feces}} = 1.000 - 1.150$ g/mL; the white marker identifies the reference condition. (B) Benchmark variation with water-accessible fraction at each density. (C) Benchmark variation with fecal density at representative water-accessible fractions. The analysis characterizes uncertainty in a technical display scale and does not estimate measured luminal indole-3-acetic acid (IAA). The underlying fecal skatole reference anchor and its literature basis are described in Section S2.1 and Supplementary References S1 and S2.

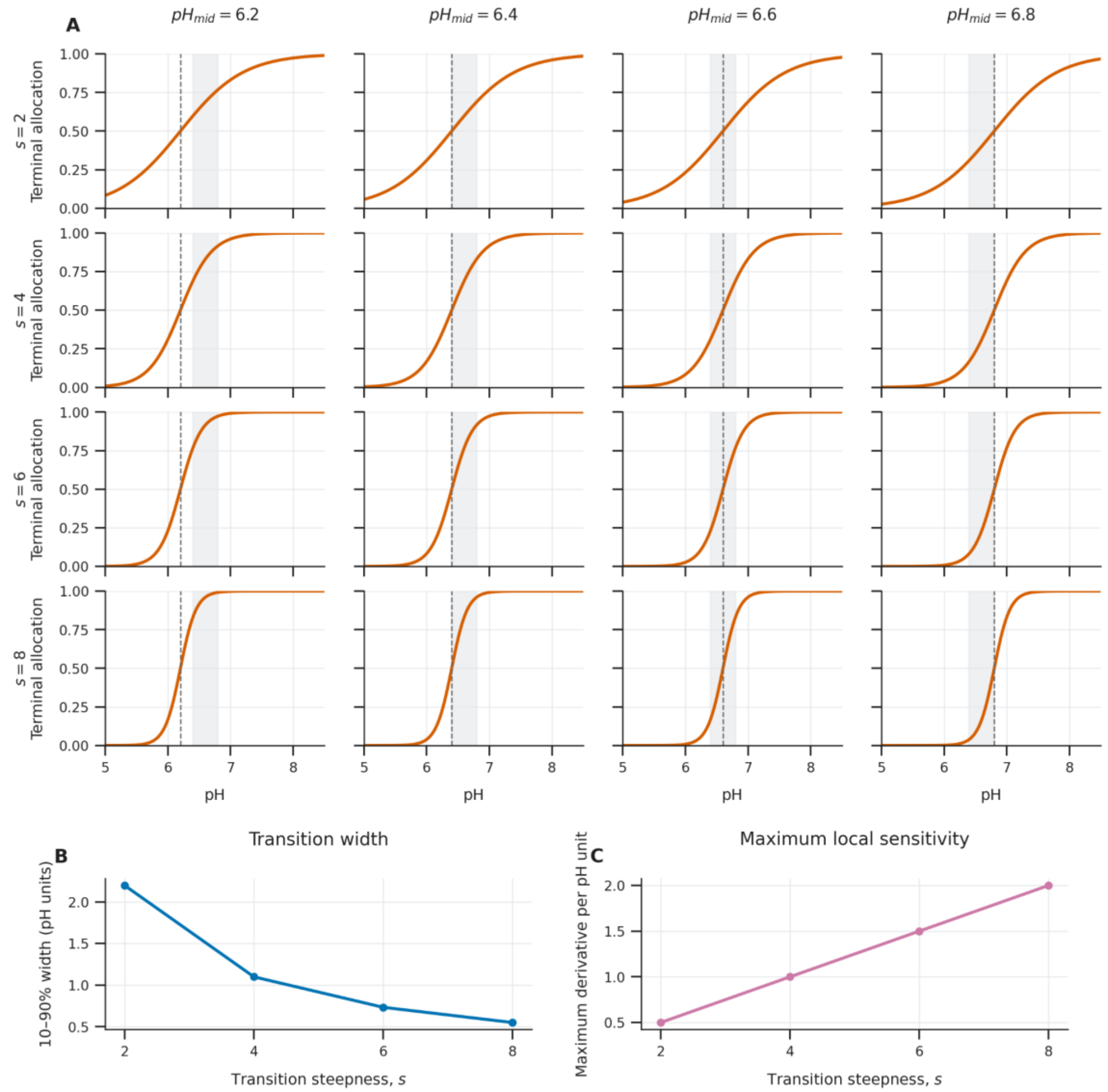

Supplementary Figure S2. Complete logistic-parameter grid and derived transition metrics. (A) Terminal-allocation curves for all 16 combinations of $pH_{mid} = 6.2, 6.4, 6.6$, or 6.8 and $s = 2, 4, 6$, or 8. (B) The $10 - 90\%$ transition width decreased as s increased. (C) Maximum local sensitivity increased as $s/4$. Shading denotes the focused pH interval used in the main analysis.

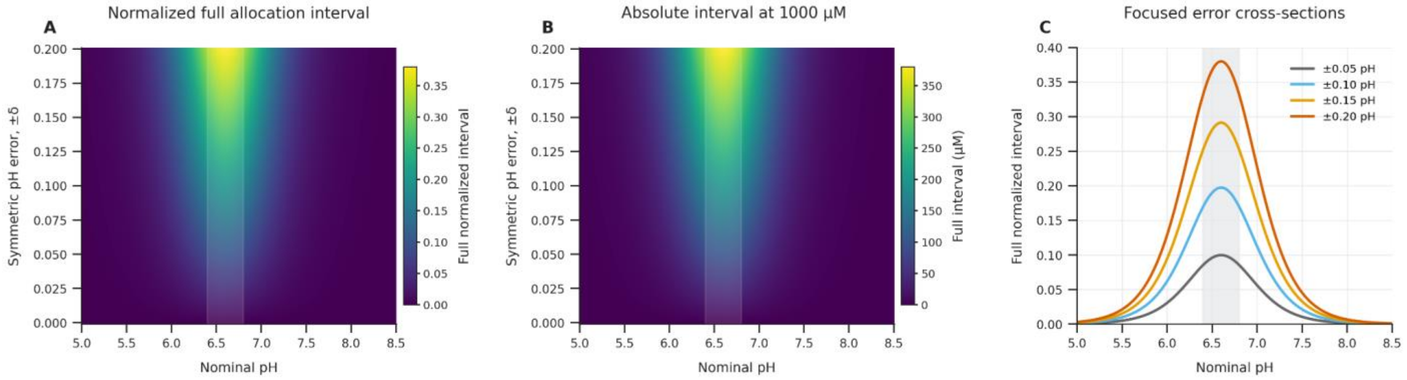

Supplementary Figure S3. Landscape of allocation changes under assumed pH deviations. (A) Full normalized allocation interval across nominal pH and symmetric perturbation δ. (B) Corresponding absolute interval at $C_{\text{total}} = 1000\ \mu$M. (C) Focused deviation cross-sections for $\delta = 0.05, 0.10, 0.15,$ and 0.20. These deterministic perturbations are not complete models of experimental measurement uncertainty.

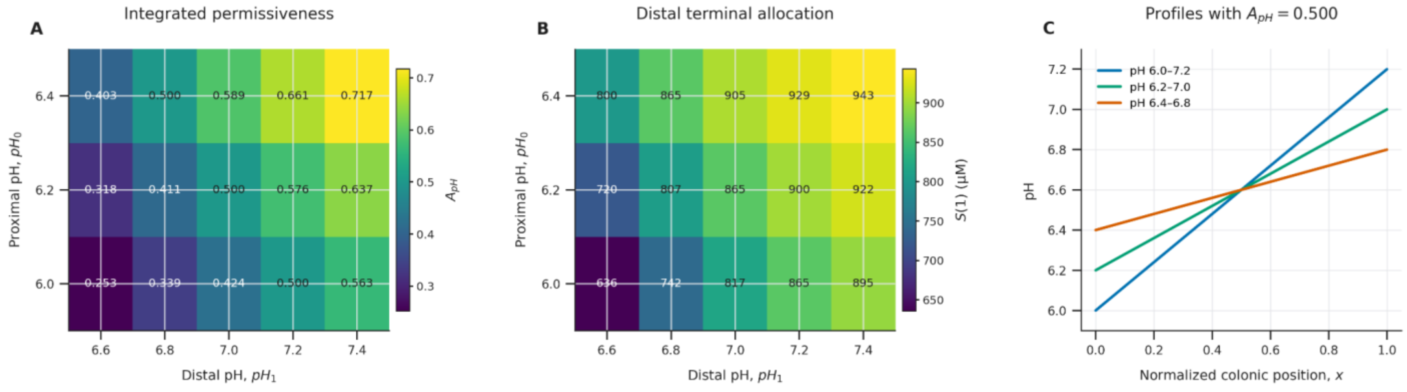

Supplementary Figure S4. Linear pH-profile grid and equal-integrated-permissiveness examples. (A) Integrated permissiveness A_{pH} for selected linear proximal and distal pH combinations. (B) Corresponding distal terminal allocation $S(1)$. (C) Illustrative linear profiles having $A_{\mathrm{pH}} = 0.500$. Broader nonlinear, reversed, and exposure-matched profile results are reported in Supplementary Table S4B and its machine-readable source file. The $A_{\mathrm{pH}} = 0.500$ examples are illustrative equal-exposure cases and are distinct from the linear pH $6.2 - 7.4$ reference profile, for which $A_{\mathrm{pH}} = 0.636678$.

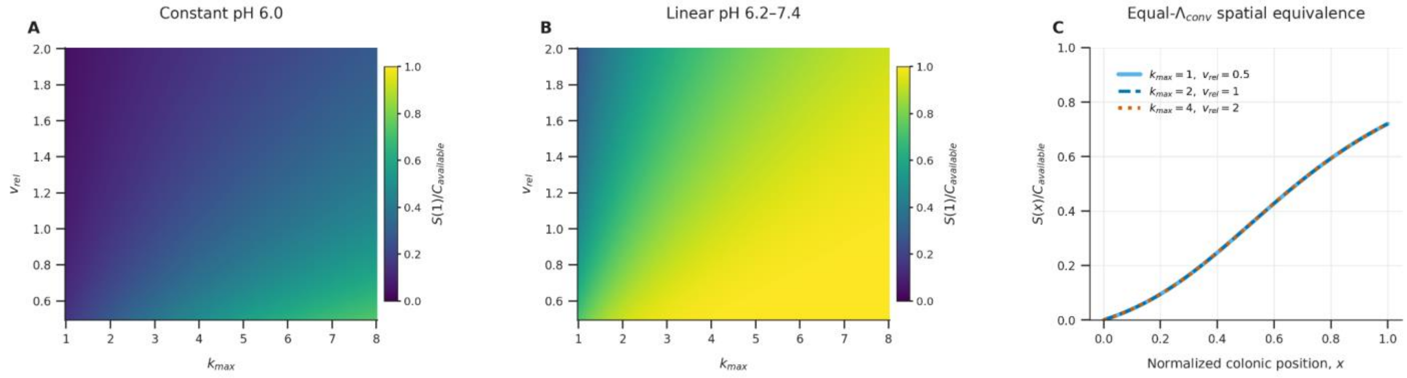

Supplementary Figure S5. Conversion-capacity–relative-progression response surfaces and equal-Λ_{conv} spatial equivalence. (A) Distal terminal allocation under the constant pH 6.0 profile across $k_{\text{max}} = 1.00 - 8.00$ and $v_{\text{rel}} = 0.50 - 2.00$. (B) Corresponding response surface under the linear pH $6.2 - 7.4$ profile. (C) Spatial equivalence for distinct combinations of k_{max}, L_2, and v_{rel} that yield the same composite conversion–progression coefficient, $\Lambda_{\text{conv}} = k_{\text{max}} L_2 / v_{\text{rel}}$. L_2, representing the Terminal Metabolic Lock, was fixed at 1 in panels A and B. L_3, representing the Host-Protection Lock and operationally defined as the dimensionless substrate-availability coefficient, was fixed at 1 in all panels. Thus, these panels evaluate conversion-capacity and relative-progression effects under a fully available model-defined precursor pool. Equal-Λ_{conv} output equivalence is a structural property of the specified loss-free formulation and does not imply biological interchangeability of the underlying mechanisms.

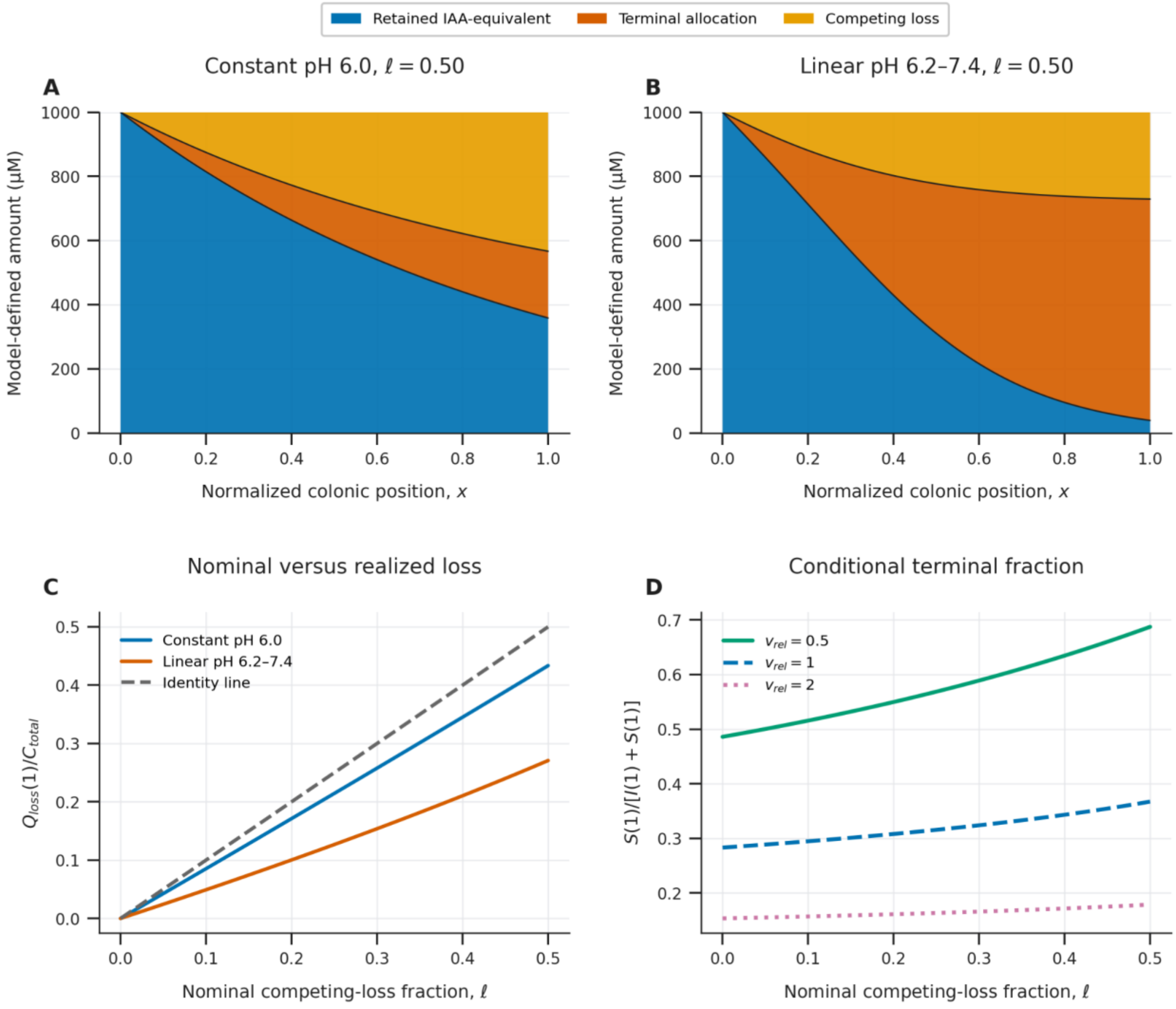

Supplementary Figure S6. Spatial and endpoint behavior under competing loss. (A and B) Spatial distributions of retained indole-3-acetic acid (IAA)-equivalent $I(x)$, terminal allocation $S(x)$, and competing loss $Q_{\text{loss}}(x)$ at nominal $\ell = 0.50$ and $v_{\text{rel}} = 1$ under the two reference pH profiles. (C) Realized loss versus nominal isolated loss. (D) Conditional terminal fraction $S(1)/[I(1) + S(1)]$ across nominal competing-loss fractions under constant pH 6.0 at $v_{\text{rel}} = 0.5, 1$, and 2. Competing loss and conversion draw from the same precursor pool.
